# New Polyhydroxysteroid Glycosides with Antioxidant Activity from the Far Eastern Sea Star *Ceramaster patagonicus*

**DOI:** 10.3390/md22110508

**Published:** 2024-11-10

**Authors:** Timofey V. Malyarenko, Viktor M. Zakharenko, Alla A. Kicha, Arina I. Ponomarenko, Igor V. Manzhulo, Anatoly I. Kalinovsky, Roman S. Popov, Pavel S. Dmitrenok, Natalia V. Ivanchina

**Affiliations:** 1 G.B. Elyakov Pacific Institute of Bioorganic Chemistry, Far Eastern Branch, Russian Academy of Sciences, Pr. 100-let Vladivostoku 159, 690022 Vladivostok, Russia; rarf247@gmail.com (V.M.Z.); kicha@piboc.dvo.ru (A.A.K.); kaaniw@piboc.dvo.ru (A.I.K.); prs_90@mail.ru (R.S.P.); paveldmt@piboc.dvo.ru (P.S.D.); 2 Department of Chemistry and Materials, Institute of High Technology and Advanced Materials, Far Eastern Federal University, Russky Island, Ajax Bay, 10, 690922 Vladivostok, Russia; 3 A.V. Zhirmunsky National Scientific Center of Marine Biology, Far Eastern Branch, Russian Academy of Sciences, ul. Palchevskogo 17, 690041 Vladivostok, Russia; arina.ponomarenko.93@mail.ru (A.I.P.); i-manzhulo@bk.ru (I.V.M.)

**Keywords:** glycosides of polyhydroxysteroids, starfish, *Ceramaster patagonicus*, NMR and MS spectra, antioxidant activity

## Abstract

Four new glycosides of polyhydroxysteroids, ceramasterosides A, B, D, and E (**1**–**4**), and two previously known compounds, ceramasteroside C_1_ (**5**) and attenuatoside B-I (**6**), were isolated from an extract of a deep-sea sea star species, the orange cookie star *Ceramaster patagonicus*. The structures of **1**–**4** were elucidated by the extensive NMR and ESIMS methods. Steroid monoglycosides **1** and **2** had a common 3β,6α,8,15β,16β-pentahydroxysteroid nucleus and a C–29 oxidized stigmastane side chain and differed from each other only in monosaccharide residues. Ceramasteroside A (**1**) contained 3-*O*-methyl-4-*O*-sulfated β-D-xylopyranose, while ceramasteroside B (**2**) had 3-*O*-methyl-4-*O*-sulfated β-D-glucopyranose, recorded from starfish-derived steroid glycosides for the first time. Their biological activity was studied using a model of lipopolysaccharide-induced (LPS) inflammation in a SIM-A9 murine microglial cell line. During the LPS-induced activation of microglial cells, **1**, **3**, and **5**, at a non-toxic concentration of 1 µM, showed the highest efficiency in reducing the production of intracellular NO, while **4** proved to be most efficient in reducing the extracellular nitrite production. All the test compounds reduced the LPS-induced malondialdehyde (MDA) production. The in vitro experiments have demonstrated, for the first time, the antioxidant activity of the compounds under study.

## 1. Introduction

Marine organisms, along with terrestrial plants and microorganisms, represent a promising source of natural biologically active compounds that can potentially be used as medicinal drugs. Sea stars (class Asteroidea, phylum Echinodermata), being widespread benthic predators and detritivores, are considered an important source of various low-molecular-weight metabolites. The chemical diversity and pharmacological potential of sea stars have been studied for more than 50 years. The most common and best-known compounds from sea stars are oxidized steroid metabolites, which have been isolated from almost all studied species. Depending on the chemical structure, these are usually divided into three types: (1) polyhydroxysteroids that have several hydroxy groups in their molecules; (2) polyhydroxysteroid glycosides containing one or more monosaccharide residues; and (3) asterosaponins, a class of steroid oligoglycosides [1,2,3,4,5,6,7,8]. Habitats of sea stars create favorable conditions for including additional structural fragments in their secondary metabolites. Thus, polar steroid compounds often contain a sodium sulfate group in the steroid core, side chain, or monosaccharide residues and show a significant pharmacological potential. A wide range of their biological activities such as hemolytic and embryotoxic, antibacterial and antifungal, anti-inflammatory, immunomodulatory, anticancer, and neuritogenic was studied previously [1,2,3,4,5,6,7,8].

Neurodegenerative diseases, with their prevalence increasing each year, are one of the major human health problems worldwide. Their treatment is a challenge for modern medicine and pharmacology. This is explained, in part, by the fact that the nervous tissue consists not only of neurons that distribute nerve impulses but also of glial cells (astrocytes, oligodendrocytes, microglia cells, and Schwann cells) whose functions are very diverse [9]. In particular, microglia are actually immune cells of the central nervous system that contribute to its homeostasis during development, adulthood, and aging [10]. In addition, microglia cells protect neurons from damage and infection by absorbing dead cells and debris and are also activated in many neurodegenerative diseases [9,10].

To date, effective agents for the treatment of neurodegenerative diseases have also been searched for among various marine natural compounds. Unfortunately, data on the neuritogenic activity of polar steroid compounds from sea stars are scarce. Previously, a group of Japanese researchers published a series of studies on the structures and neuritogenic activity of polyhydroxysteroid glycosides, linckosides A, B–K, and M–Q, echinasteroside C, desulfated echinasterosides A and B, and granulatoside A, isolated from the sea star *Linckia laevigata*, toward rat pheochromocytoma PC12 cells [11,12,13,14,15]. All the considered glycosides demonstrated pronounced neuritogenic activity by inducing the differentiation of rat pheochromocytoma PC12 cells, similarly to the nerve growth factor NGF, and showed a significant synergistic effect on neuronal differentiation of PC12 cells induced by trace amounts of NGF (1.5 ng/mL). In addition, the effect of linckosides A and B and granulatoside A on the morphological properties, proliferation, and migration of human olfactory ensheathing cells (hOECs) was assessed, and these glycosides or related compounds showed a potential to be used to regulate and enhance the therapeutic properties of OECs [16]. Recently, this group of researchers studied the effects of novel steroidal glycosides, acanthasterosides A_1_, B_1_, and B_3_, isolated from the sea star *Acanthaster planci*, on the rat pheochromocytoma cell line PC12 and concluded that structural features influence the activity of these glycosides. They also assessed the effect of acanthasteroside B_3_ on attenuating cognitive impairment in senescence-accelerated mice (SAMP8) in two cognitive tests and demonstrated that acanthasteroside B_3_ could be transported into the brain via the circulatory system in mice [17].

The neuritogenic activity of linckosides L1, L2, and echinasteroside C, isolated from another population of the sea star *L. laevigata*, was studied on a mouse neuroblastoma cell line (NB C-1300) and an organotypic culture of rat hippocampal slices [18]. Intravital observations of the cells showed that these compounds, at a dose of 5 μM, increased the percentage of differentiation after a 4-day incubation to the same extent as nerve growth factor (NGF) at a concentration of 10 ng/mL after a similar exposure. In another study on the NB C-1300 cell line, polyhydroxysteroid glycosides, distolasterosides D_1_–D_3_ from the sea star *Distolasterias nipon* and asterosaponin P_1_, (25S)-5α-cholestane-3β,4β,6α,7α,8,15α,16β,26-octol, and (25S)-5α-cholestane-3β,6α,7α,8,15α,16β,26-heptol from *Patiria pectinifera* at low concentrations showed the ability to stimulate differentiation of NB C-1300 cells, i.e., to increase the number of cells bearing more than two neurites or having neurites longer than two cell diameters [19]. In addition, some of these compounds (at a concentration of 1 μM) exhibited neuroprotective activity on an organotypic rat hippocampal slice culture [19].

Continuing our research on biologically active steroid compounds from the deep-sea orange cookie star *Ceramaster patagonicus* (order Valvatida, family Goniasteridae), collected in the Sea of Okhotsk near Iturup Island, we carried out a study on the aisolation and elucidation of structures of four new glycosides of polyhydroxysteroids, ceramasterosides A, B, D, and E (**1**–**4**), and two previously known compounds, ceramasteroside C_1_ (**5**) and attenuatoside B-I (**6**). In addition, we assessed their antioxidant activity toward the murine microglial cell line SIM-A9 (CRL-3265).

## 2. Results and Discussion

### 2.1. Isolation and Structure Elucidation of 1–4 from C. patagonicus

**Figure 1:**
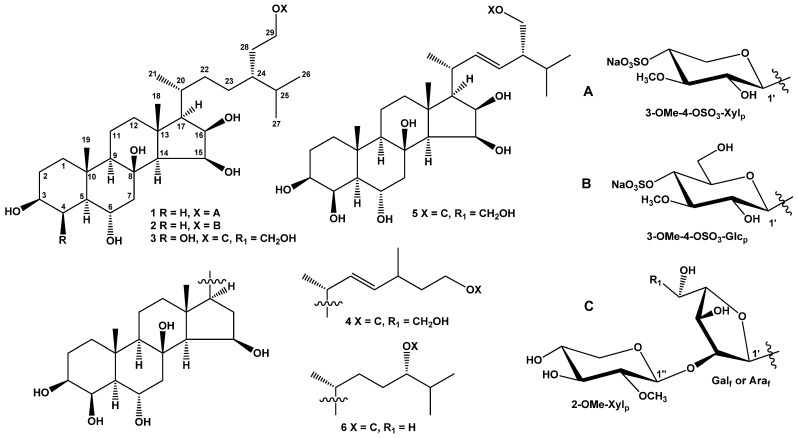
The structures of **1**−**6** isolated from *C. patagonicus*.

The concentrated MeOH/CHCl_3_/EtOH extract of *C. patagonicus* was partitioned between H_2_O and AcOEt/*n*-BuOH, and the organic layer was washed with cold Me_2_CO. The Me_2_CO-insoluble part was subjected to separation by chromatography on silica gel and Florisil columns, followed by high-performance liquid chromatography (HPLC) on semi-preparative Diasfer-110-C18 and Discovery C18 columns and an analytical YMC-Pack Pro C18 column to obtain four new steroid glycosides referred to as ceramasterosides A, B, D, and E (**1**–**4**) and two previously known steroid glycosides (**5** and **6**) (Figure 1). Glycosides **5** and **6** were identified by comparing their ^1^H, ^13^C NMR, and MS spectra with those reported for ceramasteroside C_1_ isolated from *C. patagonicus* earlier [20] and attenuatoside B-I from the sea star *Hacelia attenuata* [21].

**Figure 2:**
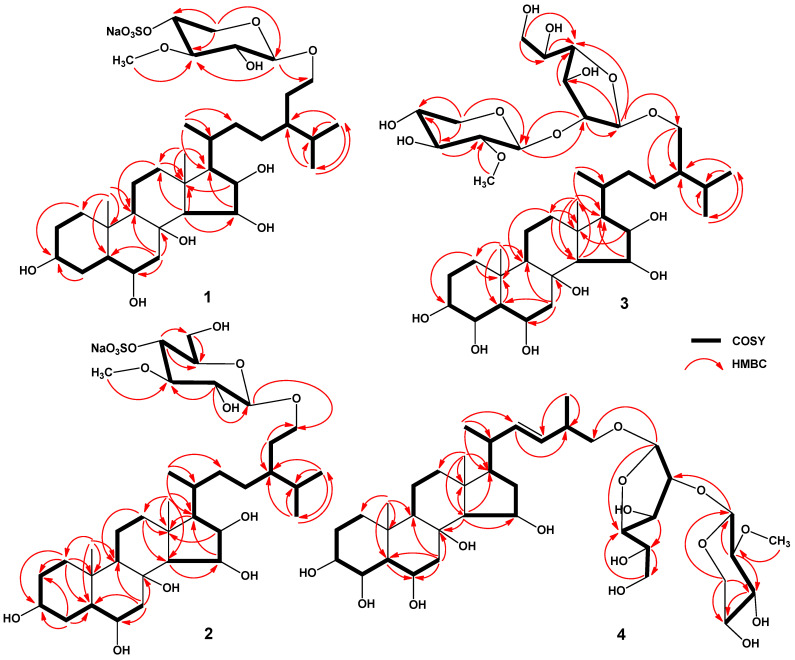
^1^H-^1^H COSY and key HMBC correlations of **1**–**4**.

The molecular formula of **1** was determined to be C_35_H_61_O_13_SNa from the [M + Na]^+^ sodium adduct ion peak at *m/z* 767.3630 [M + Na]^+^ (calculated for [C_35_H_61_O_13_SNa_2_]^+^, 767.3623) in the (+)HRESIMS spectrum and from the ion peak at *m/z* 721.3851 [M − Na]^−^ (calculated for [C_35_H_61_O_13_S]^−^, 721.3838) in the (−)HRESIMS spectrum (Figures S1 and S2). The fragment peaks at *m*/*z* 647 [(M + Na) − NaHSO_4_]^+^ in the (+)ESIMS/MS of the ion [M + Na]^+^ at *m*/*z* 767, as well as at *m*/*z* 97 [HSO_4_]^−^ in the (−)ESIMS/MS of the ion [M − Na]^−^ at *m*/*z* 721, indicated the presence of a sodium sulfate group in **1** (Figure S3). The IR spectrum of **1** showed the presence of the hydroxy (3440 cm^−1^) and sulfate (1255, 1224, 827 cm^−1^) groups (Figure S4). The ^1^H and ^13^C NMR spectroscopic data belonging to the steroid core of **1** showed the resonances of protons and carbons of two angular methyls, CH_3_-18 and CH_3_-19 (*δ*_H_ 1.24 s, 0.98 s; *δ*_C_ 17.9, 14.0), four oxygenated methines, HC-3 (*δ*_H_ 3.47 m; *δ*_C_ 72.2), HC-6 [*δ*_H_ 3.71 td (*J* = 11.0, 4.4); *δ*_C_ 67.7], HC-15 [*δ*_H_ 4.36 dd (*J* = 6.8, 5.7); *δ*_C_ 71.2], and HC-16 (*δ*_H_ 4.22 m; *δ*_C_ 72.8), and a tertiary carbon atom bonded to oxygen at C-8 (*δ*_C_ 77.2) (Table 1, Figures S5 and S6). The NMR spectra of the steroid side chain indicated the existence of three secondary methyls, CH_3_-21 [*δ*_H_ 0.94 d (*J* = 6.6); *δ*_C_ 18.5], CH_3_-26 [*δ*_H_ 0.87 d (*J* = 6.7); *δ*_C_ 20.0], and CH_3_-27 [*δ*_H_ 0.84 d (*J* = 6.7); *δ*_C_ 19.1), and one oxygenated methylene, H_2_C-29 (*δ*_H_ 3.83 m, 3.54 m; *δ*_C_ 70.0) (Table 1, Figures S5 and S6). The ^1^H-^1^H COSY and HSQC correlations attributable to the steroid moiety revealed the corresponding sequences of protons at C-1 to C-7; C-9 to C-12 through C-11; C-14 to C-17; and C-17 to C-21, C-20 to the end of the side chain, and C-24 to C-29 (Figure 2, Figures S7 and S8). The key HMBC cross-peaks, such as H-1/C-3; H-4/C-3; H-7/C-5, C-6, C-8, C-9; H-12/C-9, C-13; H-14/C-12, C-13, C-15; H-15/C-17; H-16/C-13; H_3_-18/C-12, C-13, C-17; H_3_-19/C-1, C-5, C-9, C-10; H_3_-21/C-17, C-20, C-22; H_3_-26/C-24, C-25, C-27; and H_3_-27/C-24, C-25, C-26 confirmed the overall structure of the steroid part of **1** (Figure 2 and Figure S9).

**Figure 3:**
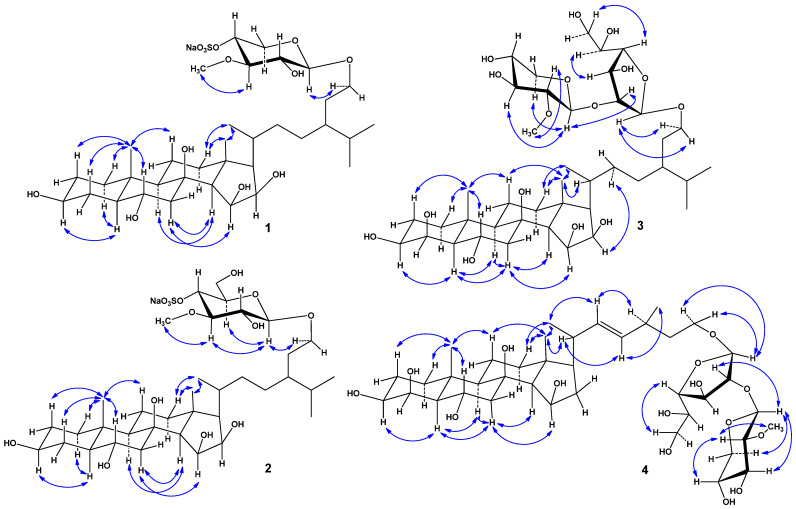
Key ROESY correlations of **1**–**4**.

The key ROESY cross-peaks showed the common 5α/9α/10β/13β stereochemistry of the steroid nucleus, 3β,6α,15β,16β-configurations of oxygenated substituents, and 29-hydroxystigmastane side chain in **1** (Figure 3 and Figure S10). The ^1^H and ^13^C NMR data of the steroid part of **1** were almost identical to those of halityloside B from *Halityle regularis* [22] with 24-ethyl-5α-cholestane-3β,6α,8,15β,16β,29-hexol as aglycon. The 20*R* configuration was assumed on the basis of the chemical shift of H_3_-21 at *δ* 0.94 (more than *δ* 0.90 for 20*R*-steroids with saturated side chain [23]) and ROESY correlations of H_3_-18/H_3_-21 and H_3_-21/H_β_-12 (Figure 3 and Figure S10). The 24*R* configuration in **1** was determined to be similar to halityloside B based on the values Δ*δ_H_* = 0.03 between the H_3_C-26 and H_3_C-27 proton signals and the values Δ*δ_C_* = 0.9 between the C–26 and C–27 carbon signals (Table 1) [22]. On the basis of the above data, the steroid moiety of **1** was determined as (20*R*,24*R*)-24-ethyl-5α-cholestane-3β,6α,8,15β,16β,29-hexol.

In addition to the above-mentioned signals, the ^1^H NMR spectrum of **1** exhibited a resonance in the deshielded region due to the anomeric proton of the monosaccharide unit at *δ_H_* 4.23, which correlated in the HSQC experiment with carbon signals at *δ*_C_ 104.8, and also one resonance due to an *O*-methyl group at *δ*_H_ 3.61, which correlated in the HSQC experiment with a carbon signal at *δ*_C_ 60.7 (Table 2, Figures S5 and S6). The presence of a monosaccharide unit in glycoside **1** was confirmed by ESIMS/MS data. In fact, the (−)ESIMS/MS spectrum of the ion [M − Na]^−^ at *m/z* 721 showed a fragment peak obtained due to the loss of a sugar unit from the decationized molecule at *m/z* 495 [(M − Na) − C_6_H_10_O_7_S]^−^ and a fragment peak of monosaccharide residue at *m/z* 225 [C_6_H_9_O_7_S]^−^ (Figure S3). Thus, the sodium sulfate group is localized in the monosaccharide residue of glycoside **1** and the monosaccharide residue itself was determined to be *O*-methyl-sulfoxy-pentose. The ^1^H-^1^H COSY and HSQC correlations attributable to the monosaccharide unit revealed the corresponding sequences of protons at C-1′–C-5′ (Figure 2, Figures S7 and S8). The key HMBC correlations H-1′/C-3′; H-3′/O-CH_3_; H-5′/C-1′, C-3′, and C-4′ and the ROESY correlation H-3′/O-CH_3_ supported the total structure of the 3-*O*-methyl-4-sulfoxy-xylopyranosyl residue (Figure 2, Figure 3, Figures S9 and S10). Moreover, the localization of the methyl group at C-3′ was indicated by the downfield chemical shift of C-3′ due to the α-effect of methylation in the ^13^C NMR spectrum. However, we noticed that the signal of C-3′ was in the upfield shift *δ*_C_ 85.1 compared to the signal of C-3′ *δ*_C_ 87.5 in 3-*O*-methyl-xylose residues [24]. The same effect was observed for the signal of C-5′ *δ*_C_ 64.8 instead of *δ*_C_ 66.7 [24]. Furthermore, C-4′ had a marked chemical downfield shift relative to *δ*_C_ 70.9 for non-sulfated 3-*O*-methyl-xylose to *δ*_C_ 77.2 in the monosaccharide residue of **1**. This indicated the localization of the sodium sulfate group at C-4′ of the 3-*O*-methyl-xylose moiety of glycoside **1** and was confirmed by data from ^1^H NMR spectra where a noticeable downfield shift of signals of H-3′, H-4′, and H-5′ was observed compared to the non-sulfated 3-*O*-methyl-xylose residue (Table 2, Figures S5 and S6). The coupling constant (7.3 Hz) of the anomeric proton corresponded to a β-configuration of the glycoside bond. The D-series of the monosaccharide unit was expected to be similar to the co-occurring glycosides previously isolated from the same sea star species [20]. 3-*O*-methyl-4-*O*-sulfo-β-D-xylopyranose is a rare monosaccharide residue in starfish-derived steroid glycosides that was previously found in scoparioside C from the sea star *Astropecten scoparius* [25]. The attachment of the monosaccharide residue to C-29 of steroid aglycon was inferred from the HMBC and ROESY spectra, where cross-peaks between H-1′ of 3-OMe-4-OSO_3_-Xyl and C-29/H-29 of aglycon, respectively, were observed (Figure 2, Figure 3, Figures S9 and S10). Based on these results, the structure of ceramasteroside A (**1**) was determined as (20*R*,24*R*)-29-*O*-(3-*O*-methyl-4-*O*-sulfo-β-D-xylopyranosyl)-24-ethyl-5α-cholestane-3β,6α,8,15β,16β,29-hexol sodium salt (**1**).

The molecular formula of **2** was determined to be C_36_H_63_O_14_SNa from the sodium adduct ion peak at *m/z* 797.3738 [M + Na]^+^ (calculated for [C_36_H_63_O_14_SNa_2_]^+^, 797.3728) in the (+)HRESIMS spectrum and from the ion peak at *m/z* 751.3946 [M − Na]^−^ (calculated for [C_36_H_63_O_14_S]^−^, 751.3944) in the (−)HRESIMS spectrum (Figures S11 and S12). The fragment peaks at *m*/*z* 677 [(M + Na) − NaHSO_4_]^+^ in the (+)ESIMS/MS of the ion at *m*/*z* 797 [M + Na]^+^, as well as at *m*/*z* 97 [HSO_4_]^−^ in the (−)ESIMS/MS of the ion at *m*/*z* 751 [M − Na]^−^, indicated the presence of a sodium sulfate group in **2** (Figure S13). The IR spectrum of **2** showed the presence of hydroxy (3358 cm^−1^) and sulfate (1255, 1224, 827 cm^−1^) groups (Figure S4). The comparison of the molecular masses of **2** and **1** showed that the difference between **2** and **1** is 30 amu (CH_2_O). The ^1^H and ^13^C NMR data of **2** differed from those of **1** only in the signals of the monosaccharide residue and the proton H-29 (Table 1 and Table 2). The ^1^H NMR spectrum of **2** exhibited one resonance in the deshielded region due to an anomeric proton of monosaccharide unit at *δ_H_* 4.27, which correlated in the HSQC experiment with a carbon signal at *δ_C_* 104.4, and also one resonance due to *O*-methyl protons of monosaccharide unit at *δ_H_* 3.62, correlated in the HSQC experiment with a carbon signal at *δ_C_* 61.1 (Table 2, Figures S15 and S16). The (−)ESIMS/MS spectrum of the [M − Na]^−^ ion at *m/z* 751 showed a fragment peak obtained due to the loss of a sugar unit from a decationized molecule at *m/z* 495 [(M − Na) − C_7_H_12_O_8_S]^−^ and a fragment peak of monosaccharide residue at 255 [C_7_H_11_O_8_S]^−^ (Figure S13). Thus, the sodium sulfate group is localized in the monosaccharide unit of glycoside **2**, and the monosaccharide moiety itself was determined to be *O*-methyl-sulfoxy-hexose. The chemical shifts and coupling constants of H-1′−H-6′ and chemical shifts of C-1′−C-6′ of an *O*-methyl-sulfoxy-hexose unit were inferred from the COSY and HSQC experiments (Table 2, Figures S17 and S18). The HMBC correlations H-2′/C-1′, C-3′; O-CH_3_/C-3′; H-4′/C-5′, C-6′; and H-6′/C-5′ and ROESY correlations H-1′/H-3′, H-5′, and H-3′/O-CH_3_ supported the total structure of the 3-*O*-methyl-4-*O*-sulfo-glucopyranosyl residue (Figure 2, Figure 3, Figures S19 and S20). The coupling constant (7.5 Hz) of the anomeric proton corresponded to a β-configuration of the glycoside bond. The attachment of the monosaccharide residue to C-29 of steroid aglycon was inferred from the HMBC and ROESY spectra, where cross-peaks between H-1′ of 3-OMe-4-OSO_3_-Glc and C-29/H-29 of aglycon, respectively, were observed (Figure 2, Figure 3, Figures S19 and S20). As far as we know, 3-*O*-methyl-4-*O*-sulfo-glucopyranose was not previously found in starfish steroid glycosides. Accordingly, the structure of ceramasteroside B (**2**) was determined as (20*R*,24*R*)-29-*O*-(3-*O*-methyl-4-*O*-sulfo-β-D-glucopyranosyl)-24-ethyl-5α-cholestane-3β,6α,8,15β,16β,29-hexol sodium salt (**2**).

The molecular formula of **3** was determined to be C_41_H_72_O_16_ from the sodium adduct ion peak at *m/z* 843.4715 [M + Na]^+^ (calculated for [C_41_H_72_O_16_Na]^+^, 843.4713) in the (+)HRESIMS spectrum and from the peak of the deprotonated molecule *m/z* 819.4748 [M − H]^−^ (calculated for [C_41_H_71_O_16_]^−^, 819.4748) in the (−)HRESIMS spectrum (Figures S21 and S22). The IR spectrum of **3** showed the presence of a hydroxy (3323 cm^−1^) group (Figure S24). The ^1^H and ^13^C NMR spectroscopic data belonging to the steroid core of **3** showed resonances of protons and carbons of two angular methyls, CH_3_-18 and CH_3_-19 (*δ*_H_ 1.23 s, 1.15 s; *δ*_C_ 17.9, 17.0), five oxygenated methines, HC-3 (*δ*_H_ 3.42 m; *δ*_C_ 73.7), HC-4 (*δ*_H_ 4.26 brs; *δ*_C_ 69.1), HC-6 [*δ*_H_ 4.18 td (*J* = 11.2, 3.5); *δ*_C_ 64.8], HC-15 [*δ*_H_ 4.37 dd (*J* = 6.8, 5.6); *δ*_C_ 71.2], and HC-16 [*δ*_H_ 4.22 t (*J* = 6.8); *δ*_C_ 72.8], and a tertiary carbon atom bonded to oxygen at C-8 (*δ*_C_ 77.2) (Table 1, Figures S25 and S26). The NMR spectra of the steroid side chain indicated the existence of three secondary methyls, CH_3_-21 [*δ*_H_ 0.95 d (*J* = 6.6); *δ*_C_ 18.6], CH_3_-26 [*δ*_H_ 0.87 d (*J* = 6.8); *δ*_C_ 20.1], and CH_3_-27 [*δ*_H_ 0.85 d (*J* = 6.8); *δ*_C_ 19.0], and one oxygenated methylene, H_2_C-29 (*δ*_H_ 3.73 m, 3.43 m; *δ*_C_ 68.0) (Table 1, Figures S25 and S26). The ^1^H and ^13^C NMR data of the steroid moiety of **3** differed from those of **1** only in the signals of the ring A of the steroid nucleus (Table 1 and Table 2). Thus, in glycoside **3**, there was an additional hydroxy group at C-4. Hence, the ^1^H and ^13^C NMR data of the steroid part of **3** were almost identical to those of halityloside A from *Halityle regularis* [22] with 24-ethyl-5α-cholestane-3β,4β,6α,8,15β,16β,29-heptol as aglycon.

In addition to the above-mentioned signals, the ^1^H NMR spectrum of **3** exhibited two resonances in the deshielded region due to the anomeric protons of monosaccharide units at *δ_H_* 5.00 and 4.45, which correlated in the HSQC experiment with carbon signals at *δ*_C_ 108.0 and 104.7, and also one resonance due to an *O*-methyl group at *δ*_H_ 3.58, which correlated in the HSQC experiment with a carbon signal at *δ*_C_ 61.2 (Table 2, Figures S25 and S26). The presence of two monosaccharide units in glycoside **3** were confirmed by ESIMS/MS data. In fact, the (−)ESIMS/MS spectrum of the deprotonated molecule [M − H]^−^ at *m/z* 819 showed fragment peaks obtained due to the losses of sugar units from deprotonated molecule at *m/z* 655 [(M − H) − C_6_H_10_O_4_ − H_2_O]^−^ and 511 [(M − H) − C_6_H_10_O_4_ − C_6_H_10_O_5_]^−^ (Figure S23). Thus, the monosaccharide residues were determined to be terminal *O*-methyl-pentose and hexose. The ^1^H-^1^H COSY and HSQC correlations attributable to monosaccharide units revealed the corresponding sequences of protons at C-1′–C-5′ of *O*-methyl-pentose and C-1′–C-6′ of hexose (Figure 2, Figures S27 and S28). The key HMBC and ROESY correlations supported the total structure of the terminal 3-*O*-methyl-xylopyranosyl and galactofuranosyl residues (Figure 2, Figure 3, Figures S29 and S30). The coupling constant (*J* = 7.7 Hz) of the anomeric protons of 3-OMe-Xyl_p_ corresponded to a β-configurations of the glycoside bond. The chemical shift of the anomeric carbon of Gal_f_ at *δ*_C_ 108.0 corresponded to the β-configuration of the glycosidic bond (δ_C_ 109.2 for β-*O*-methylgalactofuranose and *δ*_C_ 103.1 for α-*O*-methylgalactofuranose [26]). The attachment of the monosaccharide residues to C-29 of steroid aglycon and each other was inferred from the HMBC and ROESY spectra, where cross-peaks between H-1′‘ of 3-OMe-Xyl_p_ and C-2′/H-2′ of Gal_f_ and H-1′ of Gal_f_ and C-29/H_2_-29 of aglycon, respectively, were observed (Figure 2, Figure 3, Figures S29 and S30). This carbohydrate fragment was previously found in ceramasterosides C_1_, C_3_, and C_4_ from *C. patagonicus* [20]. The D-series of monosaccharide units was expected to be similar to the co-occurring glycosides previously isolated from the same sea star species [20]. Based on these results, the structure of ceramasteroside D (**3**) was determined as (20*R*,24*R*)-29-*O*-(3-*O*-methyl-β-D-xylopyranosyl-(1→2)-β-D-galactofuranosyl)-24-ethyl-5α-cholestane-3β,4β,6α,8,15β,16β,29-heptol (**3**).

The molecular formula of **4** was determined to be C_39_H_66_O_15_ from the sodium adduct ion peak at *m/z* 797.4295 [M + Na]^+^ (calculated for [C_39_H_66_O_15_Na]^+^, 797.4294) in the (+)HRESIMS spectrum and from the peak at *m/z* 773.4337 [M − H]^−^ (calculated for [C_39_H_65_O_15_]^−^, 773.4329) in the (−)HRESIMS spectrum (Figure S31 and S32). The IR spectrum of **4** showed the presence of a hydroxy groups (3365 cm^−1^) (Figure S34). The ^1^H and ^13^C NMR spectroscopic data belonging to the steroid core of **4** showed the resonances of protons and carbons of two angular methyls, CH_3_-18 and CH_3_-19 (*δ*_H_ 1.27 s, 1.15 s; *δ*_C_ 16.7, 17.0), four oxygenated methines, HC-3 (*δ*_H_ 3.42 m; *δ*_C_ 73.7), HC-4 (*δ*_H_ 4.25 brs; *δ*_C_ 69.1), HC-6 (*δ*_H_ 4.15 m; *δ*_C_ 64.8), and HC-15 [*δ*_H_ 4.39 td (*J* = 7.2, 2.0); *δ*_C_ 71.2], and a tertiary carbon atom bonded to oxygen at C-8 (*δ*_C_ 77.4) (Table 1, Figures S35 and S36). The NMR spectra of steroid side chain indicated the existence of two secondary methyls, CH_3_-21 [*δ*_H_ 0.99 d (*J* = 6.6); *δ*_C_ 21.2] and CH_3_-28 [*δ*_H_ 0.96 d (*J* = 6.7); *δ*_C_ 21.7], an oxygenated methylene, H_2_C-26 (*δ*_H_ 3.70 m, 3.39 m; *δ*_C_ 67.2), and a ∆^22^ double bond [*δ*_H_ 5.21 dd (*J* = 15.1, 8.6), 5.15 dd (*J* = 15.1, 7.8); *δ*_C_ 137.2, 134.4] (Table 1, Figures S35 and S36). The ^1^H-^1^H COSY and HSQC correlations attributable to steroid moiety revealed the corresponding sequences of protons at C-1 to C-7; C-9 to C-12 through C-11; C-14 to C-17; and C-17 to C-21, C-20 to the end of the side chain, and C-24 to C-28 (Figure 2, Figures S37 and S38). The key HMBC cross-peaks such as H-7/C-5, C-6, C-8, C-9; H-14/C-8, C-13, C-15, C-17; H_3_-18/C-12, C-13, C-14, C-17; H_3_-19/C-1, C-5, C-9, C-10; H_3_-21/C-17, C-20, C-22; H-22/C-24; and H_3_-28/C-23, C-24 confirmed the overall structure of the steroid part of **4** (Figure 2 and Figure S39). The key ROESY cross-peaks showed the common 5α/9α/10β/13β stereochemistry of the steroid nucleus, 3β,4β,6α,15β-configurations of oxygenated substituents and ∆^22^-24-methyl-27-*nor*-cholestane side chain in **4** (Figure 3 and Figure S40). ^1^H and ^13^C NMR data of the steroid part of **4** were almost identical to those of placentoside A from *Sphaerodiscus placenta* [27] with 24-methyl-27-*nor*-5α-cholest-22-ene-3β,4β,6α,8,15β,26-hexol as aglycon. The *E*-configuration of the ∆^22^-double bond was inferred from the values of the coupling constants of H-22 and H-23 at 15.1 Hz. On the basis of the above data, the steroid moiety of **4** was determined as (20*R*,22*E*,24*ξ*)-24-methyl-27-*nor*-5α-cholest-22-ene-3β,4β,6α,8,15β,26-hexol.

In addition to the above-mentioned signals, the ^1^H NMR spectrum of **4** exhibited two resonances in the deshielded region due to the anomeric protons of monosaccharide unit at *δ*_H_ 5.00 and 4.45, which correlated in the HSQC experiment with carbon signals at *δ*_C_ 108.0 and 104.7, and one also with resonance due to an *O*-methyl group at *δ*_H_ 3.58, which correlated in the HSQC experiment with a carbon signal at *δ*_C_ 61.2 (Table 2, Figures S35 and S36). Based on the extensive 2D NMR and MS analysis of glycosides **4** and **3**, we suggested that the carbohydrate moiety of **4** was identical to those of **3** (Table 2, Figures S33 and S35–S40). Thus, the structure of ceramasteroside E (**4**) was elucidated to be (20*R*,22*E*,24*ξ*)-26-*O*-(3-*O*-methyl-β-D-xylopyranosyl-(1→2)-β-D-galactofuranosyl)- 24-methyl-27-*nor*-5α-cholest-22-ene-3β,4β,6α,8,15β,26-hexol (**4**).

### 2.2. Biological Activity Assay

#### 2.2.1. Cytotoxicity of Compounds Against Microglial Cells

**Figure 4:**
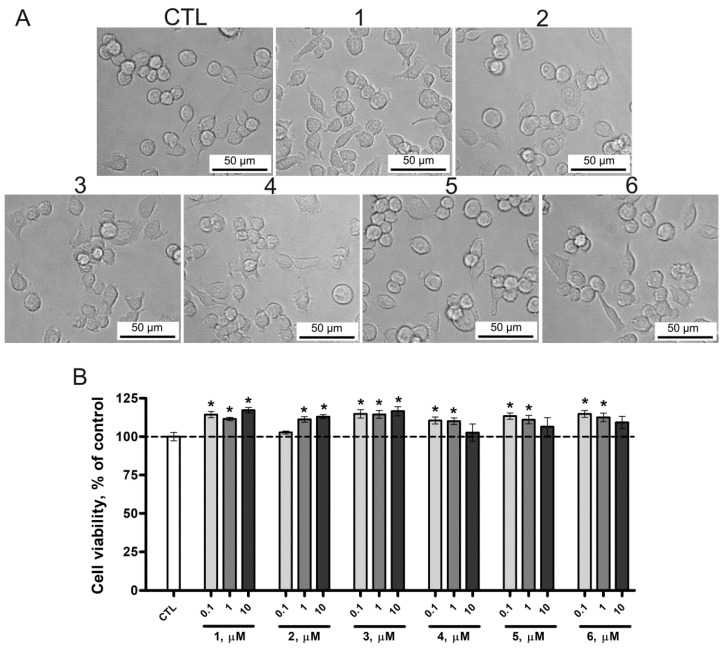
(**A**) Images of microglial cells exposed to lipopolysaccharide (LPS) and **1**–**6** at a concentration of 10 µM. (**B**) The cytotoxic effect of **1**–**6** on mouse microglial cell line SIM-A9 determined by MTS assay. Data are mean ± SEM; *n* = 8 (number of samples analyzed); * *p* < 0.05 vs. control (CTL) (Student’s *t*-test).

In this study, we assessed the effect of **1**–**6** on the viability, proliferation, and morphology of microglial cells. The absence of the cytotoxic effect of the compounds under study toward mouse microglial SIM-A9 cells was confirmed by tetrazolium staining (MTS assay method) with 72-h exposure of cells. We found that the tested concentrations of compounds (0.1, 1, and 10 µM), in addition to the lack of cytotoxic effect, *vice versa*, increased the metabolism of tetrazolium, which may indicate an increase in proliferation and metabolism compared to the control group (Figure 4B). Micrographs of the SIM-A9 culture also showed the absence of any pronounced morphological changes in terms of the characteristics of cells in a state of apoptosis/necrosis such as vacuolization, sagging, and loss of attachment to the substrate (Figure 4A).

#### 2.2.2. Antioxidant Activity of Compounds Toward Microglial Cells

**Figure 5:**
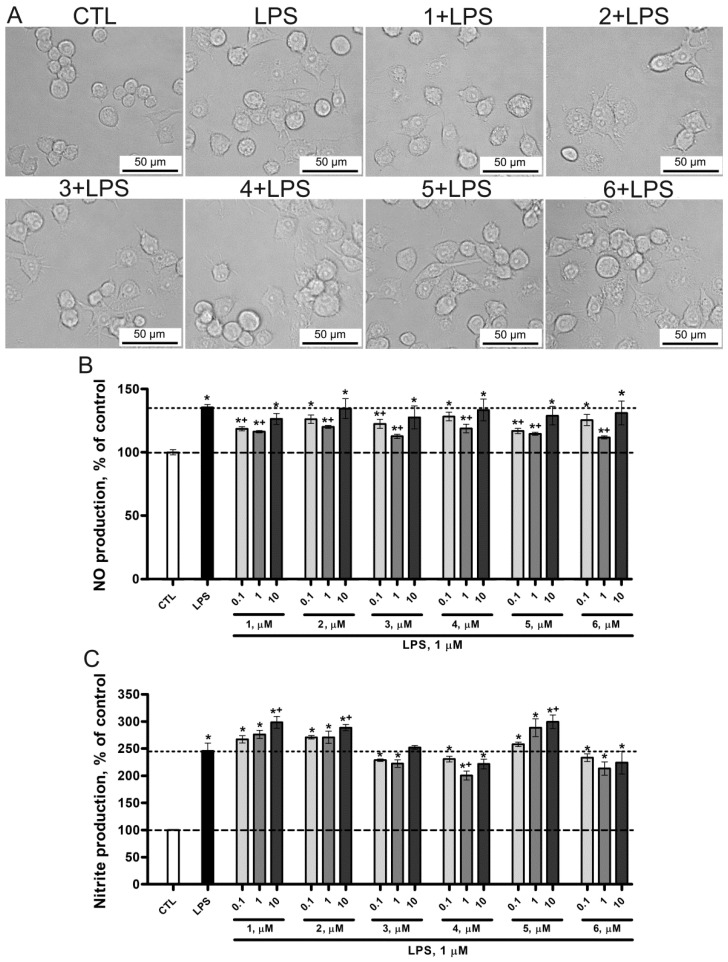
(**A**) Morphological changes in microglia under the exposure to LPS and **1**–**6** at a concentration of 10 µM. Antioxidant activity of **1**–**6** against LPS-induced production of NO (**B**) and nitrites (**C**) in mouse microglial cell line SIM-A9. Data are mean ± SEM; *n* = 8 (number of samples analyzed)’ * *p* < 0.05 vs. CTL, ^+^ *p* < 0.05 vs. LPS (Student’s *t*-test).

One of important responses of glial cells to various pathological processes in nervous tissue is the activation of oxidative molecules such as nitric oxide (NO), nitrites, and ROS, which induces the protective functions of inflammation in the period of acute injury. However, excessive and prolonged inflammation can be neurotoxic due to the release of mediators that trigger vicious cycles of microglia-mediated neurodegeneration [28]. Thus, the search for effective and non-toxic anti-inflammatory and antioxidant compounds is a priority issue to address in biology and medicine. During the LPS-induced activation, microglia are transformed morphologically (from a branched to an amoeboid form) and functionally (begin to secrete free radicals) (Figure 5A).

When studying the LPS-induced intracellular NO production under the exposure to the compounds, we observed the following pattern: the best concentration inhibiting the LPS-induced NO production for all compounds was 1 µM, while the maximum concentration of 10 µM had almost no effect. The highest activity at a minimum concentration of 0.1 µM was exhibited by **1** and **5** (Figure 5B).

The study of nitrite secreted into the culture medium under the exposure to LPS and the test compounds revealed other patterns that differed from that for NO. **1**, 2, and **5** at a concentration of 10 µM, *vice versa*, increased the concentration of extracellular nitrite in the culture medium even above that for LPS. Only **4** at a concentration of 1 µM significantly reduced extracellular nitrite (Figure 5C).

**Figure 6:**
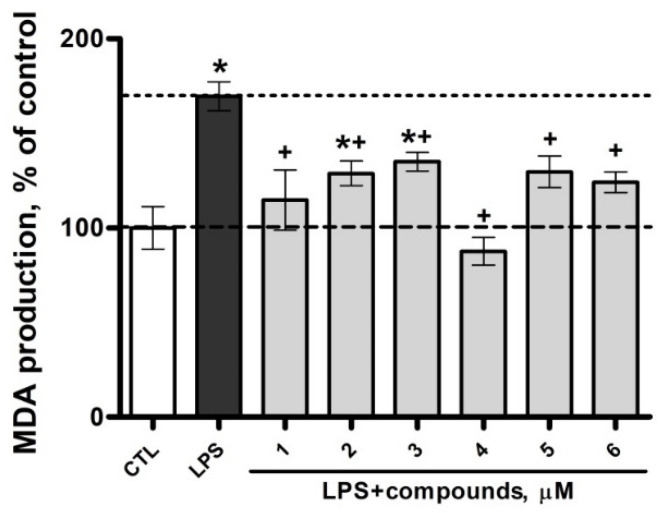
Analysis of MDA production in SIM-A9 cell line under the exposure to LPS and **1**–**6** at a concentration of 1 µM. Data are mean ± SEM; *n* = 8 (number of samples analyzed); * *p* < 0.05 vs. CTL; ^+^ *p* < 0.05 vs. LPS (Student’s *t*-test).

The rate of oxidative stress development was also directly proportional to the intensity of activation of lipid peroxidation processes in microglia, which was manifested as a pronounced increase in the level of malondialdehyde (MDA) in the LPS group, almost two-fold higher vs. the control group. Moreover, all the test compounds decreased the LPS-induced MDA production, while with the use of **1** and **4,** the MDA levels were indistinguishable from the control levels (Figure 6).

## 3. Materials and Methods

### 3.1. General Methods

Optical rotations were measured on a PerkinElmer 343 polarimeter (Waltham, MA, USA). IR spectra were recorded using an IRTracer-100 FTIR Spectrophotometer (Shimadzu, Kyoto, Japan) with a Quest attenuated total reflection (ATR) accessory P/N GS10801-B (Specac Ltd., Orpington, UK). The ^1^H and ^13^C NMR spectra were obtained on a Bruker Avance III 700 spectrometer (Bruker BioSpin, Bremen, Germany) at 700.13 and 176.04 MHz, respectively. Chemical shifts (ppm) were internally referenced to the corresponding solvent signals at *δ*_H_ 3.30/*δ*_C_ 49.0 for CD_3_OD. HRESIMS spectra were recorded on a Bruker Impact II Q-TOF mass spectrometer (Bruker, Bremen, Germany); samples were dissolved in MeOH (*c* 0.001 mg/mL). HPLC separations were carried out on an Agilent 1100 Series chromatograph (Agilent Technologies, Santa Clara, CA, USA) equipped with a differential refractometer and on the following columns: Diasfer-110-C18 (10 μm, 250 × 15 mm, Biochemmack, Moscow, Russia), Discovery C18 (5 μm, 250 mm × 10 mm, Supelco, North Harrison, PA, USA), and YMC-Pack Pro C18 (5 μm, 250 × 4.6 mm, YMC Co., Ltd., Kyoto, Japan). Low-pressure liquid column chromatography was carried out with Polychrom-1 (powdered Teflon, 0.25–0.50 mm; Biolar, Olaine, Latvia), Si gel KSK (50–160 μm, Sorbpolimer, Krasnodar, Russia), and Florisil (60–100 μm, Sigma Aldrich, St. Louis, MO, USA). Sorbfil Si gel plates (4.5 × 6.0 cm, 5–17 μm, Sorbpolimer, Krasnodar, Russia) were used for thin-layer chromatography (TLC).

### 3.2. Animal Material

Specimens of *Ceramaster patagonicus* Sladen, 1889 (order Valvatida, family Goniasteridae), were collected in the Sea of Okhotsk, at a depth of 150–300 m near Iturup Island, during the 42nd scientific cruise aboard the R/V *Akademik Oparin* in August 2012. The species was identified by B.B. Grebnev (G.B. Elyakov Pacific Institute of Bioorganic Chemistry (PIBOC) FEB RAS, Vladivostok, Russia). A voucher specimen (No. 042-67) has been deposited at the marine specimen collection of the PIBOC FEB RAS, Vladivostok, Russia.

### 3.3. Extraction and Isolation

Freshly collected *C. patagonicus* specimens (3 kg wet weight) were cut into small pieces and extracted with CHCl_3_/MeOH (2:1) followed by further extraction with CHCl_3_/MeOH (1:1) and EtOH. The combined extracts were concentrated *in vacuo* to a residue of 159.5 g. This residue was separated between H_2_O (1.5 L) and AcOEt/*n*-BuOH (2:1) (4.5 L), and the organic layer was concentrated *in vacuo* to obtain a less polar fraction (51.5 g), which was washed with cold Me_2_CO (1 L). The Me_2_CO-insoluble fraction (23 g) was chromatographed on a Si gel column (18 × 6 cm) using CHCl_3_, CHCl_3_/MeOH (9:1), and CHCl_3_/MeOH/H_2_O (9:1.5:0.1→8:2:0.2→6:4:0.5→6:4:1, *v*/*v*/*v*) to yield eight fractions: 1 (7.7 g), 2 (10.3 g), 3 (1.4 g), 4 (912 mg), 5 (619 mg), 6 (1.08 g), 7 (1.24 g), and 8 (2.98 g). Fraction 6 was further chromatographed on a Florisil column (6 × 4 cm) using CHCl_3_/MeOH (9:3) and CHCl_3_/MeOH:H_2_O (8:2:0.2→6:4:0.5→6:4:1, *v*/*v*/*v*) to yield two subfractions, 61 (311 mg) and 62 (837 mg), which were then analyzed by TLC in the eluent system *n*-BuOH/EtOH/H_2_O (4:1:2). Fraction 7 was further chromatographed on a Florisil column (9.5 × 4 cm) using CHCl_3_/MeOH/H_2_O (8:2:0.2→6:4:0.5→6:4:1, *v*/*v*/*v*) to yield three subfractions, 71 (180.5 mg), 72 (365 mg), and 73 (406 mg), which were then analyzed by TLC in the eluent system *n*-BuOH/EtOH/H_2_O (4:1:2). Subfractions 61, 62, and 71–73 mainly contained glycosides of polyhydroxysteroids with admixtures of pigments and concomitant lipids. An HPLC separation of subfraction 61 (311 mg) on a Diasfer-110-C18 column (2.5 mL/min) with MeOH/H_2_O (80:20) as an eluent yielded eight subfractions: 61-5 to 61-12. An HPLC separation of subfraction 61-5 on a YMC-Pack Pro C18 column (1.0 mL/min) with MeOH/H_2_O (75:25) as an eluent yielded pure **6** (1.3 mg, *R*t 8.2 min). An HPLC separation of subfraction 71 (180.5 mg) on a Discovery C18 column (2.5 mL/min) with MeOH/H_2_O (70:30) as an eluent yielded pure **1** (3.0 mg, *R*t 18.8 min) and **2** (3.0 mg, *R*t 17.3 min). An HPLC separation of subfraction 72 (365 mg) on a Discovery C18 column (2.5 mL/min) with MeOH/H_2_O (75:25) as an eluent yielded pure **3** (2.0 mg, *R*t 37.7 min), **5** (9.5 mg, *R*t 34.2 min), and subfraction 72-7 (3.5 mg). An HPLC separation of subfraction 72-7 on a YMC-Pack Pro C18 column (1.0 mL/min) with MeOH/H_2_O (65:35) as an eluent yielded pure **4** (2.3 mg, *R*t 31.5 min).

### 3.4. Spectral Data of New Compounds

**Ceramasteroside A** (**1**), C_35_H_61_O_13_SNa, amorphous powder; [α]_D_^25^ +4.2° (c 0.3, MeOH); IR: ν_max_ = 3350, 2928, 2870, 1456, 1379, 1255, 1224, 1074, 1045, 991, 879, 827, 588 cm^−1^; ^1^H and ^13^C NMR data are listed in Table 1 and Table 2; (+)ESIMS/MS of the ion [M + Na]^+^ at *m/z* 767: 647 [(M + Na) − NaHSO_4_]^+^, 629 [(M + Na) − NaHSO_4_ − H_2_O]^+^, 143 [Na_2_HSO_4_]^+^; (+)HRESIMS *m/z* 767.3630 [M + Na]^+^ (calculated for [C_35_H_61_O_13_SNa_2_]^+^, 767.3623); (−)ESIMS/MS of the ion [M − Na]^−^ at *m/z* 721: 495 [(M − Na) − C_6_H_10_O_7_S]^−^; 225 [C_6_H_10_O_7_S]^−^; 97 [HSO_4_]^−^; (−)HRESIMS *m/z* 721.3851 [M − Na]^−^ (calculated for [C_35_H_61_O_13_S]^−^, 721.3838).

**Ceramasteroside B** (**2**), C_36_H_63_O_14_SNa, amorphous powder; [α]_D_^25^ +4.7° (c 0.3, MeOH); IR: ν_max_ = 3358, 2927, 2870, 1456, 1379, 1255, 1224, 1074, 1045, 987, 879, 827, 588 cm^−1^; ^1^H and ^13^C NMR data are listed in Table 1 and Table 2; (+)ESIMS/MS of the ion [M + Na]^+^ at *m/z* 797: 779 [(M + Na) − H_2_O]^+^, 677 [(M + Na) − NaHSO_4_]^+^, 143 [Na_2_HSO_4_]^+^; (+)HRESIMS *m/z* 797.3738 [M + Na]^+^ (calculated for [C_36_H_63_O_14_SNa_2_]^+^, 797.3728); (−)ESIMS/MS of the ion [M − Na]^−^ at *m/z* 751: 495 [(M − Na) − C_7_H_12_O_8_S]^−^; 255 [C_7_H_12_O_8_S]^−^; 97 [HSO_4_]^−^; (−)HRESIMS *m/z* 751.3946 [M − Na]^−^ (calculated for [C_36_H_63_O_14_S]^−^, 751.3944).

**Ceramasteroside D** (**3**), C_41_H_72_O_16_, amorphous powder; [α]_D_^25^ −17.5° (c 0.2, MeOH); IR: *ν*_max_ = 3323, 2924, 2854, 1573, 1456, 1406, 1377, 1082, 1045, 968, 879, 636, 432 cm^−1^; ^1^H and ^13^C NMR data are listed in Table 1 and Table 2; (+)ESIMS/MS of the ion [M + Na]^+^ at *m/z* 843: 825 [(M + Na) − H_2_O]^+^, 697 [(M + Na) − C_6_H_10_O_4_]^+^, 679 [(M + Na) − C_6_H_10_O_4_ − H_2_O]^+^, 535 [(M + Na) − C_6_H_10_O_4_ − C_6_H_10_O_5_]^+^; (+)HRESIMS *m/z* 843.4715 [M + Na]^+^ (calculated for [C_41_H_72_O_16_Na]^+^, 843.4713); (−)ESIMS/MS of the ion [M − H]^−^ at *m/z* 819: 655 [(M − H) − C_6_H_10_O_4_ − H_2_O]^−^; 511 [(M − H) − C_6_H_10_O_4_ − C_6_H_10_O_5_]^−^; (−)HRESIMS *m/z* 819.4748 [M − H]^−^ (calculated for [C_41_H_71_O_16_]^−^, 819.4748).

**Ceramasteroside E** (**4**), C_39_H_66_O_15_, amorphous powder; [α]_D_^25^ −17.5° (c 0.2, MeOH); IR: *ν*_max_ = 3365, 2924, 2854, 1735, 1570, 1458, 1406, 1377, 1163, 1055, 966 cm^−1^; ^1^H and ^13^C NMR data are listed in Table 1 and Table 2; (+)ESIMS/MS of the ion [M + Na]^+^ at *m/z* 797: 779 [(M + Na) − H_2_O]^+^, 651 [(M + Na) − C_6_H_10_O_4_]^+^, 633 [(M + Na) − C_6_H_10_O_4_ − H_2_O]^+^; (+)HRESIMS *m/z* 797.4295 [M + Na]^+^ (calculated for [C_39_H_66_O_15_Na]^+^, 797.4294); (−)ESIMS/MS of the ion [M − H]^−^ at *m/z* 773: 627 [(M − H) − C_6_H_10_O_4_]^−^, 609 [(M − H) − C_6_H_10_O_4_ − H_2_O]^−^, 465 [(M − H) − C_6_H_10_O_4_ − C_6_H_10_O_5_]^−^; (−)HRESIMS *m/z* 773.4337 [M − H]^−^ (calculated for [C_39_H_65_O_15_]^−^, 773.4329).

### 3.5. Reagents

The following reagents were used to maintain and passage the cell culture: the Dulbecco’s modified Eagle medium F12 (DMEM/F12, Thermo Fisher Scientific, Waltham, MA, USA), fetal bovine serum (FBS, Thermo Fisher Scientific), horse serum (DHS, Gibco, Waltham, MA, USA), essential amino acids (Gibco, USA), penicillin-streptomycin solution (Thermo Fisher Scientific), and trypsin-ethylenediaminetetraacetic acid (trypsin-EDTA, Thermo Fisher Scientific). Hanks’ balanced salt solution (HBSS) was used to dilute the compounds (Thermo Fisher Scientific).

The cytotoxic effect of the studied compounds was assessed using the MTS reagent (3-[4,5-dimethylthiazol-2-yl]-2,5-diphenyltetrazolium bromide, Abcam, Waltham, MA, USA). DAF-FM diacetate (4-amino-5-methylamino-2′,7′-difluorofluorescein diacetate, Thermo Fisher Scientific) was used to quantify nitric oxide production. Nitrite quantification was carried out by using Griess reagent (Sigma, G4410, St. Louis, MO, USA). An MDA kit (Lipid Peroxidation (MDA) Assay Kit, MAK085, Sigma) was used to determine the production of malondialdehyde, as a marker of lipid peroxidation, under the exposure to the compounds under study.

### 3.6. Cell Lines and Culture Conditions

The murine microglial cell line SIM-A9 (CRL-3265) was obtained from the American Type Culture Collection. Cells were cultured in DMEM/F12 medium containing 10% FBS, 5% DHS, 1% penicillin/streptomycin, and essential amino acids at 37 °C in a humidified atmosphere containing 5% CO_2_. The number of passages was carefully controlled, and mycoplasma contamination was monitored on a regular basis.

### 3.7. MTS Assay

The SIM-A9 cells were plated in a 96-well plate (1 × 10^3^ cells/well) and incubated at 37 °C for 1 h in a CO_2_ incubator. Then, the standard culture medium was replaced with a medium containing the test compounds at concentrations of 0.1, 1, and 10 μM. The maximum concentration of the compounds was chosen based on the maximum permissible concentration of the solvent (EtOH), which should not exceed 0.1%. The cytotoxic activity of the compounds was measured after 72 h of exposure. Afterward, the medium in all wells was carefully removed and replaced with a fresh one supplemented with 1/10 of the volume of the MTS reagent. The plate was incubated at 37 °C in a humidified atmosphere of 5% CO_2_ for 2 h to allow color development. Optical density was measured at 490 nm on an iMark microplate reader (Bio-Rad, Hercules, CA, USA). Cell viability was estimated using absorbance in control (untreated) cells as 100% and absorbance in blank wells without cells as 0%.

### 3.8. NO Measurement

To evaluate the potential of the compounds to reduce the production of endogenously generated NO, the SIM-A9 cells were incubated for 24 h in a medium containing LPS and **1**–**6**. The cells in a standard medium without compounds served as a negative control; the cells in a medium containing only LPS served as a positive control. Staining was carried out directly in the wells of the plates, by removing the medium from them and replacing it with a 10 μM solution of DAF-FM diacetate. The cells were incubated in the dye at 37 °C for 40 min in the dark. The samples were then washed three times with PBS. The fluorescence intensity was measured on a Spark 10M plate reader (TECAN, Männedorf, Switzerland). Cell fluorescence was recorded at a reference λex = 460 nm and λem = 524 nm.

### 3.9. Griess Assay

After the incubation of the SIM-A9 cells in a medium with LPS and the exposure to test compounds for 24 h, the nitrite accumulated in the culture medium was measured as an indicator of NO production using the Griess assay method. In brief, according to the manufacturer’s protocol, 100 μL of cell culture medium was mixed with 100 μL of the Griess reagent and incubated at room temperature for 10 min. Optical density was measured at 540 nm on an iMark microplate reader (Bio-Rad).

### 3.10. MDA

MDA concentration measurements were used to assess the effect of the test compounds on the LPS-induced lipid peroxidation. The SIM-A9 cells were seeded into 24-well plates (1 × 10^5^/cm^2^) and left in the incubator for 1 h for adhesion. Then, the culture medium was replaced with a compound-containing medium and left for another 1 h. Then, LPS was added to each well to reach a concentration of 1 μg/mL. The MDA analysis was carried out after 24 h. For analysis, the cells from each well were passaged using trypsin/EDTA solution and transferred into separate tubes. The cells were then subjected to ultrasonic homogenization. All further manipulations were carried out in accordance with the manufacturer’s instructions. Optical density was measured on an iMark microplate reader (Bio-Rad) at a wavelength of 532 nm. The results were presented as relative to the negative control group without LPS added.

### 3.11. Statistical Analysis

All the assays were performed in at least triplicate. All data were tested for normality of distribution using the Shapiro–Wilk test. The data obtained from the in vitro study were compared using the Student’s *t*-test. The results were expressed as the mean ± standard error of the mean (SEM), with *p* < 0.05 considered statistically significant. All statistical calculations and plotting were performed using the GraphPad Prism 4.00 software (GraphPad Software, San Diego, CA, USA).

## 4. Conclusions

Six polyhydroxysteroid glycosides, including four new compounds, ceramasterosides A, B, D, and E (**1**–**4**), have been isolated from orange cookie stars, *C. patagonicus*. Five polyhydroxysteroid glycosides, ceramasterosides C_1_-C_5_, containing steroid nuclei of varying oxidation states (penta-, hexa-, or heptahydroxy), ∆^22^-cholestane or ∆^22^-ergostane side chains, and two monosaccharide residues (galactofuranose, xylopyranose or its methylated derivatives at C-2 or C-4), were isolated from *C. patagonicus* previously [20]. In the present study, we have discovered and described steroid glycosides with a number of specific structural features. In particular, the 3-*O*-Me-4-*O*-SO3--glucopyranose residue (ceramasteroside B (**2**)) has been found for the first time in starfish-derived steroid glycosides, while the 3-*O*-Me-4-*O*-SO3-xylopyranose residue (ceramasteroside A (**1**)) is very rare and previously was found only in scoparioside C from *A. scoparius* [25] and antarcticoside J from sea stars of the family Echinasteridae [29]. In addition, the previously isolated polyhydroxysteroid glycosides did not contain a saturated stigmastane side chain, as in ceramasterosides A, B, and D (**1**–**3**), sodium sulfate groups, as in ceramasterosides A (**1**) and B (**2**), and an arabinofuranose residue, as in attenuatoside B-I (**6**). It is also worth noting that the ∆^22^-24-methyl-27-*nor*-cholestane side chain found in ceramasteroside E (**4**) rarely occurs in starfish-derived polyhydroxysteroid glycosides [1,2,3,4,5,6,7,8]. Furthermore, all previously isolated polyhydroxysteroid glycosides from *C. patagonicus* (ceramasterosides C_1_–C_5_) are actually biglycosides, while ceramasterosides A (**1**) and B (**2**) have only one monosaccharide residue. Based on the above findings, we have concluded that the structural diversity of polyhydroxysteroid glycosides in the sea star *C. patagonicus* is significantly greater than previously described.

In our present study, we have demonstrated the antioxidant activity of starfish-derived steroid glycosides on murine microglial cells. Thus, all the isolated compounds at a non-toxic concentration of 1 μM significantly reduced the increased NO production induced by LPS in microglial cells, compared to LPS-treated cells (positive control). In addition, ceramasteroside E (**4**) at the same concentration significantly reduced the level of nitrites and malonic dialdehyde, an important marker of lipid peroxidation. Thus, we have shown, for the first time, that starfish-derived polyhydroxysteroid glycosides not only promote neurite differentiation and protect them from oxygen-glucose deprivation but also exhibit noticeable antioxidant activity toward microglial cells, which plays an important role in the development of many neurodegenerative diseases. This makes this class of marine-derived metabolites even more promising for addressing a wide range of issues in pharmacology and medicine.

## Figures and Tables

**Table 1:** ^1^H (700 MHz) and ^13^C (175 MHz) NMR data of aglycon parts of **1**–**4** (30 °C, CD_3_OD, *J* in Hz) ^a^.

Position	1,2		3		4	
	* δ *_ H _	* δ *_ C _	* δ *_ H _	* δ *_ C _	* δ *_ H _	* δ *_ C _
1	1.70 m 0.96 m	39.4	1.70 m 0.97 m	39.6	1.70 m 0.99 m	39.7
2	1.72 m 1.45 m	31.4	1.82 m 1.55 m	26.2	1.82 m 1.55 m	26.5
3	3.47 m	72.2	3.42 m	73.7	3.42 m	73.7
4	2.19 brd (12.3) 1.20 m	32.4	4.26 brs	69.1	4.25 brs	69.1
5	1.04 m	53.8	0.94 m	57.2	0.94 dd (11.2, 1.8)	57.2
6	3.71 td (11.0, 4.4)	67.7	4.18 td (11.2, 3.5)	64.8	4.15 m	64.8
7	2.39 dd (12.5, 4.4) 1.33 m	49.6	2.46 dd (12.5, 3.5) 1.35 dd (12.5, 11.2)	50.1	2.43 dd (12.5, 4.4) 1.30 m	49.6
8		77.2		77.2		77.4
9	0.83 m	57.4	0.82 dd (12.9, 2.9)	58.4	0.83 dd (12.7, 3.2)	58.4
10		38.0		38.2		38.2
11	1.77 m 1.47 m	19.4	1.76 m 1.41 m	18.9	1.78 m 1.44 m	19.2
12	1.94 m 1.13 m	43.5	1.94 brd (12.5) 1.11 m	43.5	1.97 dt (12.7, 3.0) 1.17 m	43.3
13		44.5		44.5		44.2
14	1.02 d (5.7)	61.1	1.01 d (5.6)	61.1	1.01 d (5.8)	62.9
15	4.36 dd (6.8, 5.7)	71.2	4.37 dd (6.8, 5.6)	71.2	4.39 td (7.2, 2.0)	71.2
16	4.22 m	72.8	4.22 t (6.8)	72.8	2.21 m 1.35 m	43.6
17	0.96 m	62.9	0.95 m	63.0	1.00 m	57.6
18	1.24 s	17.9	1.23 s	17.9	1.27 s	16.7
19	0.98 s	14.0	1.15 s	17.0	1.15 s	17.0
20	1.89 m	31.5	1.91 m	31.4	2.12 m	41.1
21	0.94 d (6.6)	18.5	0.95 d (6.6)	18.6	0.99 d (6.6)	21.2
22	1.72 m 1.08 m	34.6	1.70 m 1.08 m	34.7	5.21 dd (15.1, 8.6)	137.2
23	1.43 m 1.15 m	28.8	1.41 m 1.18 m	28.6	5.15 dd (15.1, 7.8)	134.4
24	1.25 m	42.3	1.26 m	42.2	2.23 m	34.9
25	1.74 m	30.6	1.76 m	30.5	1.59 m 1.44 m	38.0
26	0.87 d (6.7)	20.0	0.87 d (6.8)	20.1	3.70 m 3.39 m	67.2
27	0.84 d (6.7)	19.1	0.85 d (6.8)	19.0		
28	1.71 m 1.46 m	31.7	1.62 m 1.42 m	31.6	0.96 d (6.7)	21.7
29	3.83 m/3.92 m ^b^ 3.54 m	70.0	3.73 m 3.43 m	68.0		

^a^ Assignments from 700 MHz ^1^H-^1^H COSY, HSQC, HMBC (8 Hz), and ROESY (270 msec) data. ^b^ Obtained for H-29 of **2**.

**Table 2:** ^1^H (700 MHz) and ^13^C (175 MHz) NMR data of carbohydrate parts of **1**–**4** (30 °C, CD_3_OD, *J* in Hz) ^a^.

**Position**	**1**		**2**		**3,4**	
	* δ *_ H _	* δ *_ C _	* δ *_ H _	* δ *_ C _	* δ *_ H _	* δ *_ C _
	**3-OMe-4-OSO_3_-Xyl_p_**		**3-OMe-4-OSO_3_-Glc_p_**		**Gal_f_**	
1′	4.23 d (7.3)	104.8	4.27 d (7.5)	104.4	5.00 brd (1.5)	108.0
2′	3.25 dd (9.7, 7.3)	74.3	3.24 m	74.7	4.05 dd (4.1, 1.5)	91.8
3′	3.18 t (8.7)	85.1	3.22 m	86.3	4.15 dd (7.7, 4.1)	77.6
4′	4.25 m	77.2	4.18 dd (10.0, 8.6)	77.3	3.85 dd (7.7, 3.6)	83.0
5′	4.24 m 3.34 m	64.8	3.35 m	76.7	3.70 m	72.2
6′			3.89 dd (12.8, 2.6) 3.81 dd (12.8, 5.0)	62.6	3.62 d (6.2)	64.8
3′-OMe	3.61 s	60.7	3.62 s	61.1		
					**2-OMe-Xyl_p_**	
1″					4.45 d (7.7)	104.7
2″					2.85 dd (9.2, 7.7)	84.8
3″					3.31 t (9.2)	77.4
4″					3.48 m	71.2
5″					3.83 dd (11.2, 5.6) 3.16 dd (11.2, 10.6)	67.0
2″-OMe					3.58 s	61.2

^a^ Assignments from 700 MHz ^1^H-^1^H COSY, HSQC, HMBC (8 Hz), and ROESY (270 msec) data.

## Data Availability

The data presented in this study are available on request from the corresponding authors.

## Supplementary Materials

The following supporting information can be downloaded at: https://www.mdpi.com/article/10.3390/md22110508/s1, Figure S1: (+)-HRESIMS spectrum of ceramasteroside A (**1**); Figure S2: (−)-HRESIMS spectrum of ceramasteroside A (**1**); Figure S3: (−)-ESIMS/MS spectrum of ceramasteroside A (**1**); Figure S4: IR spectrum of ceramasteroside A (**1**) in thin layer; Figure S5: ^1^H NMR spectrum of ceramasteroside A (**1**) in CD_3_OD; Figure S6: ^13^C NMR spectrum of ceramasteroside A (**1**) in CD_3_OD; Figure S7: ^1^H-^1^H COSY spectrum of ceramasteroside A (**1**) in CD_3_OD; Figure S8: HSQC spectrum of ceramasteroside A (**1**) in CD_3_OD; Figure S9: HMBC spectrum of ceramasteroside A (**1**) in CD_3_OD; Figure S10: ROESY spectrum of ceramasteroside A (**1**) in CD_3_OD; Figure S11: (+)-HRESIMS spectrum of ceramasteroside B (**2**); Figure S12: (−)-HRESIMS spectrum of ceramasteroside B (**2**); Figure S13: (−)-ESIMS/MS spectrum of ceramasteroside B (**2**); Figure S14: IR spectrum of ceramasteroside B (**2**) in thin layer; Figure S15: ^1^H NMR spectrum of ceramasteroside B (**2**) in CD_3_OD; Figure S16: ^13^C NMR spectrum of ceramasteroside B (**2**) in CD_3_OD; Figure S17: ^1^H-^1^H COSY spectrum of ceramasteroside B (**2**) in CD_3_OD; Figure S18: HSQC spectrum of ceramasteroside B (**2**) in CD_3_OD; Figure S19: HMBC spectrum of ceramasteroside B (**2**) in CD_3_OD; Figure S20: ROESY spectrum of ceramasteroside B (**2**) in CD_3_OD; Figure S21: (+)-HRESIMS spectrum of ceramasteroside D (**3**); Figure S22: (−)-HRESIMS spectrum of ceramasteroside D (**3**); Figure S23: (−)-ESIMS/MS spectrum of ceramasteroside D (**3**); Figure S24: IR spectrum of ceramasteroside D (**3**) in thin layer; Figure S25: ^1^H NMR spectrum of ceramasteroside D (**3**) in CD_3_OD; Figure S26: ^13^C NMR spectrum of ceramasteroside D (**3**) in CD_3_OD; Figure S27: ^1^H-^1^H COSY spectrum of ceramasteroside D (**3**) in CD_3_OD; Figure S28: HSQC spectrum of ceramasteroside D (**3**) in CD_3_OD; Figure S29: HMBC spectrum of ceramasteroside D (**3**) in CD_3_OD; Figure S30: ROESY spectrum of ceramasteroside D (**3**) in CD_3_OD; Figure S31: (+)-HRESIMS spectrum of ceramasteroside E (**4**); Figure S32: (−)-HRESIMS spectrum of ceramasteroside E (**4**); Figure S33: (−)-ESIMS/MS spectrum of ceramasteroside E (**4**); Figure S34: IR spectrum of ceramasteroside E (**4**) in thin layer; Figure S35: ^1^H NMR spectrum of ceramasteroside E (**4**) in CD_3_OD; Figure S36: ^13^C NMR spectrum of ceramasteroside E (**4**) in CD_3_OD; Figure S37: ^1^H-^1^H COSY spectrum of ceramasteroside E (**4**) in CD_3_OD; Figure S38: HSQC spectrum of ceramasteroside E (**4**) in CD_3_OD; Figure S39: HMBC spectrum of ceramasteroside E (**4**) in CD_3_OD; Figure S40: ROESY spectrum of ceramasteroside E (**4**) in CD_3_OD.
