# Supplementary material for: New Polyhydroxysteroid Glycosides with Antioxidant Activity from the Far Eastern Sea Star Ceramaster patagonicus
Source: Mar Drugs. 2024 Nov 10;22(11):508. doi: 10.3390/md22110508 (PMC11595467; doi:10.3390/md22110508)

New Polyhydroxysteroid Glycosides with Antioxidant Activity from the Far Eastern Sea Star *Ceramaster patagonicus*

Timofey V. Malyarenko[1,2*], Viktor M. Zakharenko[1,2], Alla A. Kicha[1], Arina I. Ponomarenko[3], Igor V. Manzhulo[3], Anatoly I. Kalinovsky[1], Roman S. Popov[1], Pavel S. Dmitrenok[1] and Natalia V. Ivanchina[1*]

[1] G.B. Elyakov Pacific Institute of Bioorganic Chemistry, Far Eastern Branch, Russian Academy of Sciences, Pr. 100-let Vladivostoku 159, 690022, Vladivostok, Russia; rarf247@gmail.com (V.M.Z.); kicha@piboc.dvo.ru (A.A.K.); kaaniw@piboc.dvo.ru (A.I.K.); prs_90@mail.ru (R.S.P.); paveldmt@piboc.dvo.ru (P.S.D.)

[2] Department of Chemistry and Materials, Institute of High Technology and Advanced Materials, Far Eastern Federal University, Russky Island, Ajax Bay, 10, 690922, Vladivostok, Russia

[3] A.V. Zhirmunsky National Scientific Center of Marine Biology, Far Eastern Branch, Russian Academy of Sciences, ul. Palchevskogo 17, 690041, Vladivostok, Russia; arina.ponomarenko.93@mail.ru (A.I.P.); i-manzhulo@bk.ru (I.V.M.)

* Correspondence: malyarenko-tv@mail.ru (T.V.M.); ivanchina@piboc.dvo.ru (N.V.I.). Tel.: +7-423-2312-360; Fax: +7-423-2314-050

List

Figure S1. (+)-HRESIMS spectrum of ceramasteroside A (**1**).

Figure S2. (–)-HRESIMS spectrum of ceramasteroside A (**1**).

Figure S3. (–)-ESIMS/MS spectrum of ceramasteroside A (**1**).

Figure S4. IR spectrum of ceramasteroside A (**1**) in thin layer.

Figure S5. ^{1}H NMR spectrum of ceramasteroside A (**1**) in CD_3OD.

Figure S6. ^{13}C NMR spectrum of ceramasteroside A (**1**) in CD_3OD.

Figure S7. ^{1}H-^{1}H COSY spectrum of ceramasteroside A (**1**) in CD_3OD.

Figure S8. HSQC spectrum of ceramasteroside A (**1**) in CD_3OD.

Figure S9. HMBC spectrum of ceramasteroside A (**1**) in CD_3OD.

Figure S10. ROESY spectrum of ceramasteroside A (**1**) in CD_3OD.

Figure S11. (+)-HRESIMS spectrum of ceramasteroside B (**2**).

Figure S12. (–)-HRESIMS spectrum of ceramasteroside B (**2**).

Figure S13. (–)-ESIMS/MS spectrum of ceramasteroside B (**2**).

Figure S14. IR spectrum of ceramasteroside B (**2**) in thin layer.

Figure S15. ^{1}H NMR spectrum of ceramasteroside B (**2**) in CD_3OD.

Figure S16. ^{13}C NMR spectrum of ceramasteroside B (**2**) in CD_3OD.

Figure S17. ^{1}H-^{1}H COSY spectrum of ceramasteroside B (**2**) in CD_3OD.

Figure S18. HSQC spectrum of ceramasteroside B (**2**) in CD_3OD.

Figure S19. HMBC spectrum of ceramasteroside B (**2**) in CD_3OD.

Figure S20. ROESY spectrum of ceramasteroside B (**2**) in CD_3OD.

Figure S21. (+)-HRESIMS spectrum ceramasteroside D (**3**).

Figure S22. (–)-HRESIMS spectrum of ceramasteroside D (**3**).

Figure S23. (–)-ESIMS/MS spectrum of ceramasteroside D (**3**).

Figure S24. IR spectrum of ceramasteroside D (**3**) in thin layer.

Figure S25. ^{1}H NMR spectrum of ceramasteroside D (**3**) in CD_3OD.

Figure S26. ^{13}C NMR spectrum of ceramasteroside D (**3**) in CD_3OD.

Figure S27. ^{1}H-^{1}H COSY spectrum of ceramasteroside D (**3**) in CD_3OD.

Figure S28. HSQC spectrum of ceramasteroside D (**3**) in CD_3OD.

Figure S29. HMBC spectrum of ceramasteroside D (**3**) in CD_3OD.

Figure S30. ROESY spectrum of ceramasteroside D (**3**) in CD_3OD.

Figure S31. (+)-HRESIMS spectrum of ceramasteroside E (**4**).

Figure S32. (–)-HRESIMS spectrum of ceramasteroside E (**4**).

Figure S33. (–)-ESIMS/MS spectrum of ceramasteroside E (**4**).

Figure S34. IR spectrum of ceramasteroside E (**4**) in thin layer.

Figure S35. ^{1}H NMR spectrum of ceramasteroside E (**4**) in CD_3OD.

Figure S36. ^{13}C NMR spectrum of ceramasteroside E (**4**) in CD_3OD.

Figure S37. ^{1}H-^{1}H COSY spectrum of ceramasteroside E (**4**) in CD_3OD.

Figure S38. HSQC spectrum of ceramasteroside E (**4**) in CD_3OD.

Figure S39. HMBC spectrum of ceramasteroside E (**4**) in CD_3OD.

Figure S40. ROESY spectrum of ceramasteroside E (**4**) in CD_3OD.

Figure S1. (+)-HRESIMS spectrum of ceramasteroside A (**1**).

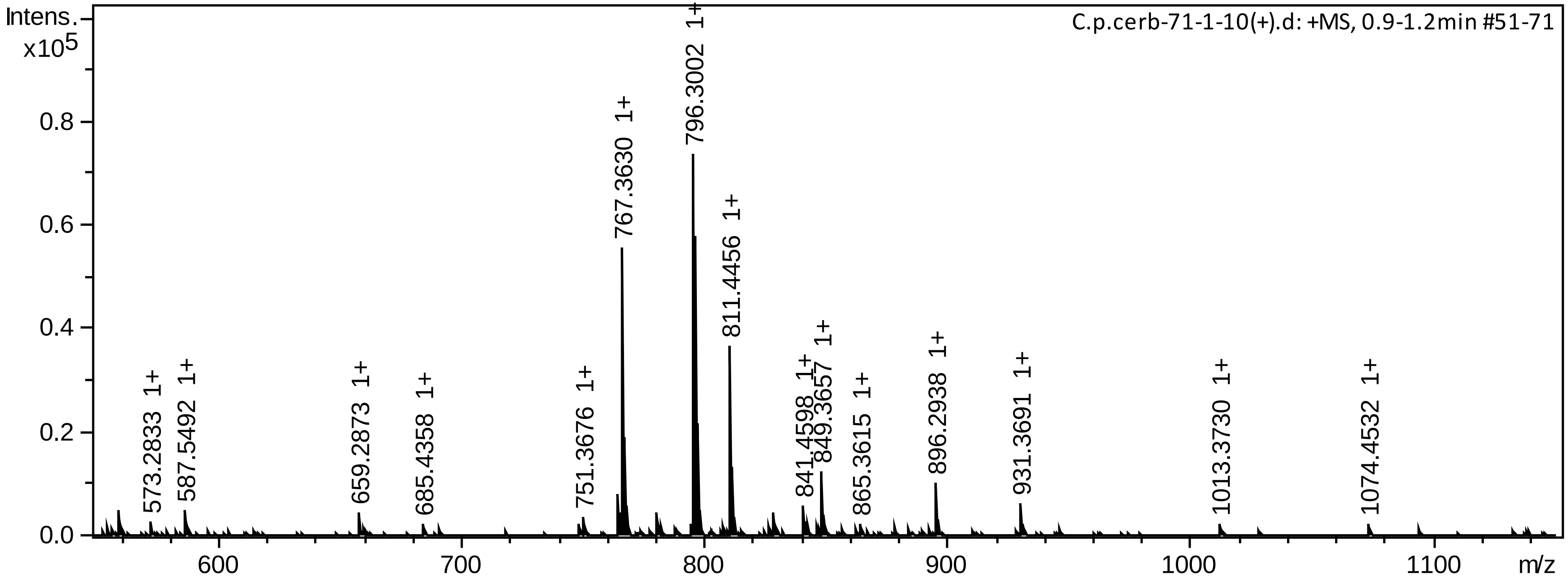

Figure S2. (–)-HRESIMS spectrum of ceramasteroside A (**1**).

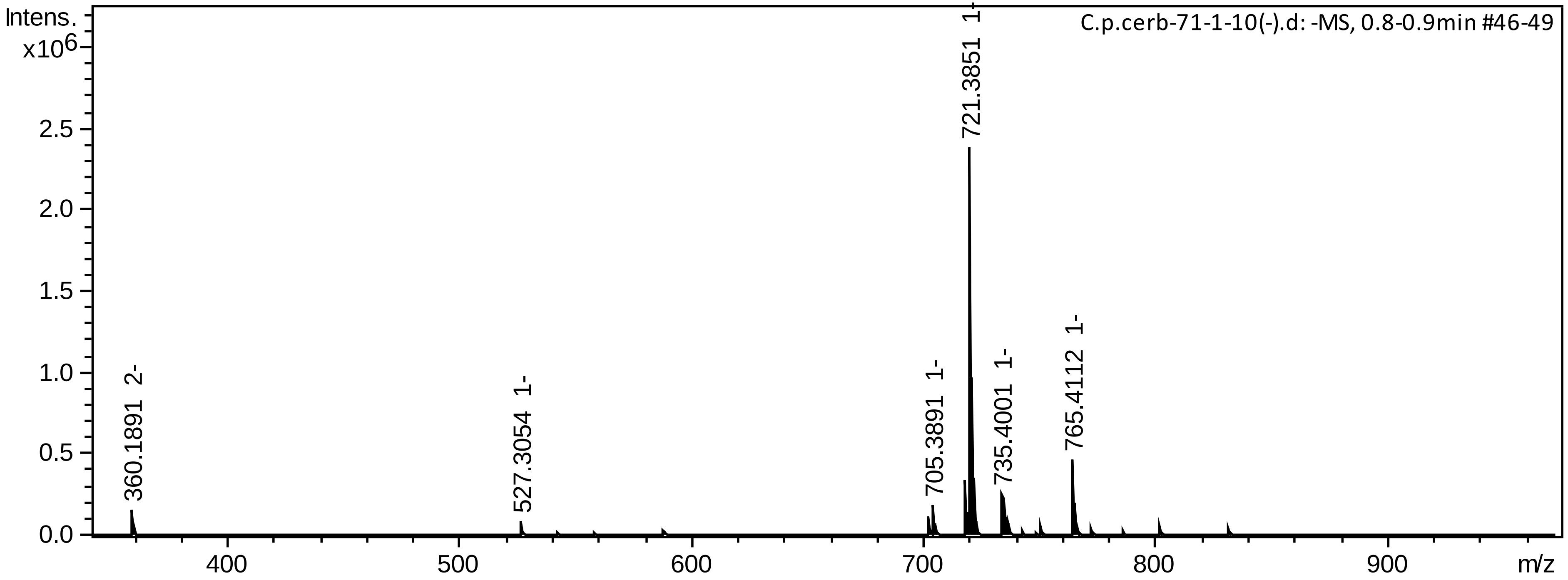

Figure S3. (–)-ESIMS/MS spectrum of ceramasteroside A (**1**).

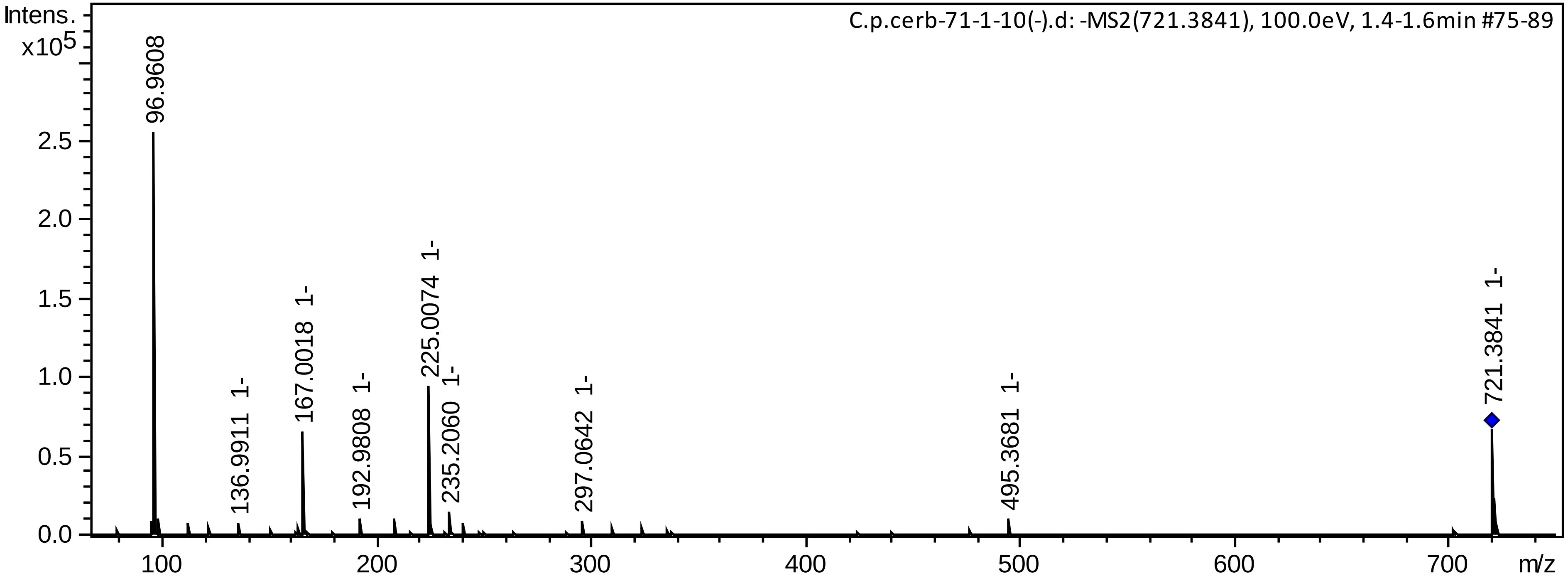

Figure S4. IR spectrum of ceramasteroside A (**1**) in thin layer.

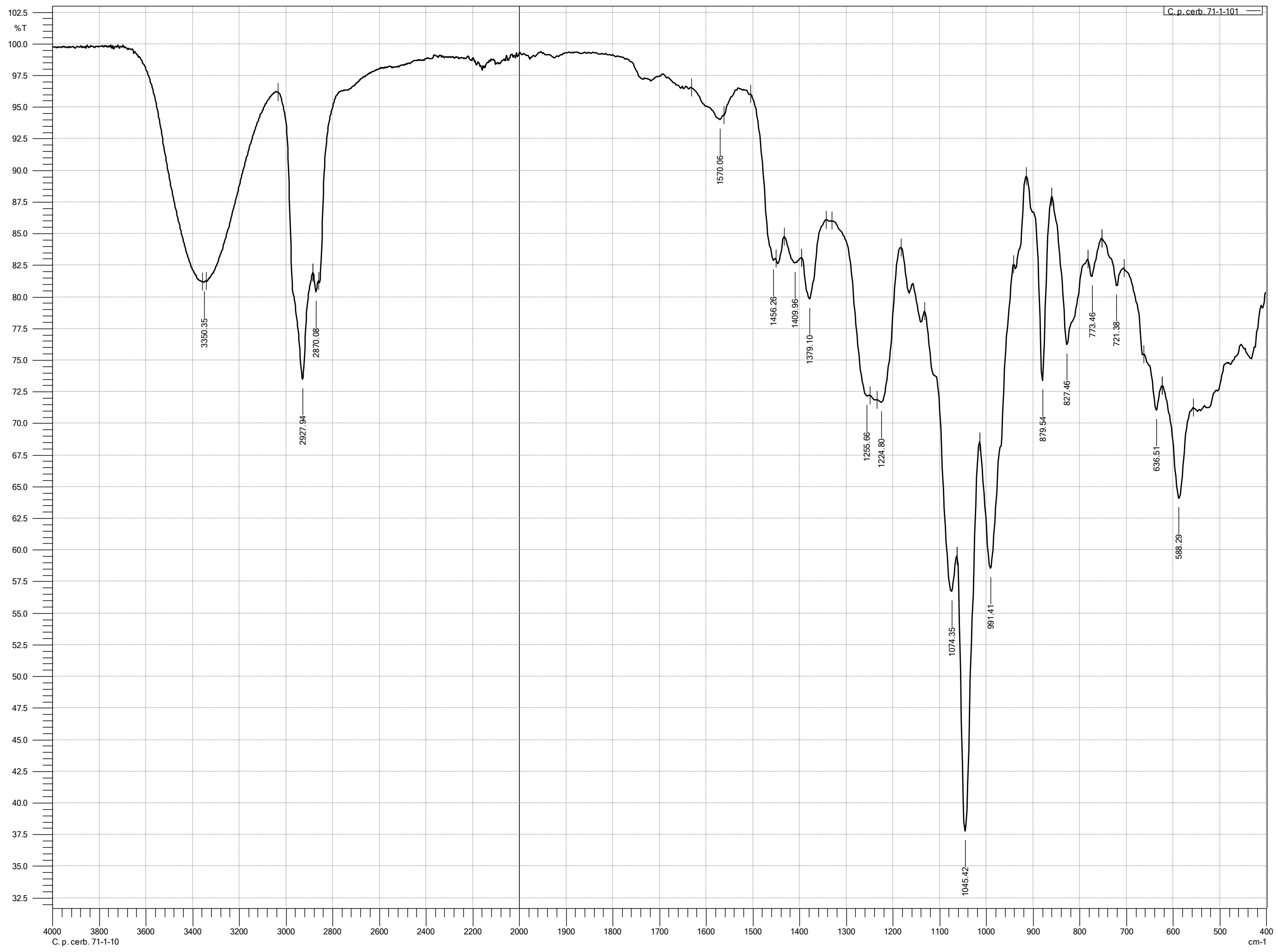

Figure S5. ^{1}H NMR spectrum of ceramasteroside A (**1**) in CD_3OD.

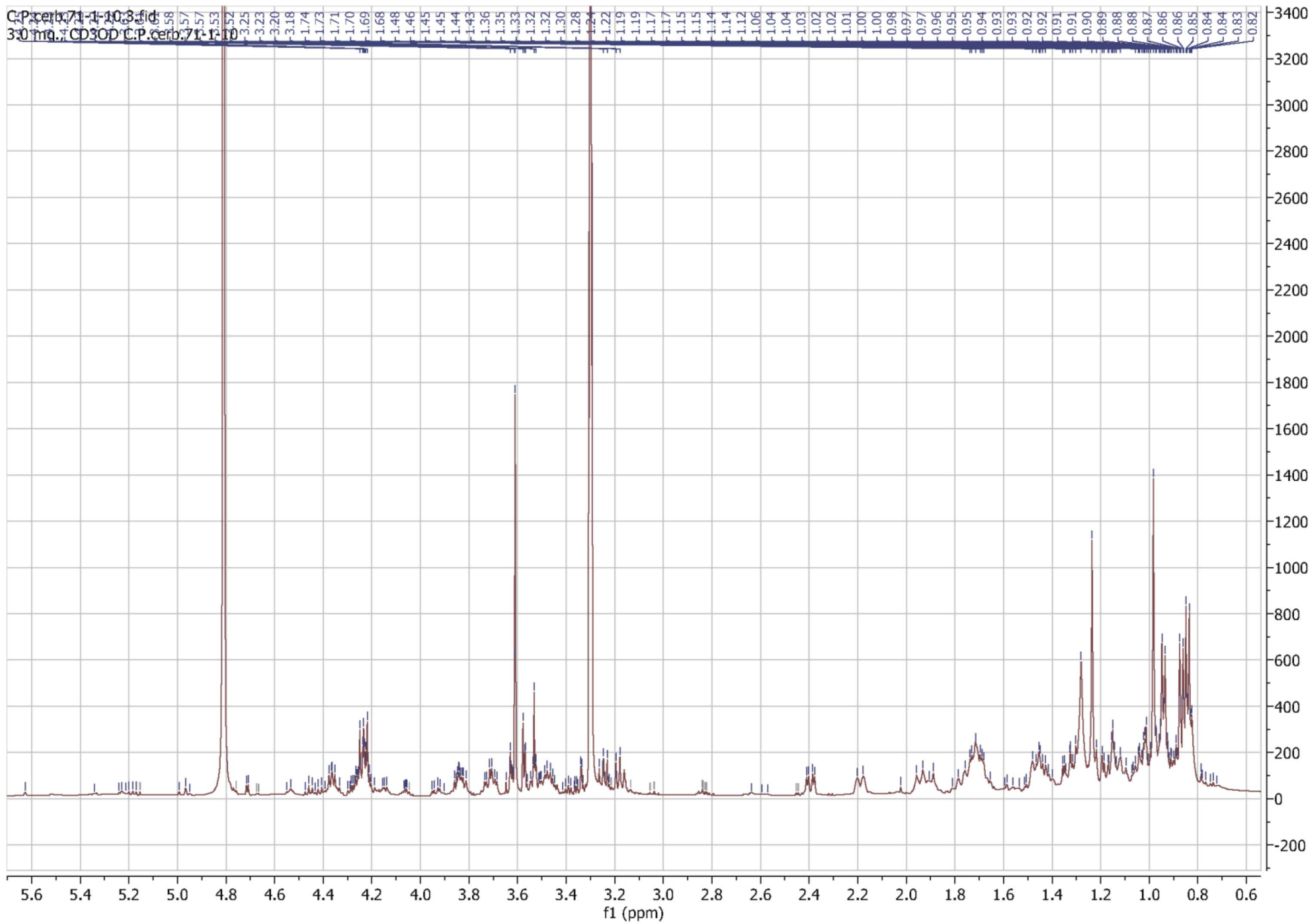

Figure S6. ^{13}C NMR spectrum of ceramasteroside A (**1**) in CD_3OD.

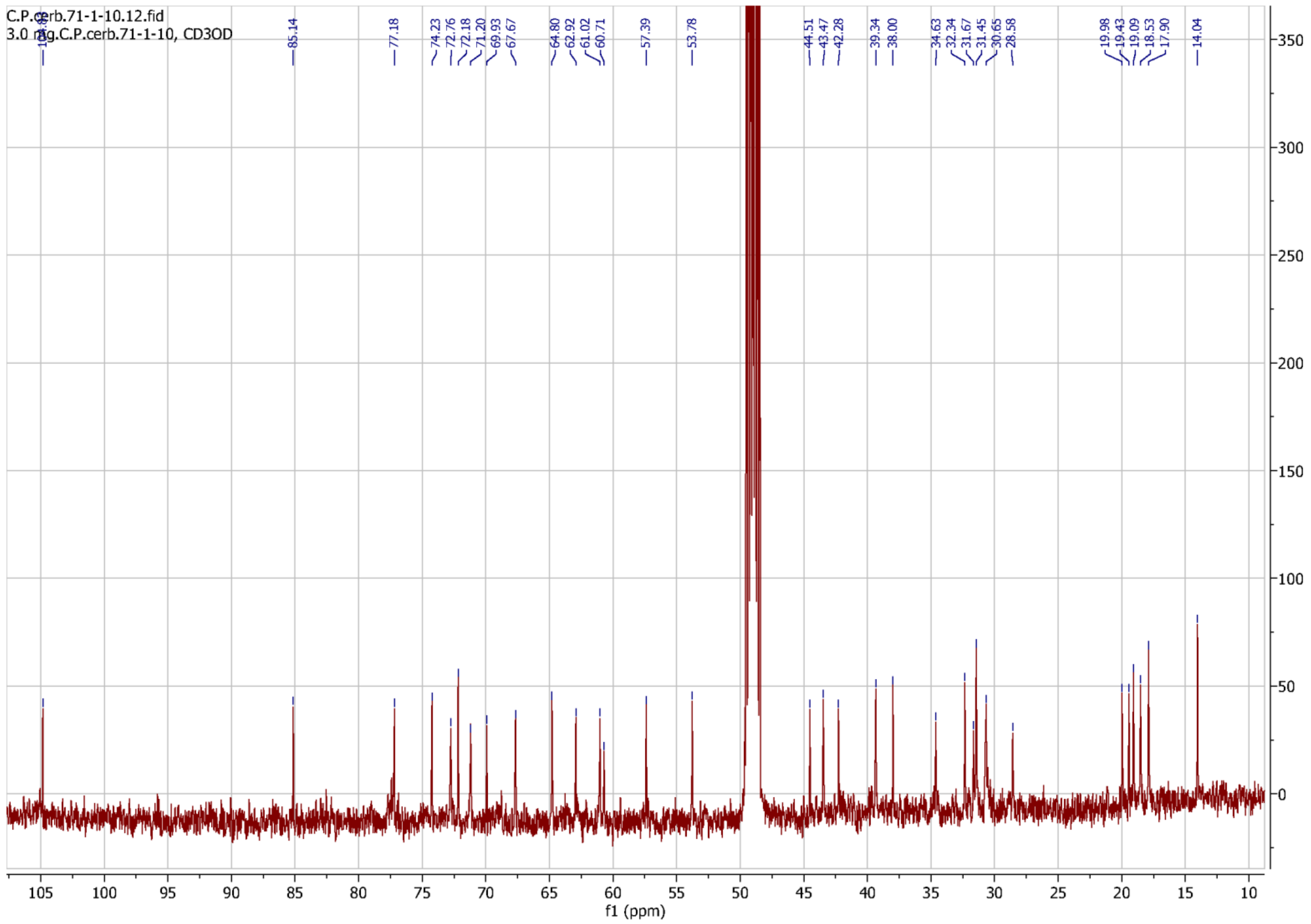

Figure S7. ^{1}H-^{1}H COSY spectrum of ceramasteroside A (**1**) in CD_3OD.

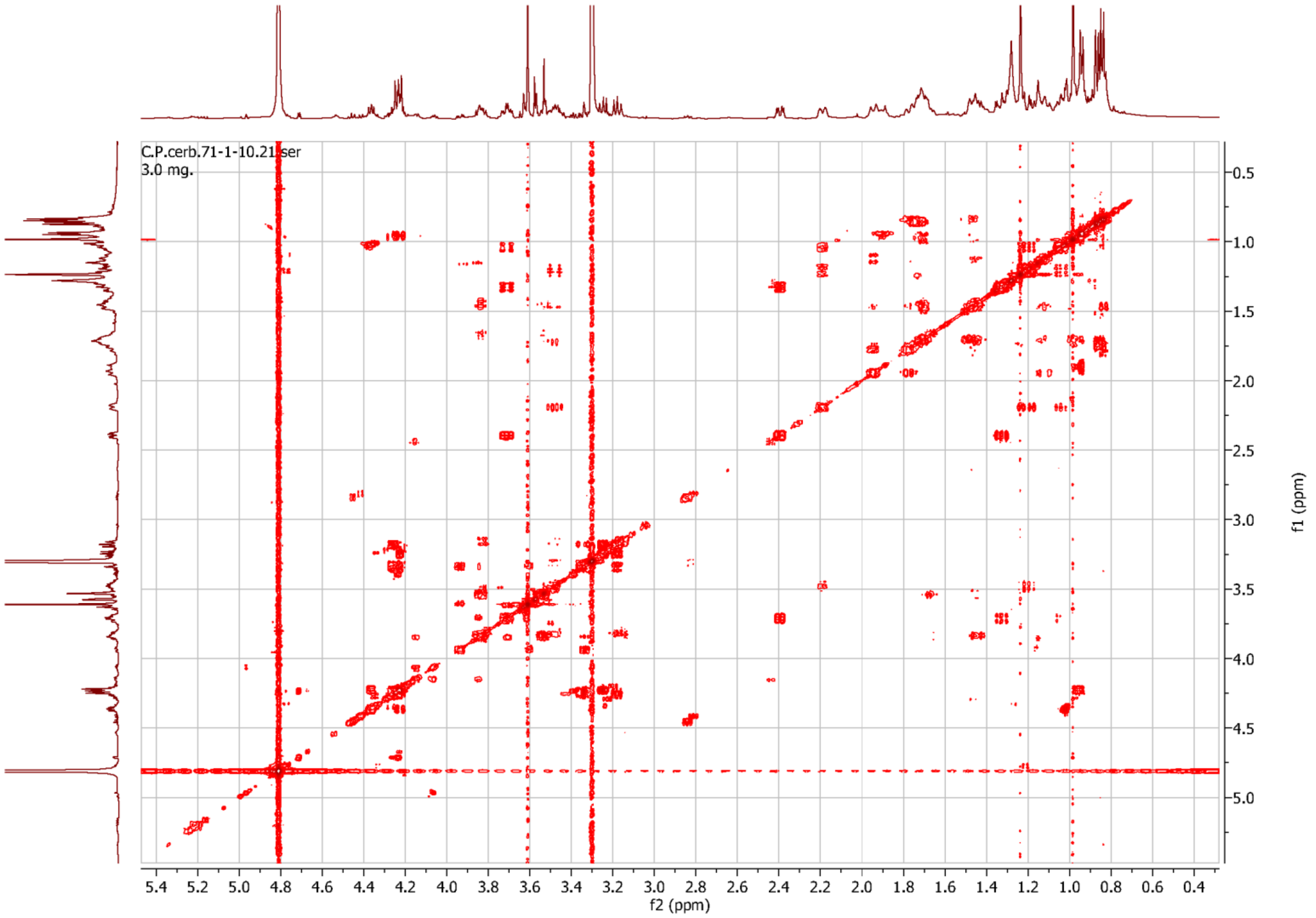

Figure S8. HSQC spectrum of ceramasteroside A (**1**) in CD_3OD.

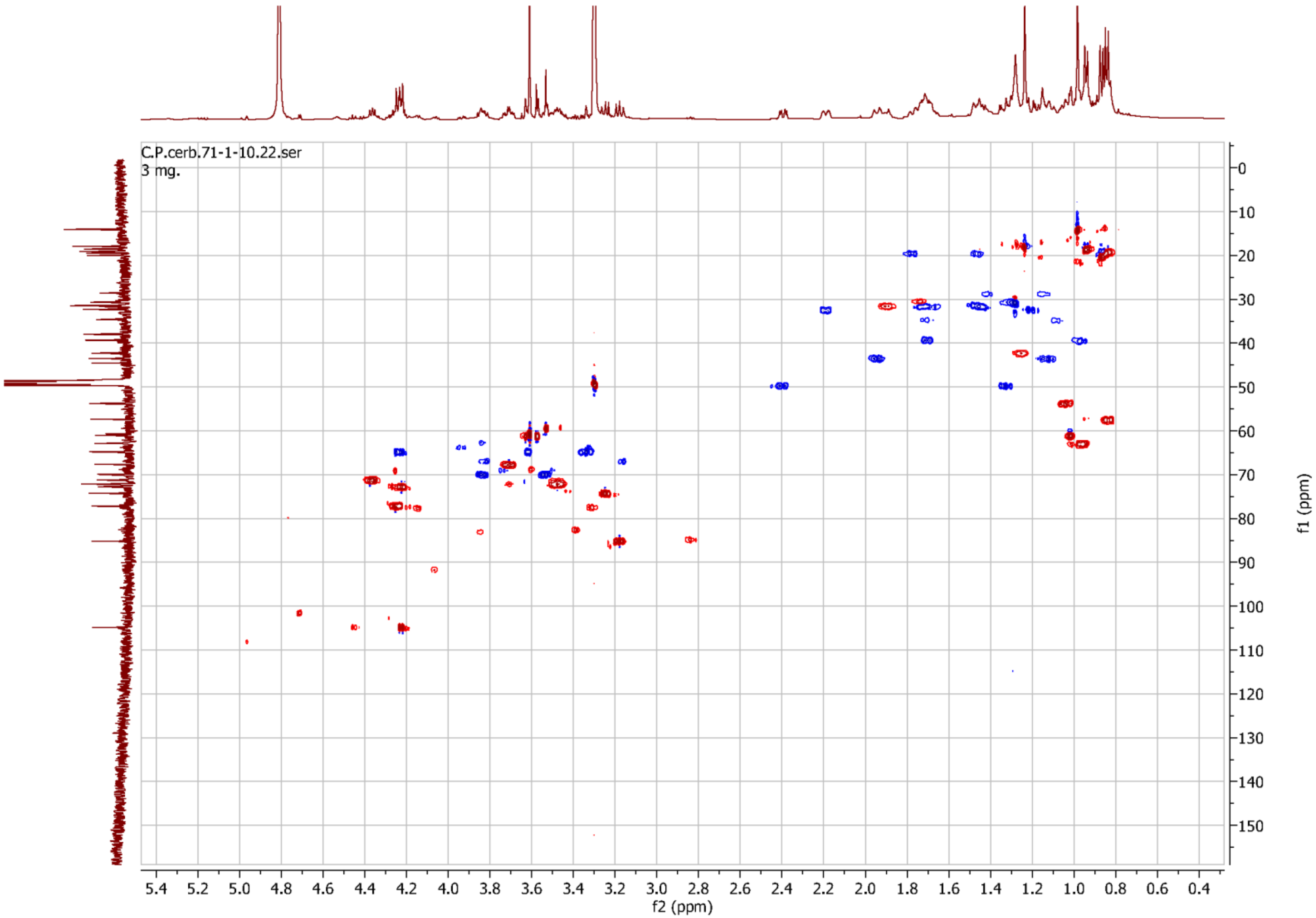

Figure S9. HMBC spectrum of ceramasteroside A (**1**) in CD3OD.

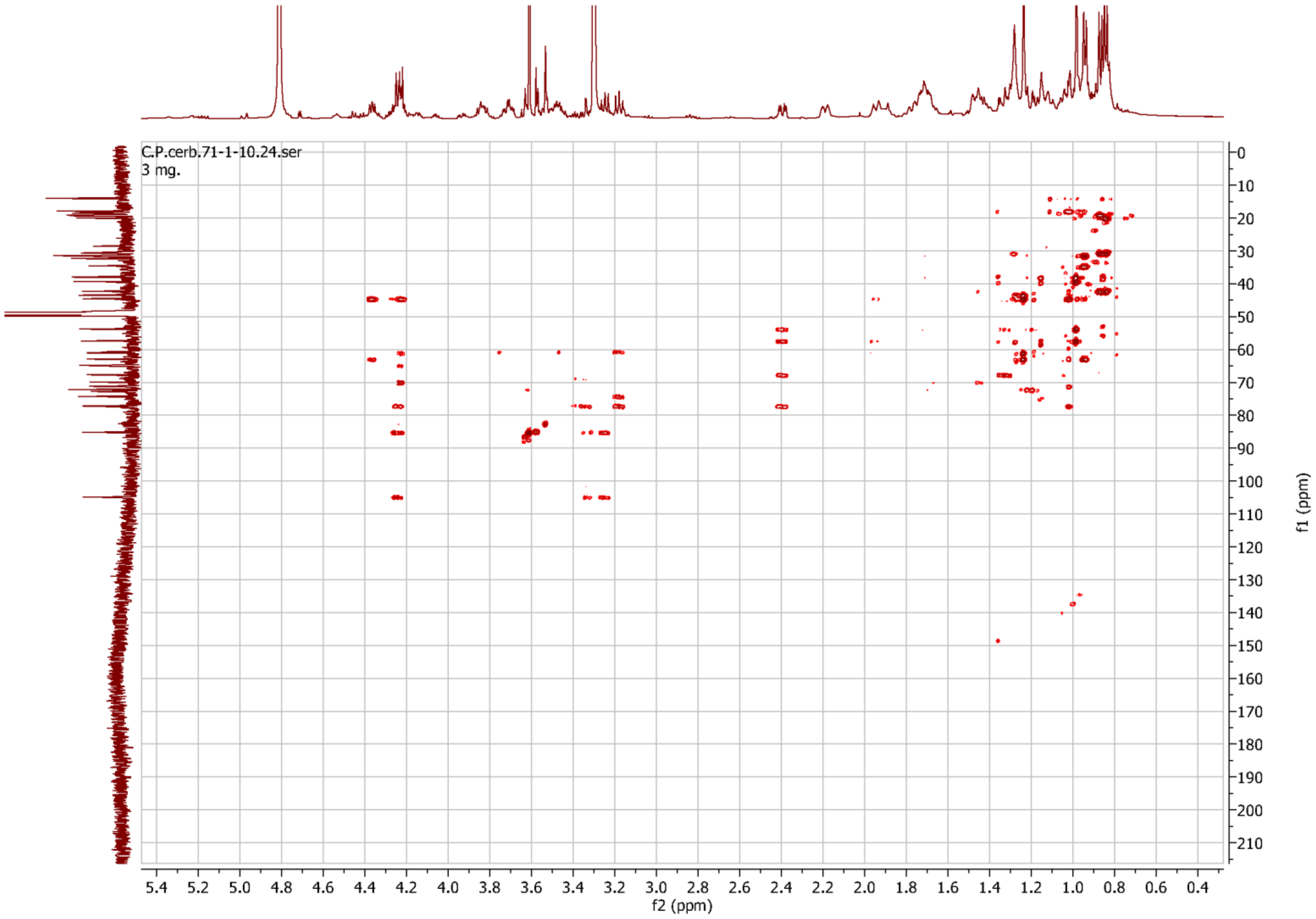

Figure S10. ROESY spectrum of ceramasteroside A (**1**) in CD_3OD.

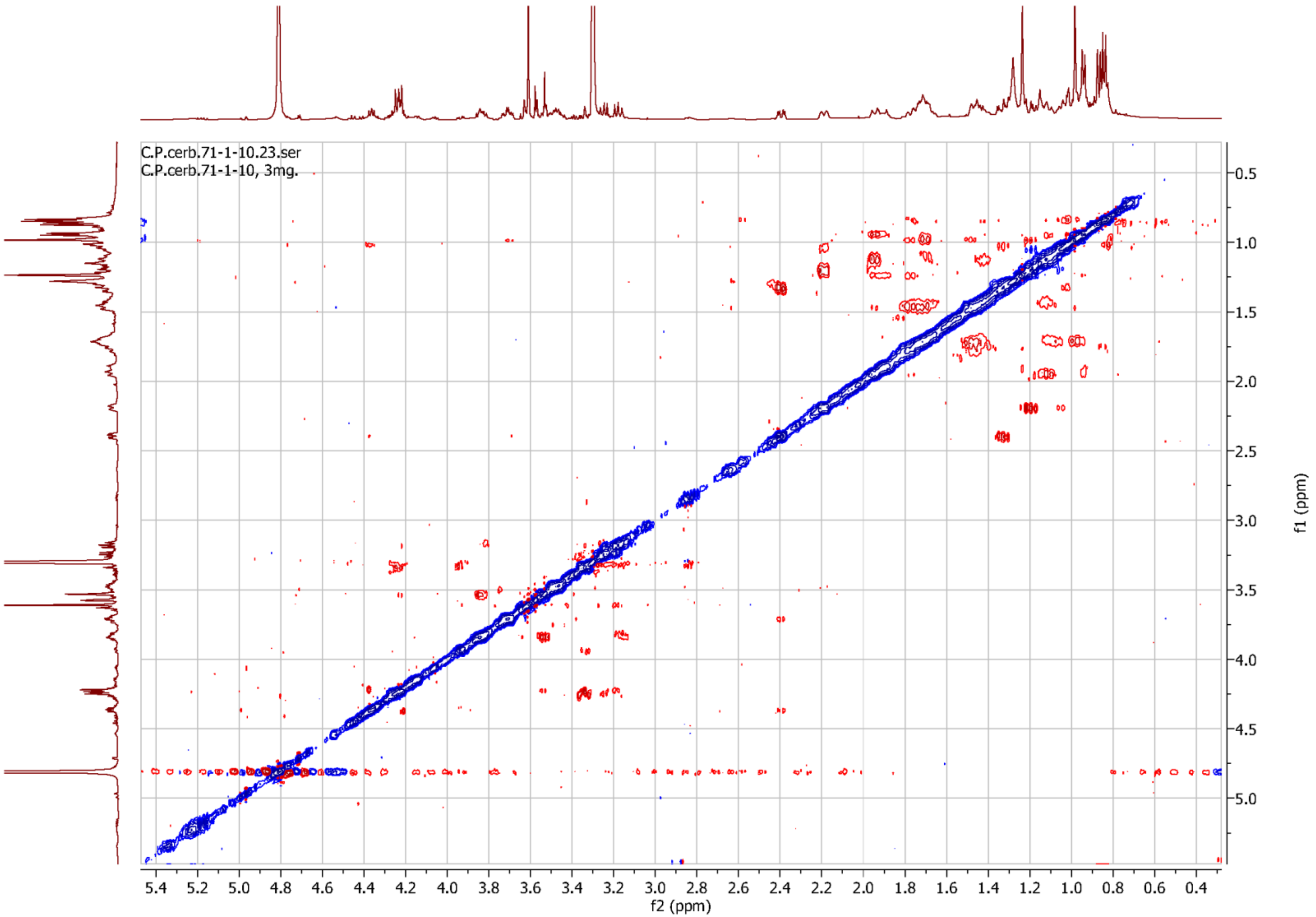

Figure S11. (+)-HRESIMS spectrum of ceramasteroside B (**2**).

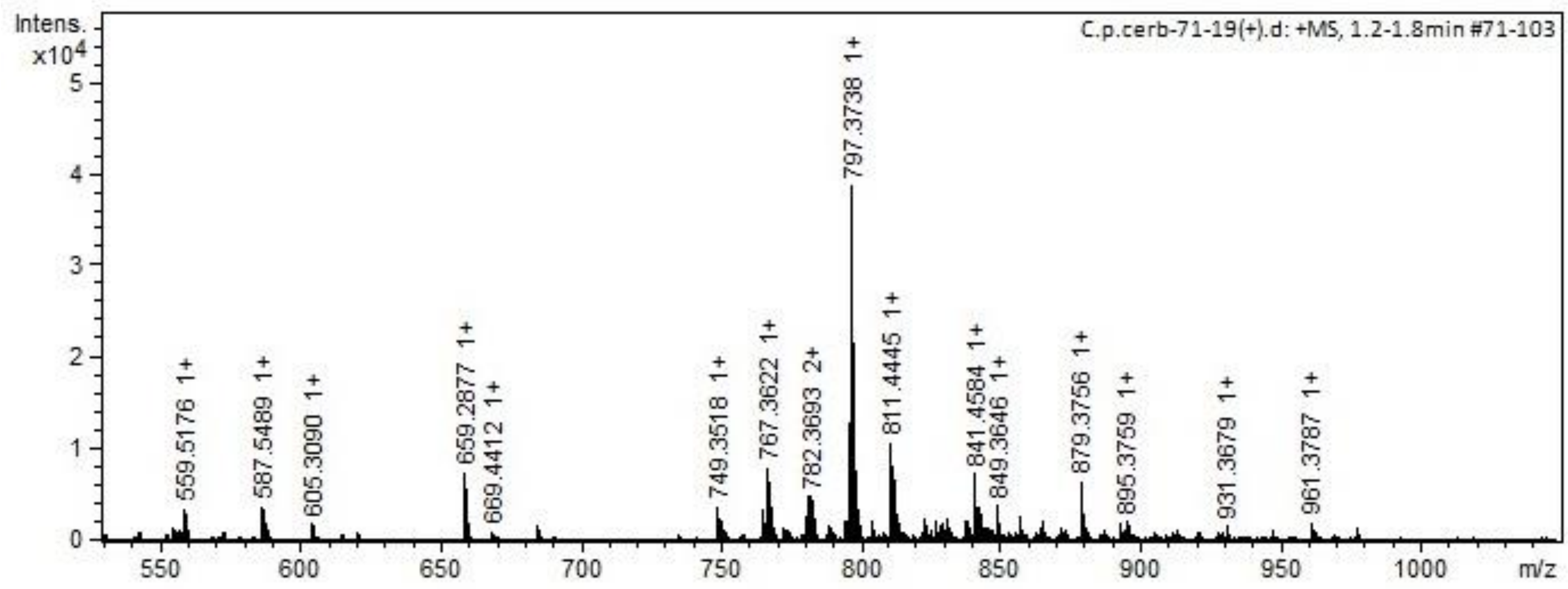

Figure S12. (–)-HRESIMS spectrum of ceramasteroside B (**2**).

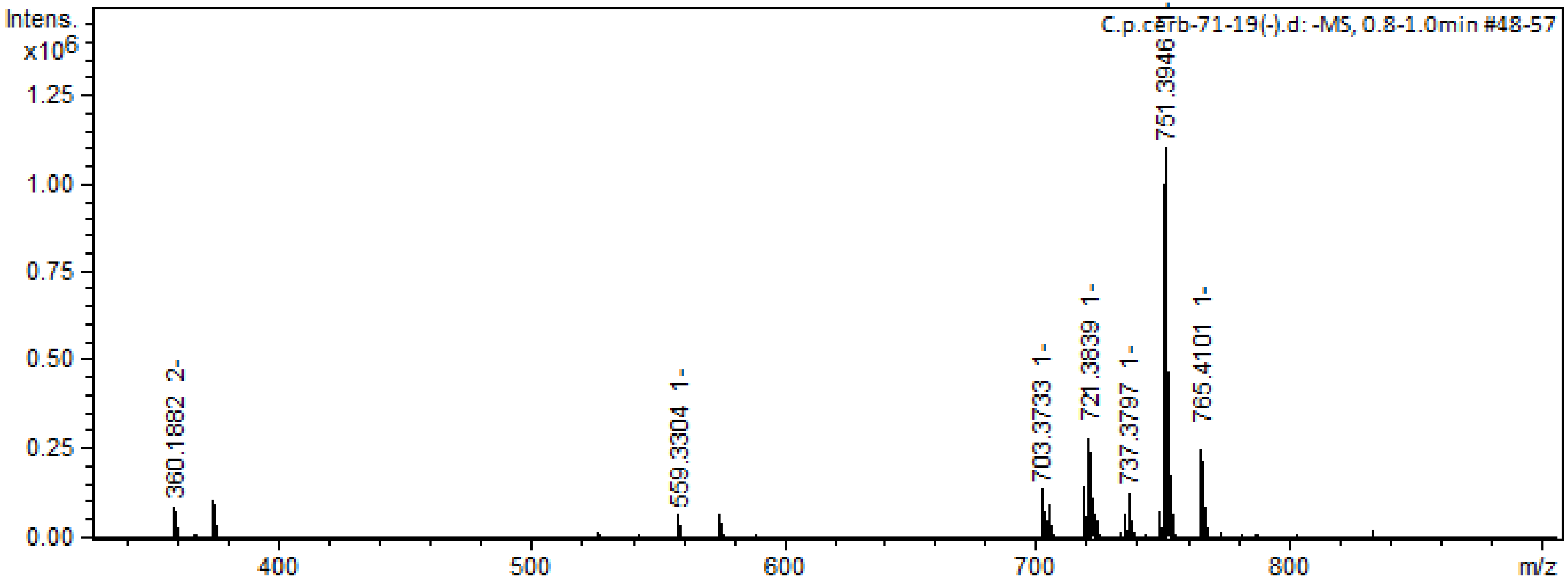

Figure S13. (–)-ESIMS/MS spectrum of ceramasteroside B (**2**).

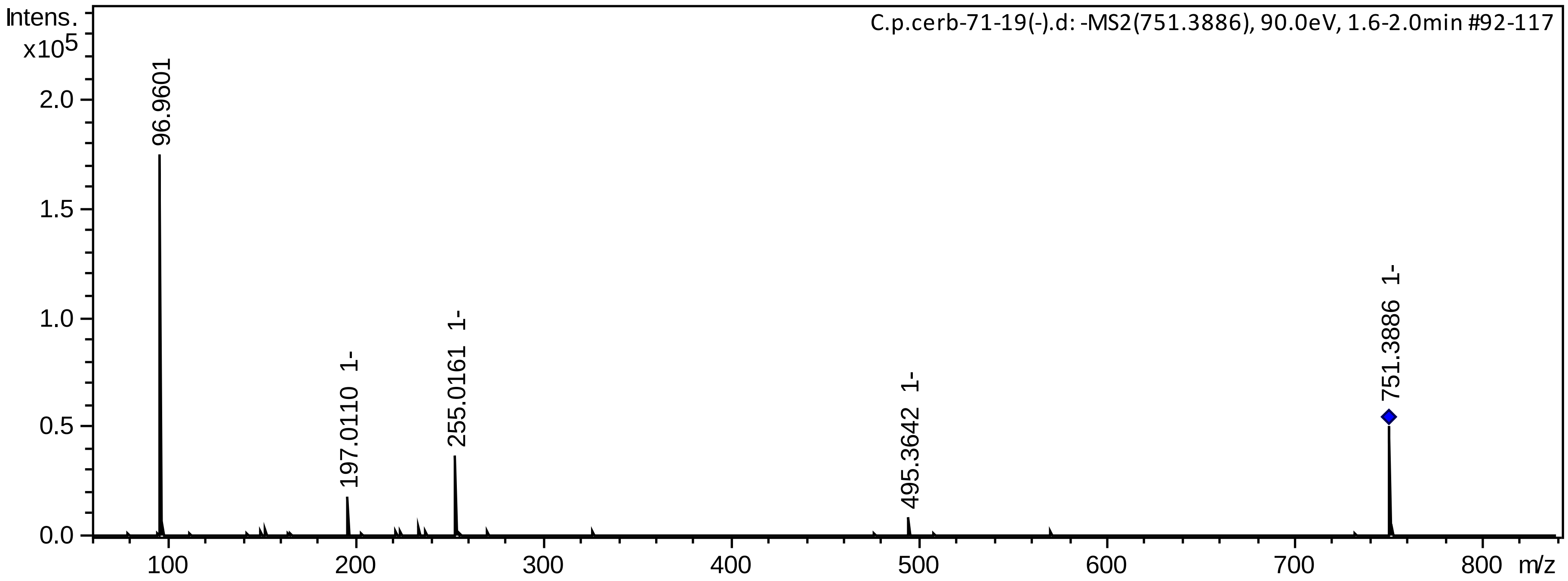

Figure S14. IR spectrum of ceramasteroside B (**2**) in thin layer.

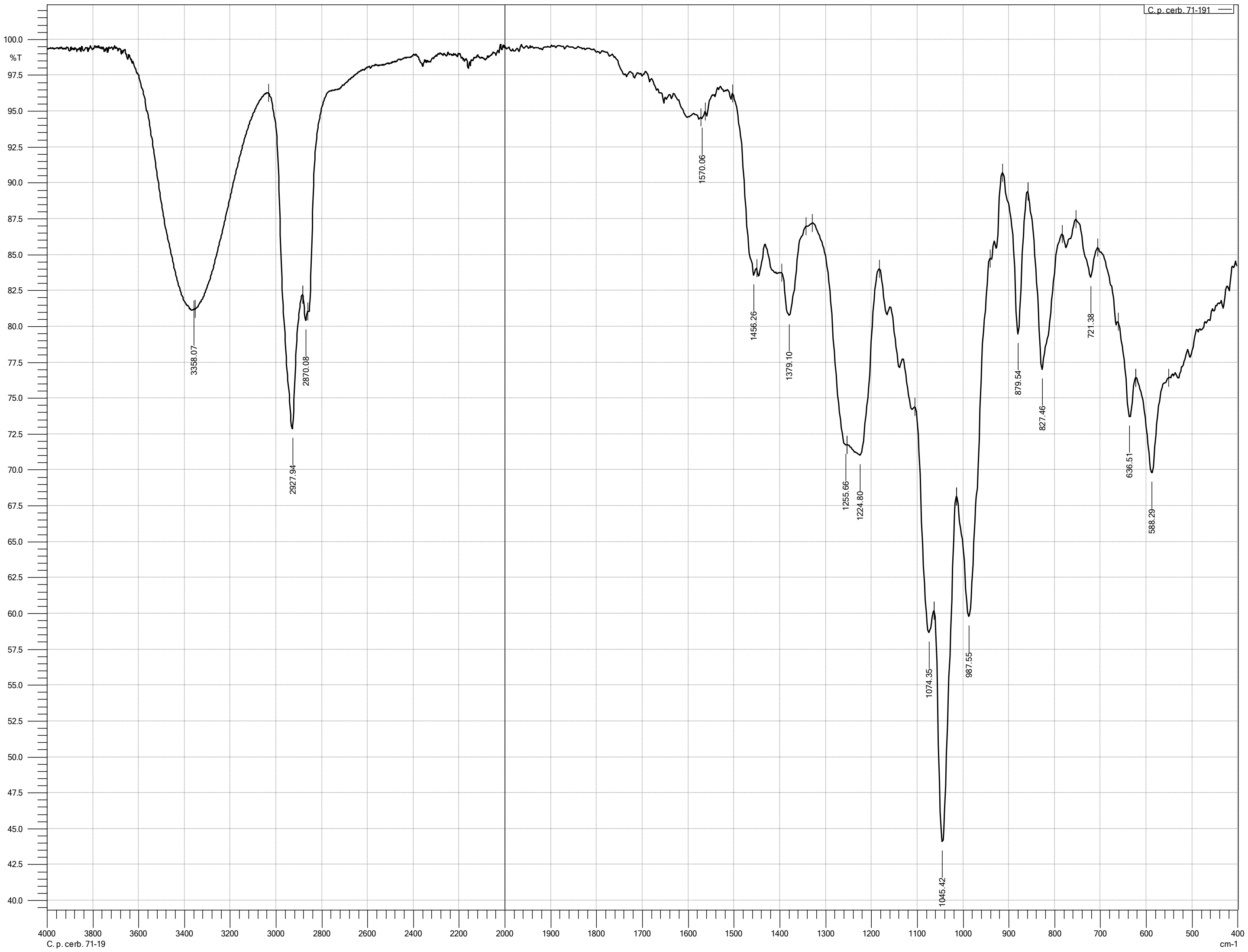

Figure S15. ^{1}H NMR spectrum of ceramasteroside B (**2**) in CD_3OD.

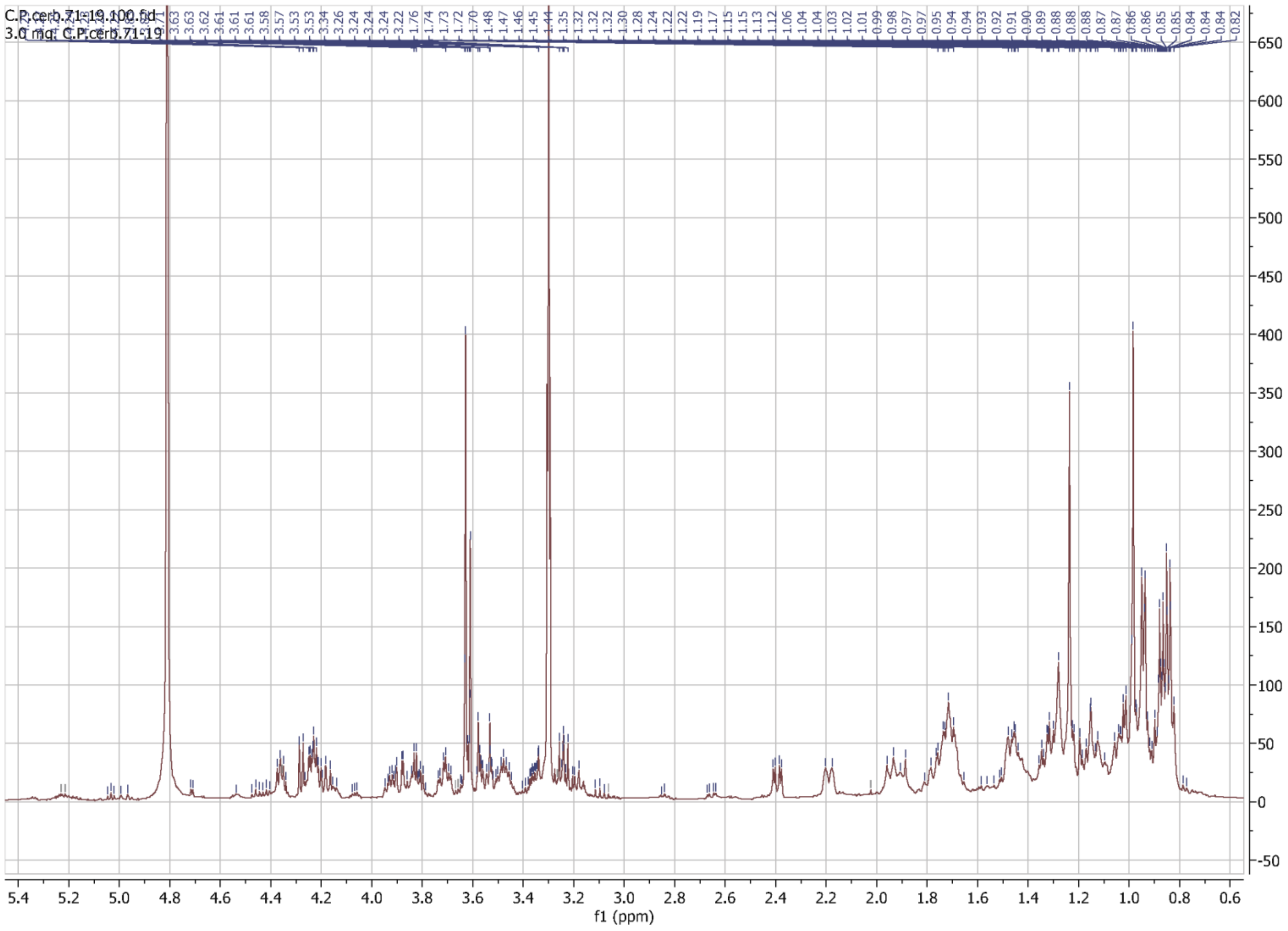

Figure S16. ^{13}C NMR spectrum of ceramasteroside B (**2**) in CD_3OD.

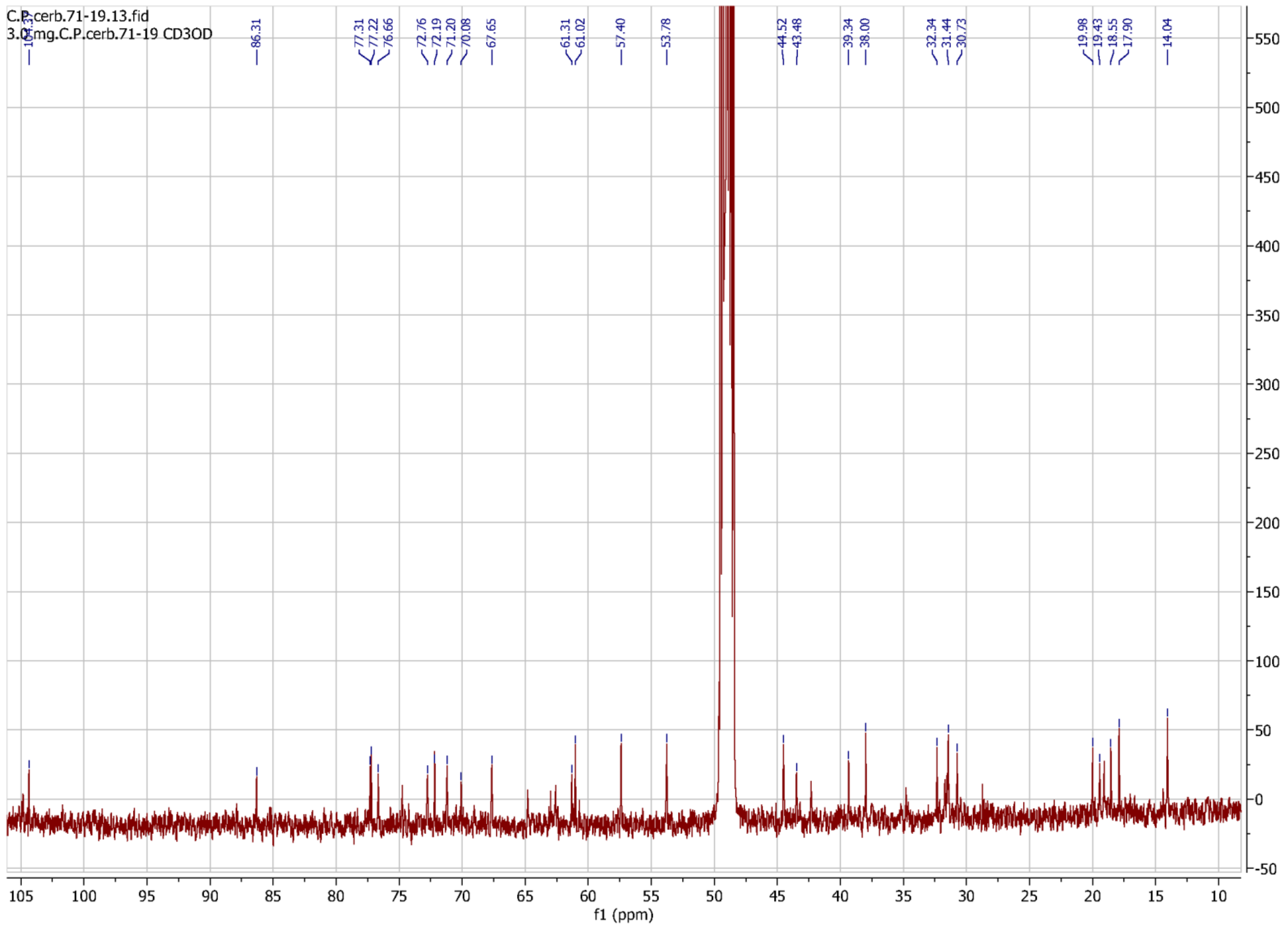

Figure S17. ^{1}H-^{1}H COSY spectrum of ceramasteroside B (**2**) in CD_3OD.

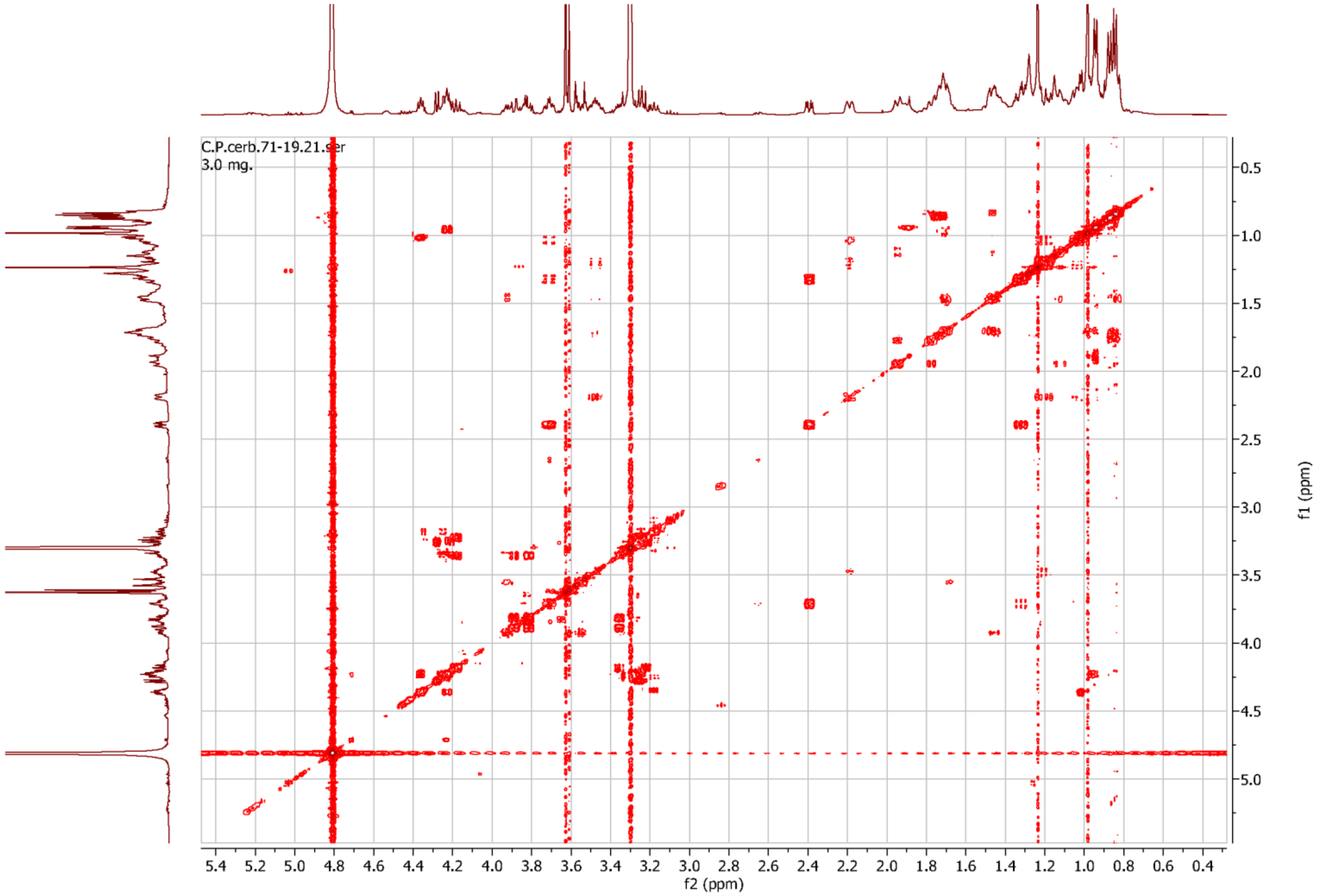

Figure S18. HSQC spectrum of ceramasteroside B (**2**) in CD_3OD.

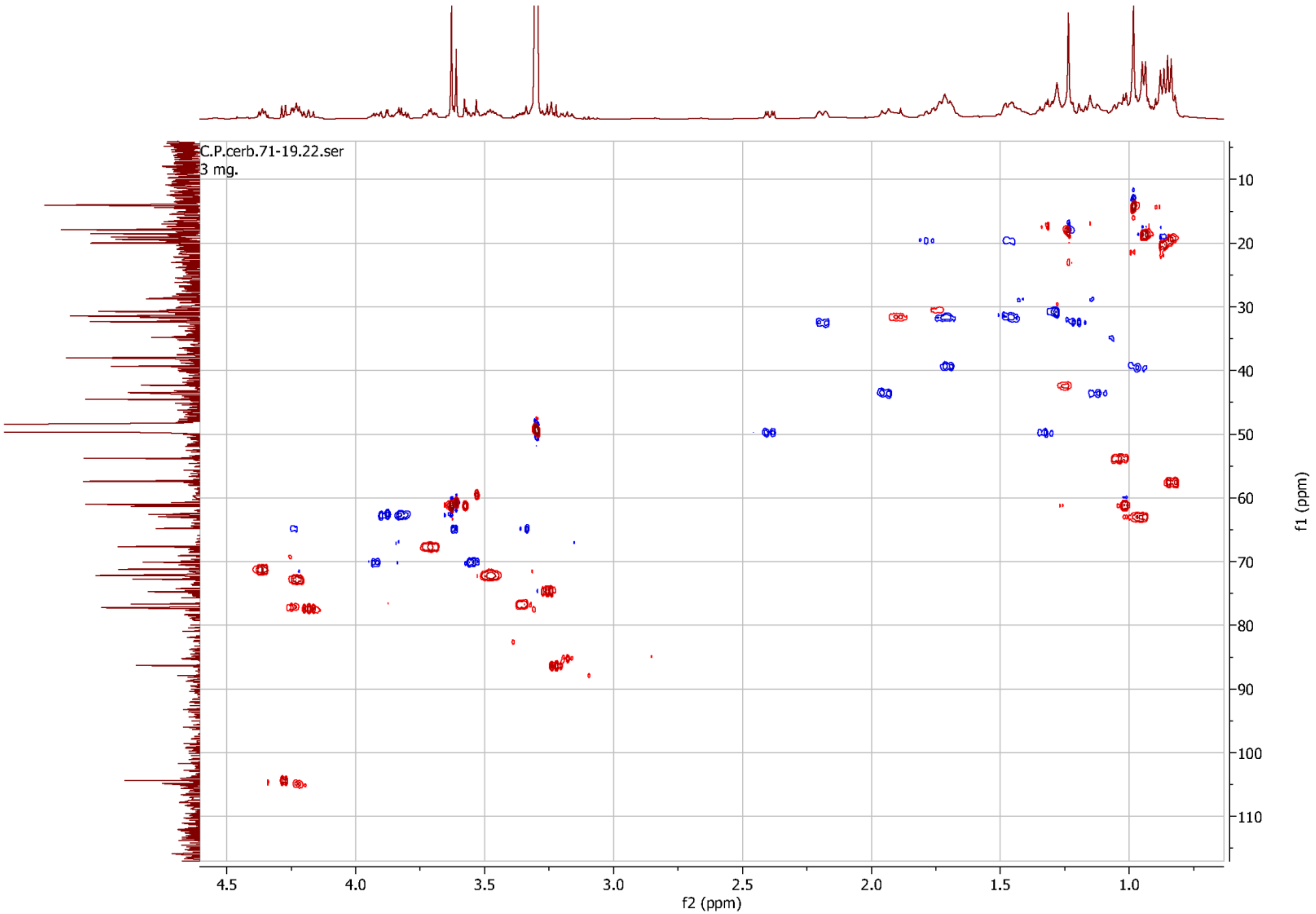

Figure S19. HMBC spectrum of ceramasteroside B (**2**) in CD_3OD.

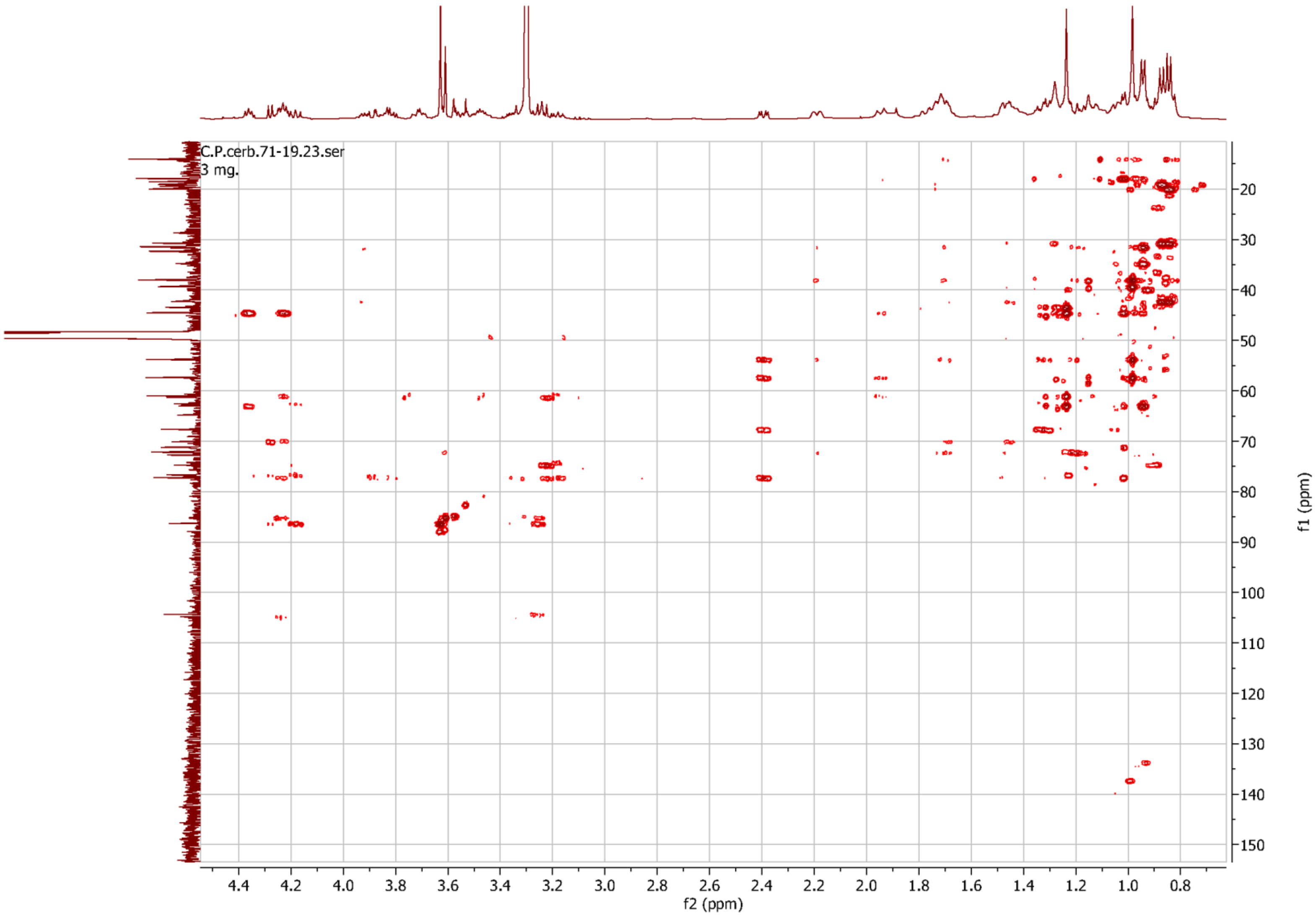

Figure S20. ROESY spectrum of ceramasteroside B (**2**) in CD_3OD.

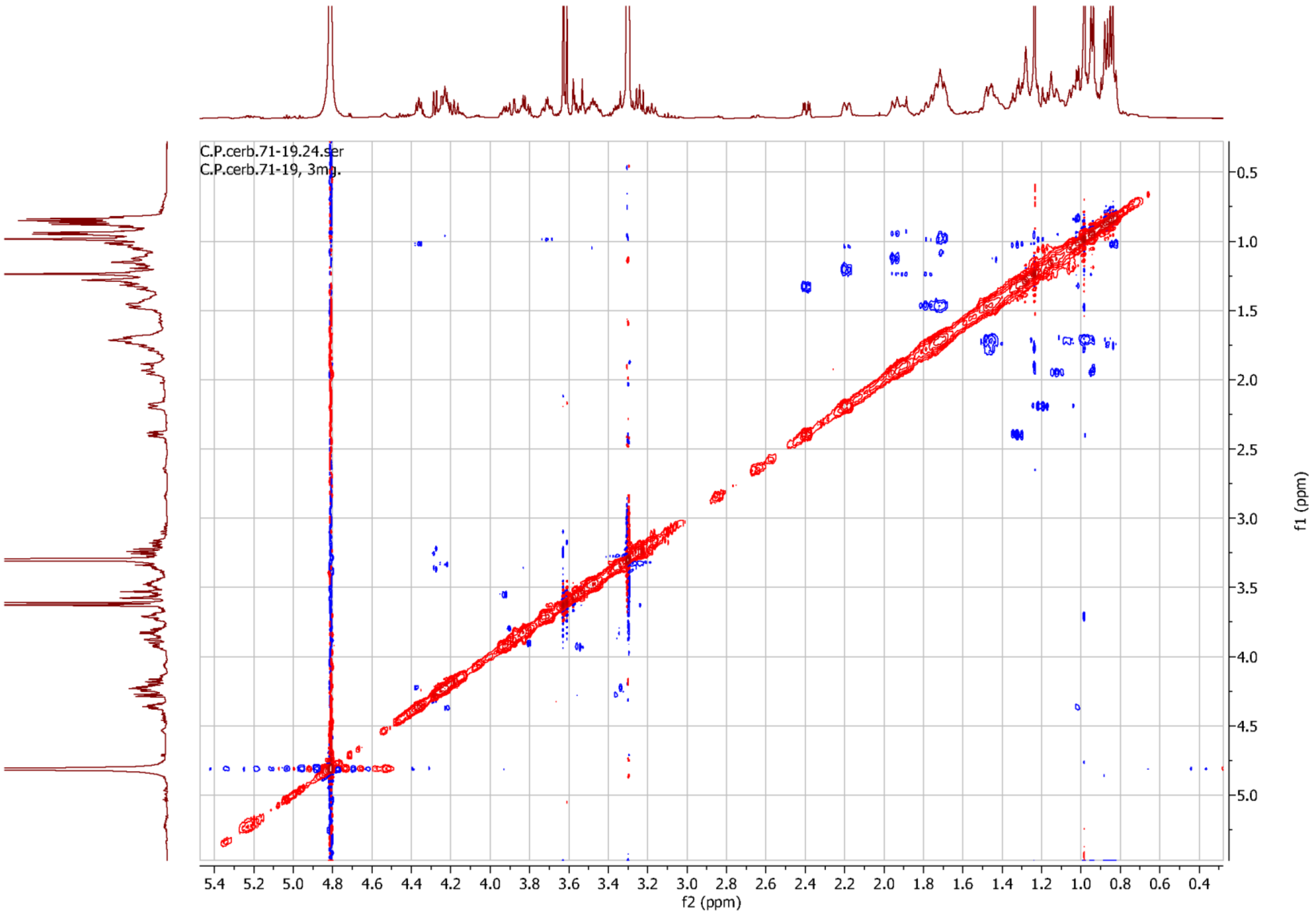

Figure S21. (+)-HRESIMS spectrum ceramasteroside D (**3**).

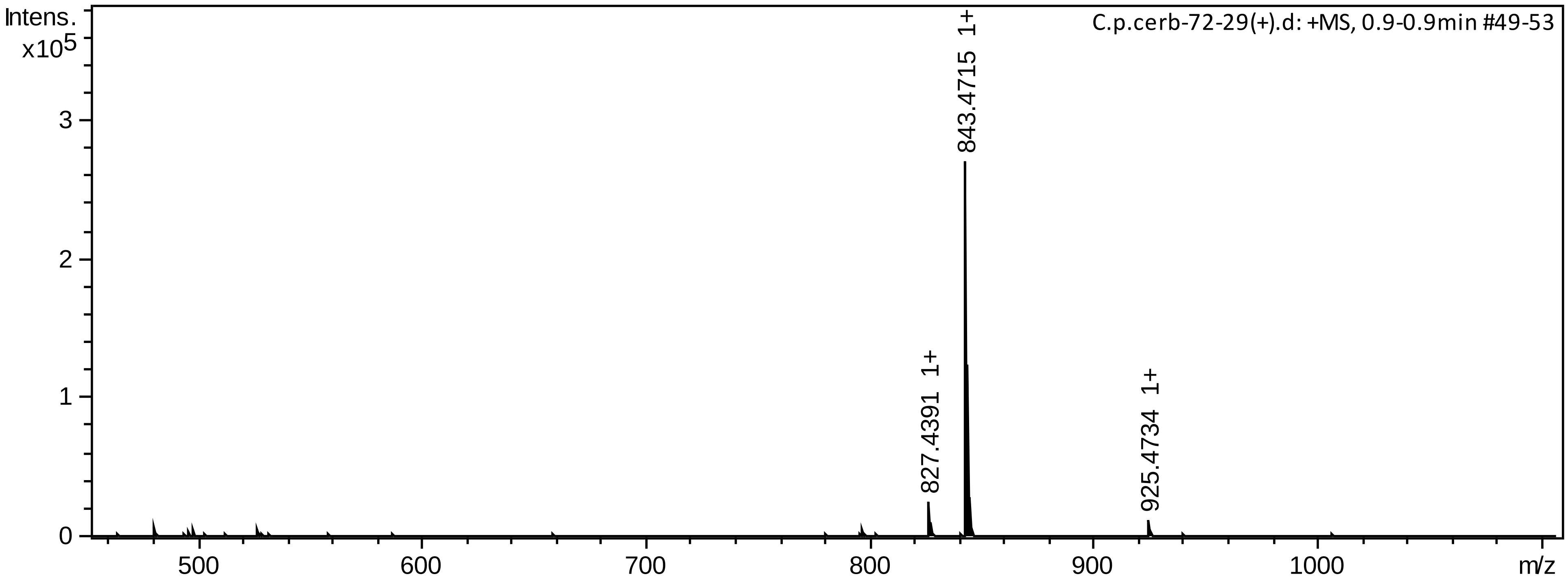

Figure S22. (–)-HRESIMS spectrum of ceramasteroside D (**3**).

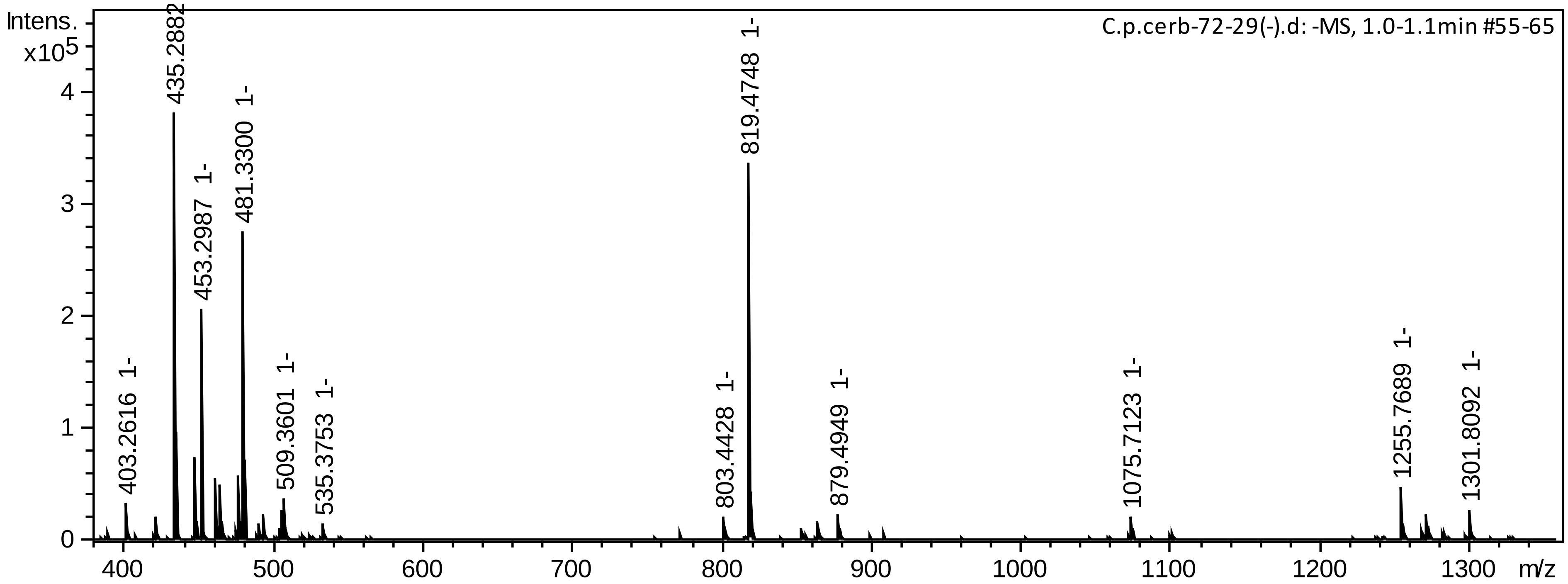

Figure S23. (–)-ESIMS/MS spectrum of ceramasteroside D (**3**).

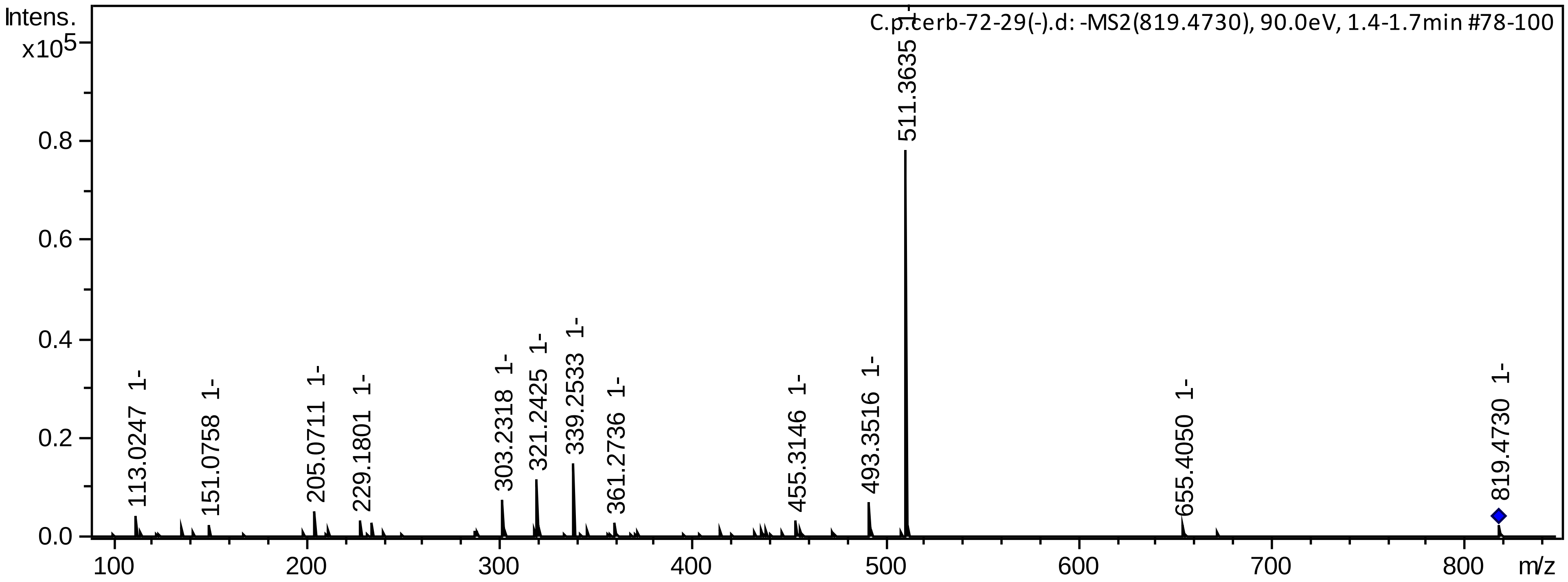

Figure S24. IR spectrum of ceramasteroside D (**3**) in thin layer.

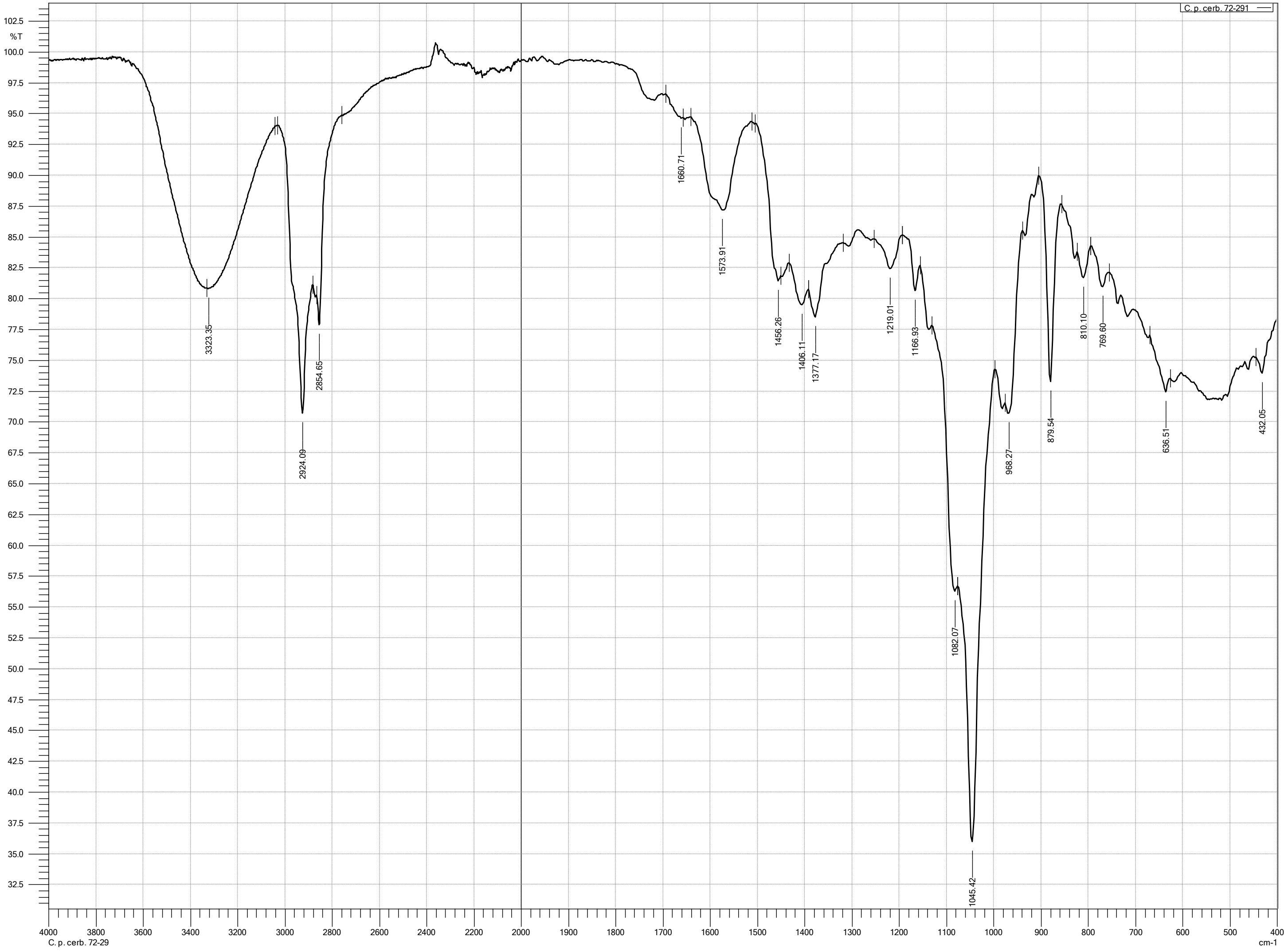

Figure S25. ^{1}H NMR spectrum of ceramasteroside D (**3**) in CD_3OD.

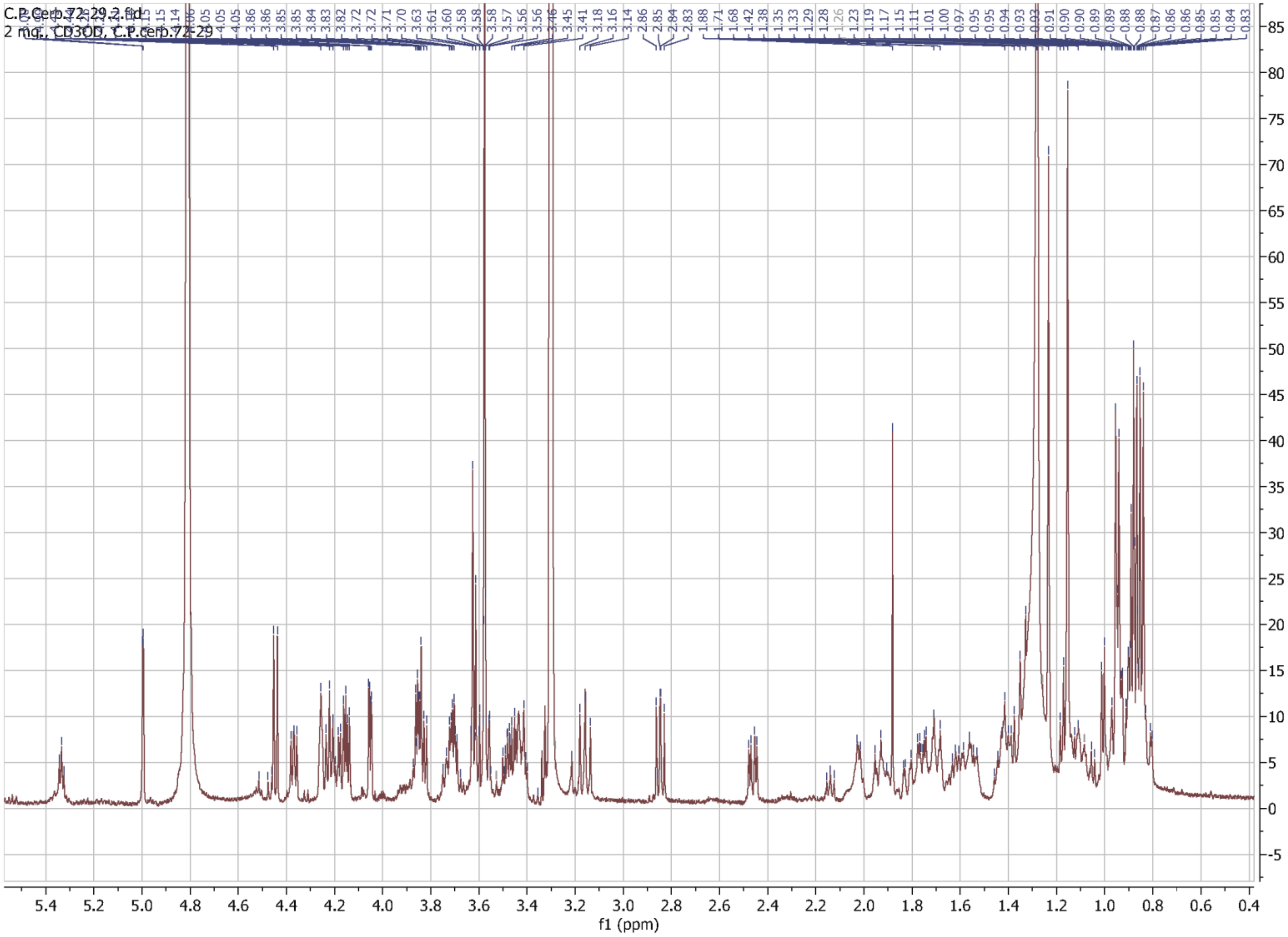

Figure S26. ^{13}C NMR spectrum of ceramasteroside D (**3**) in CD_3OD.

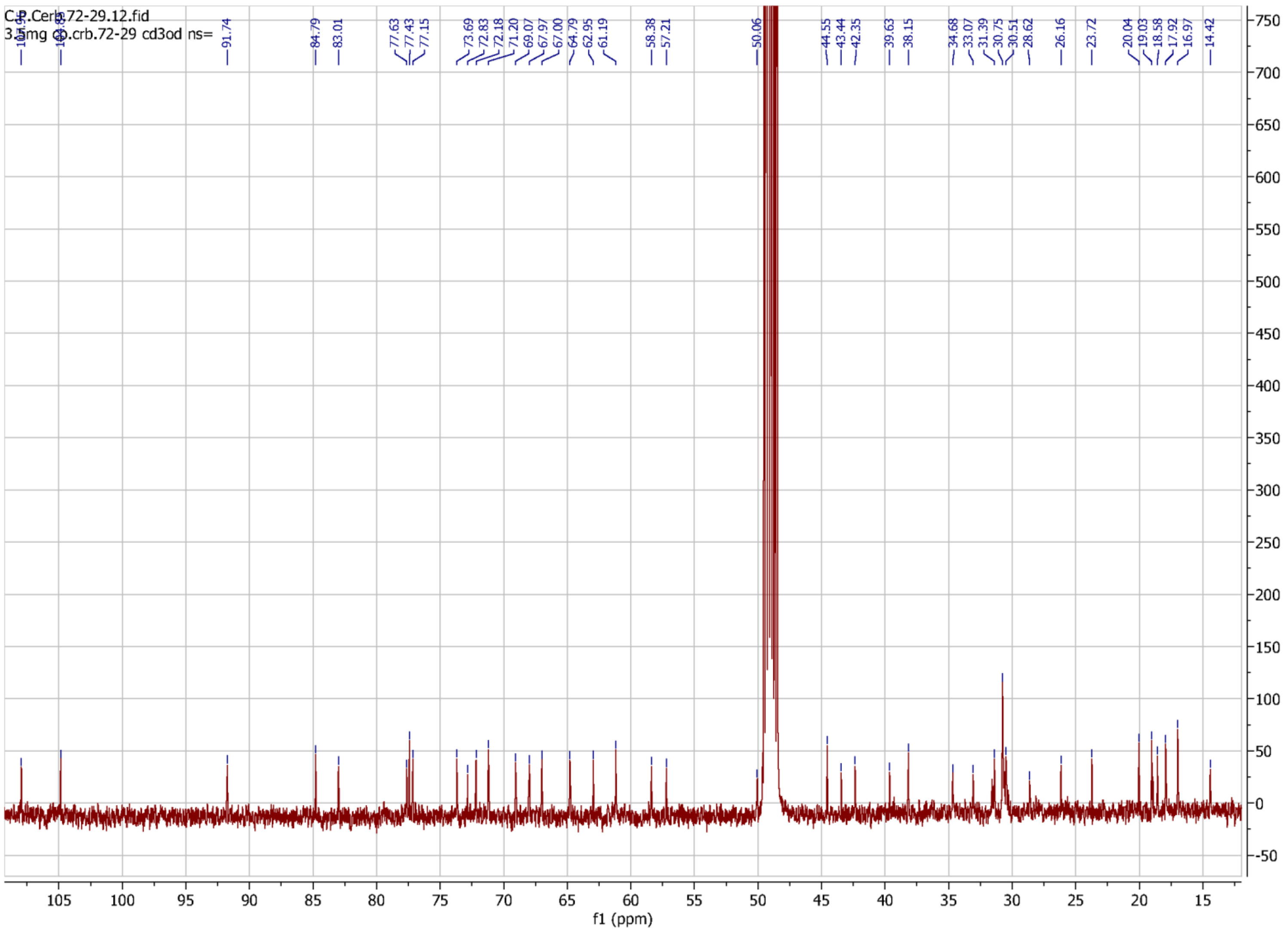

Figure S27. ^{1}H-^{1}H COSY spectrum of ceramasteroside D (**3**) in CD_3OD.

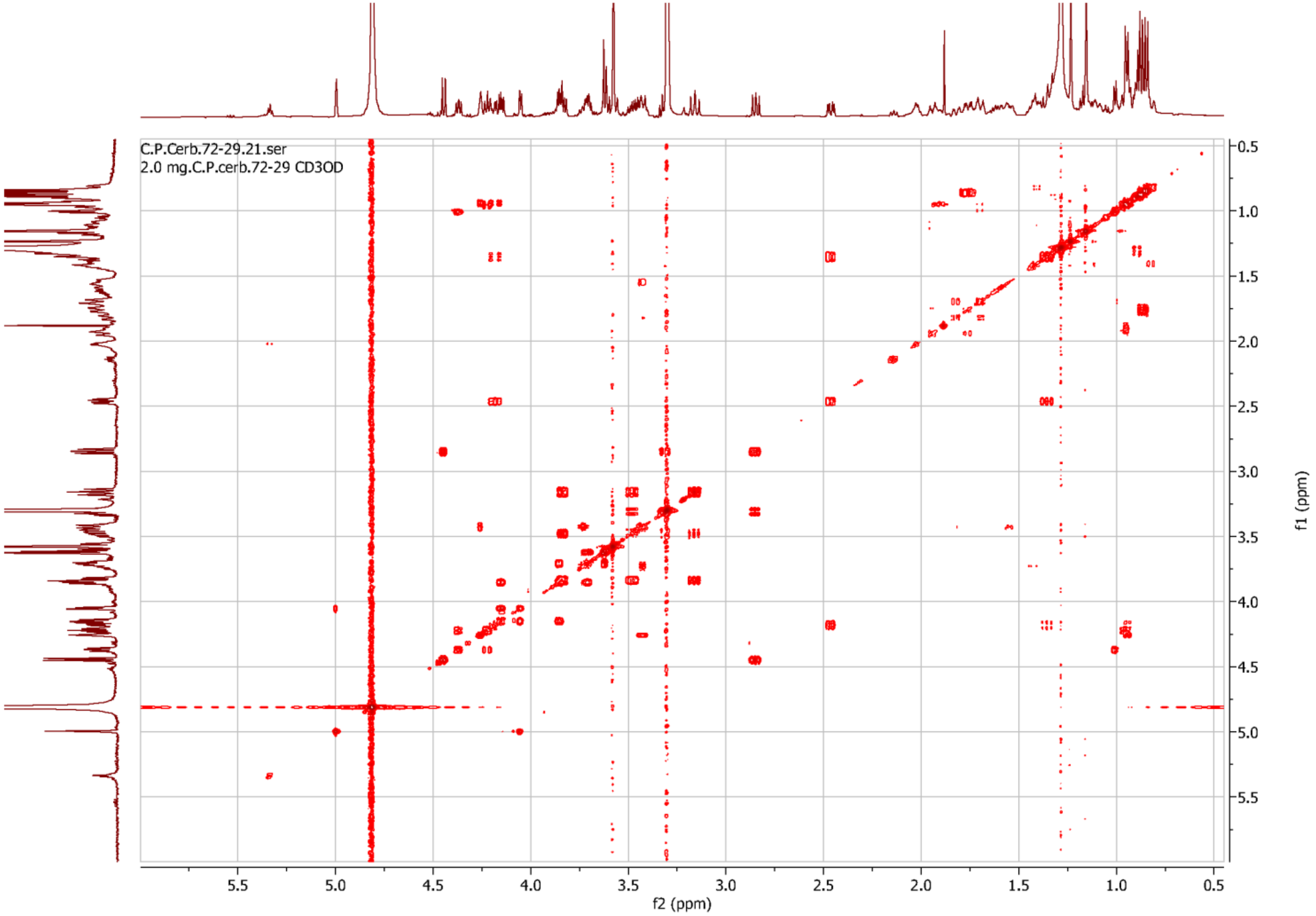

Figure S28. HSQC spectrum of ceramasteroside D (**3**) in CD_3OD.

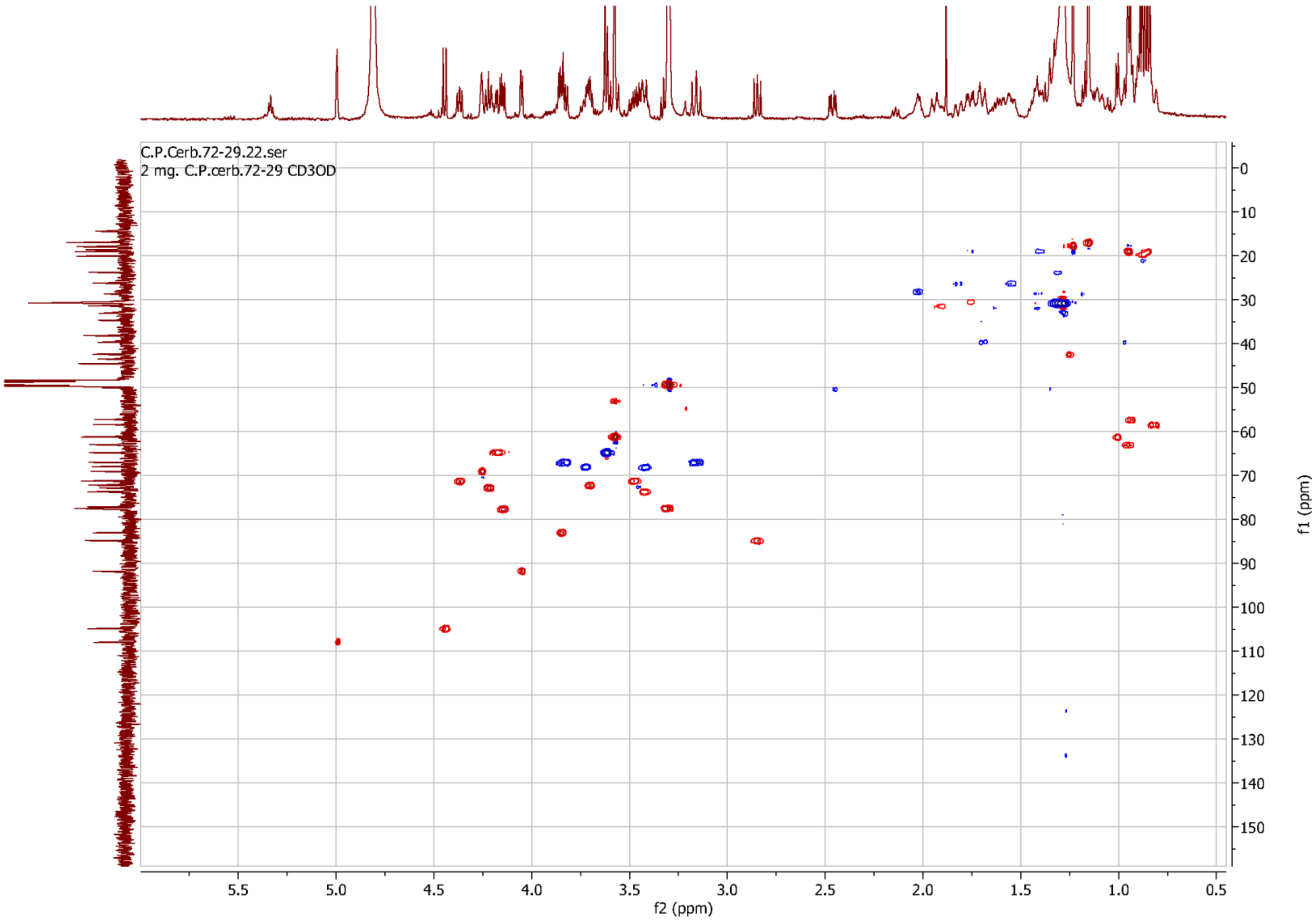

Figure S29. HMBC spectrum of ceramasteroside D (**3**) in CD_3OD.

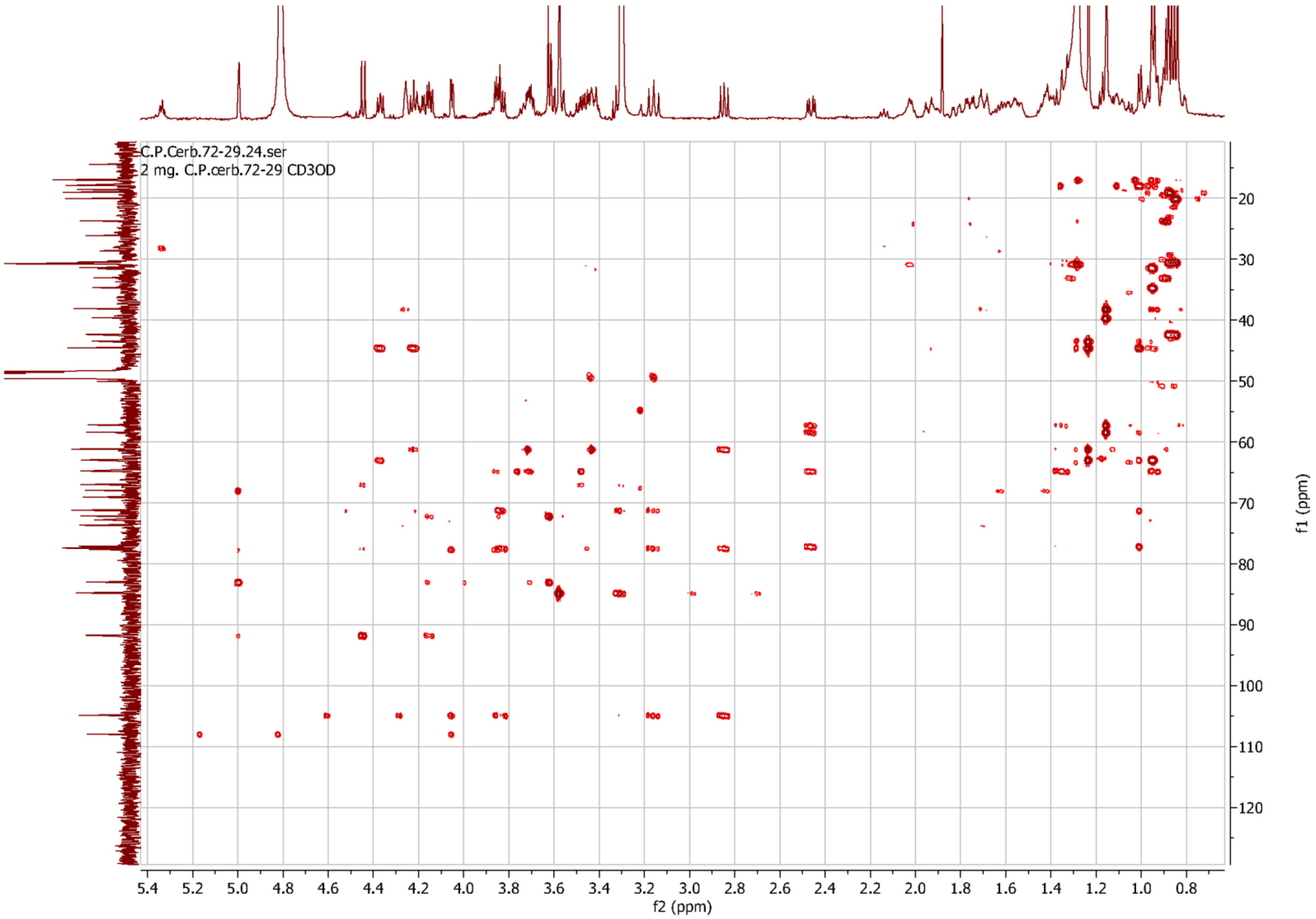

Figure S30. ROESY spectrum of ceramasteroside D (**3**) in CD_3OD.

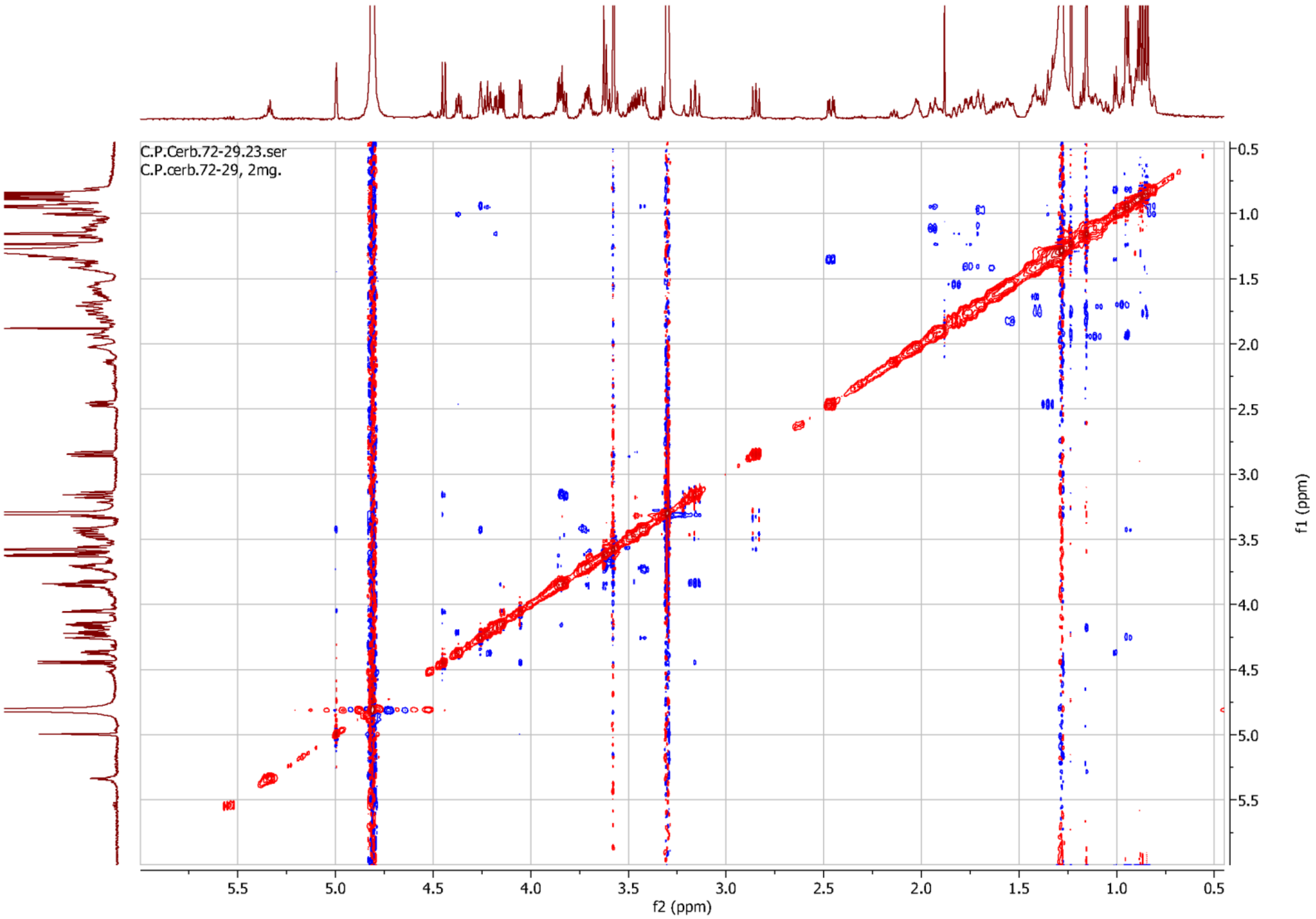

Figure S31. (+)-HRESIMS spectrum of ceramasteroside E (**4**).

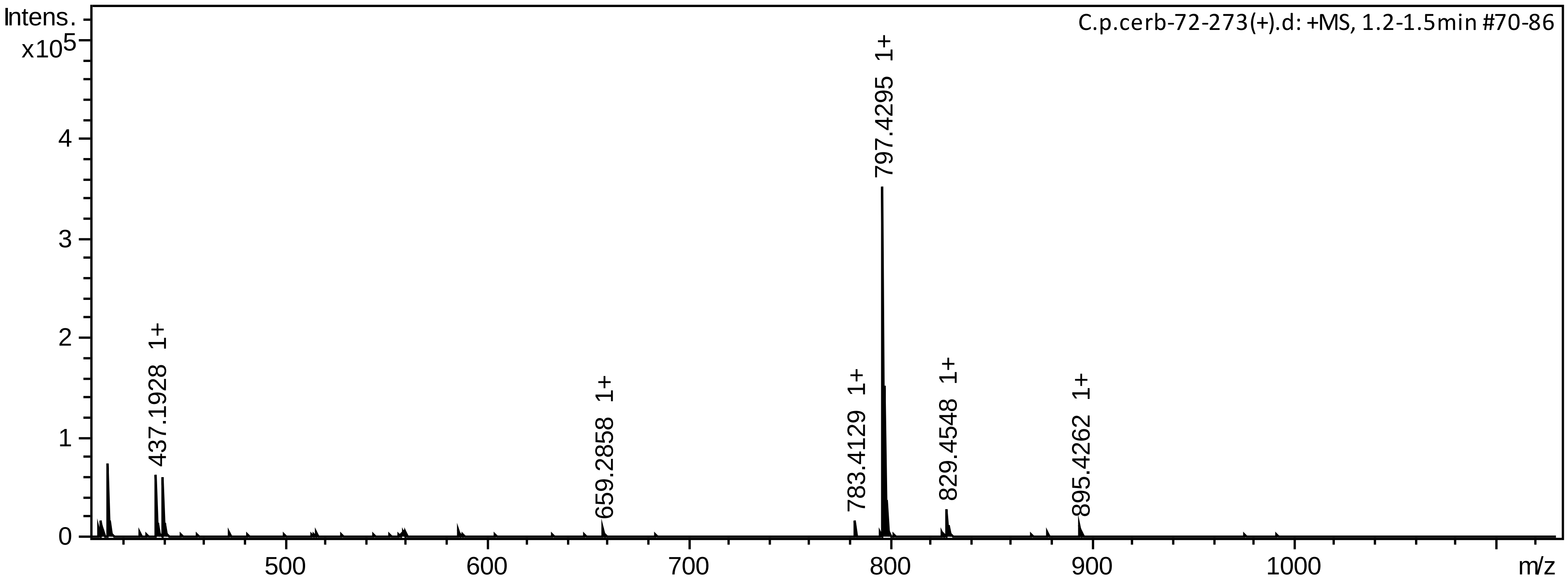

Figure S32. (–)-HRESIMS spectrum of ceramasteroside E (**4**).

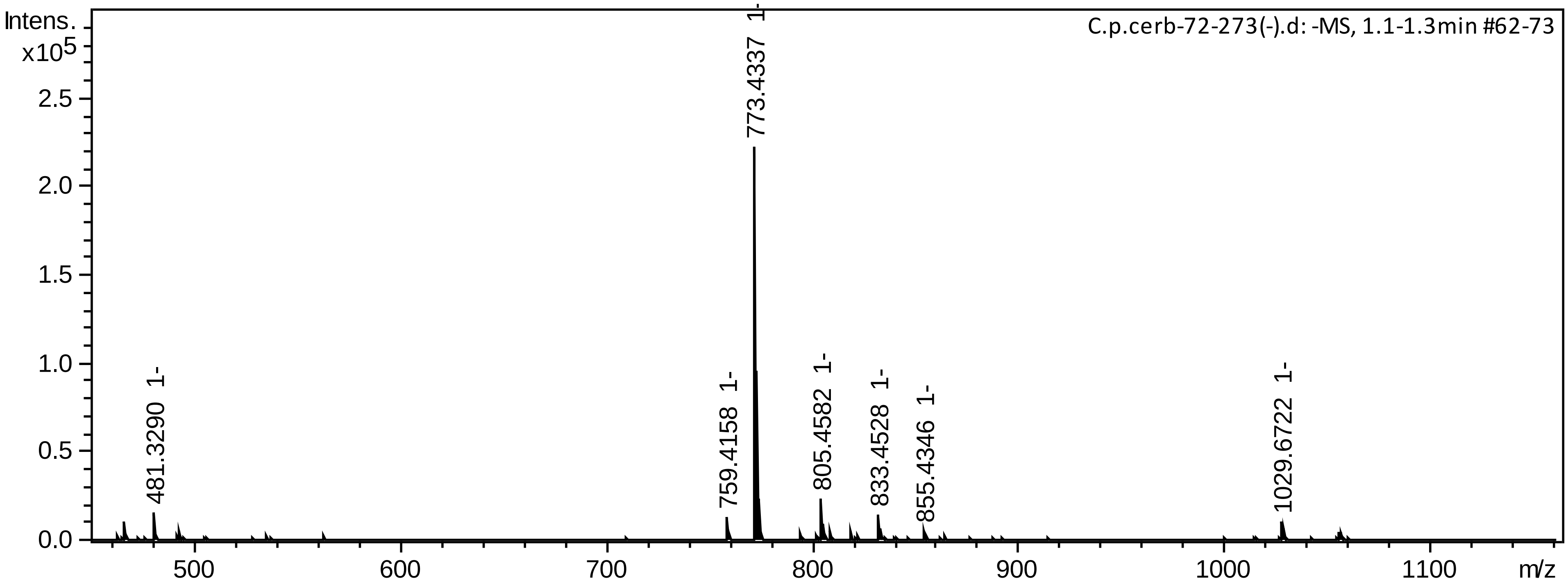

Figure S33. (–)-ESIMS/MS spectrum of ceramasteroside E (**4**).

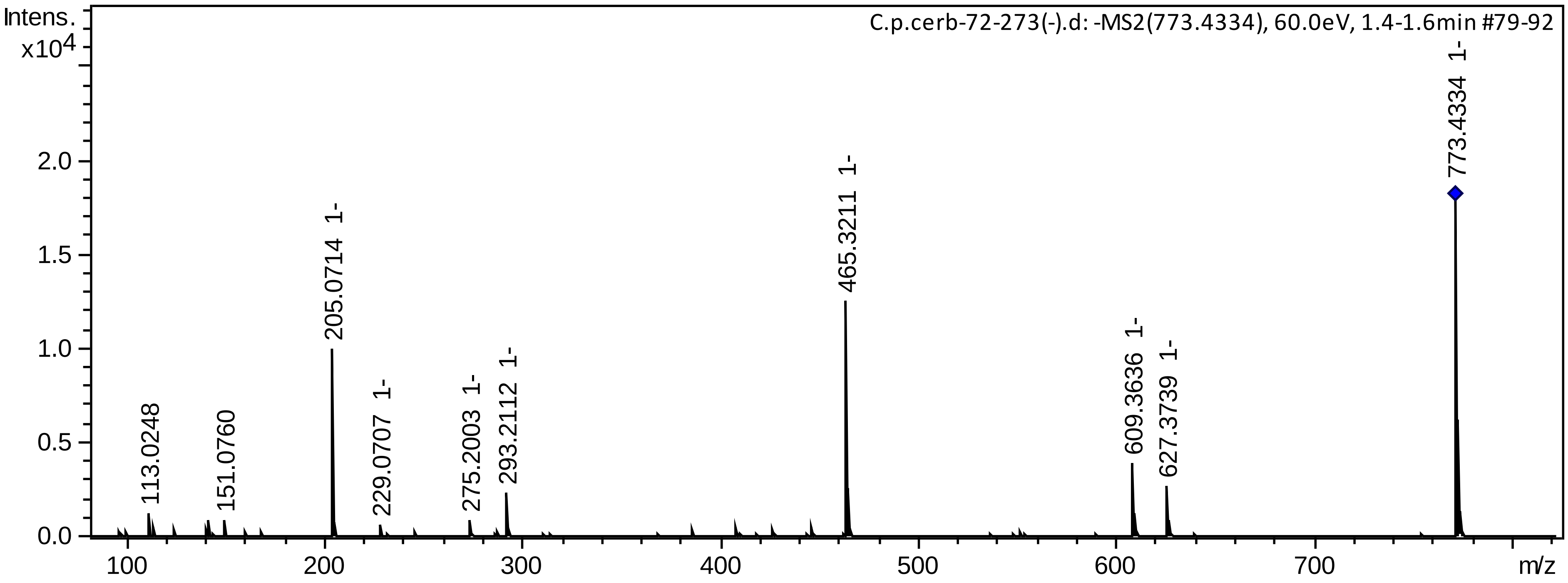

Figure S34. IR spectrum of ceramasteroside E (**4**) in thin layer.

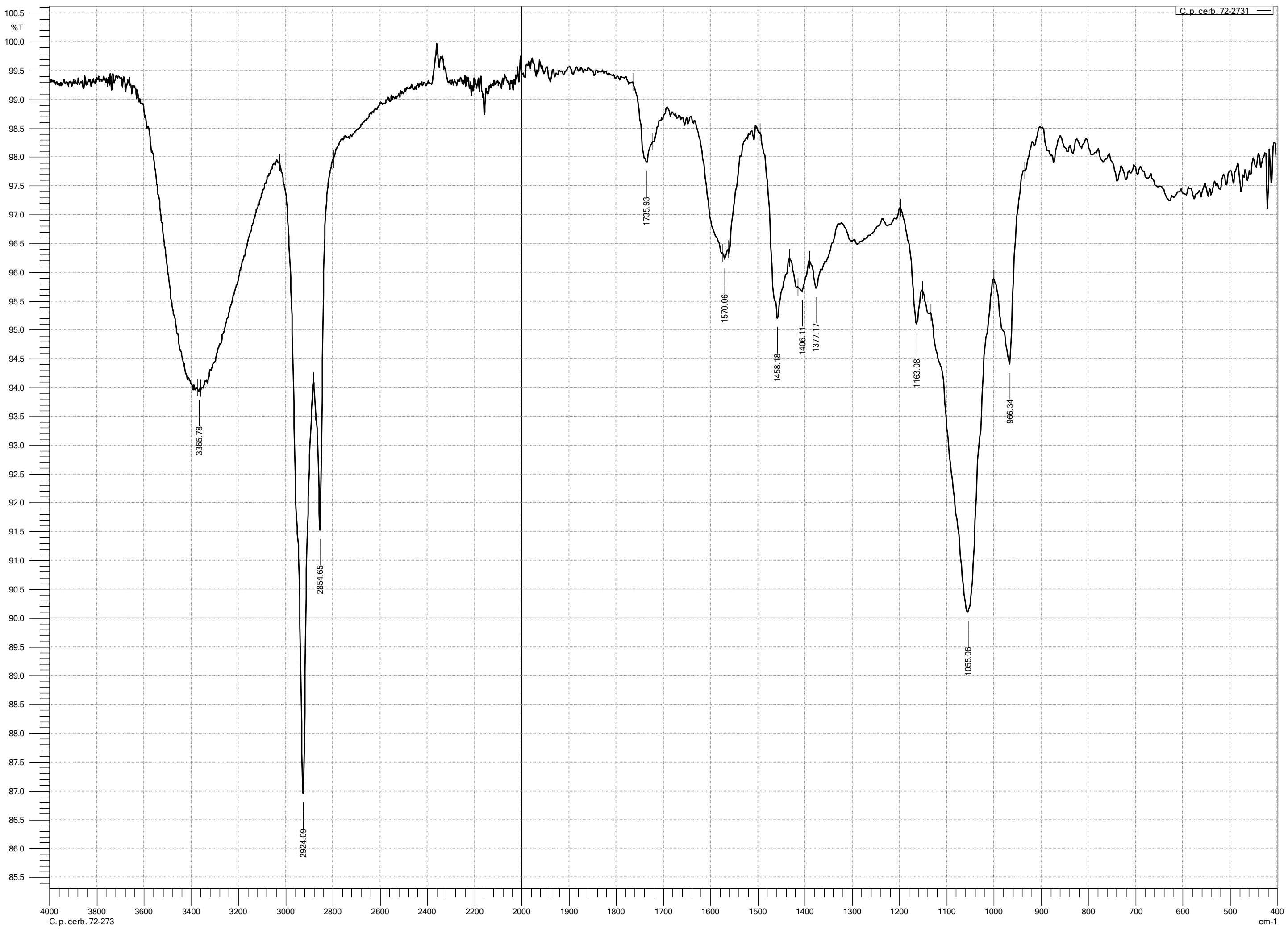

Figure S35. ^{1}H NMR spectrum of ceramasteroside E (**4**) in CD_3OD.

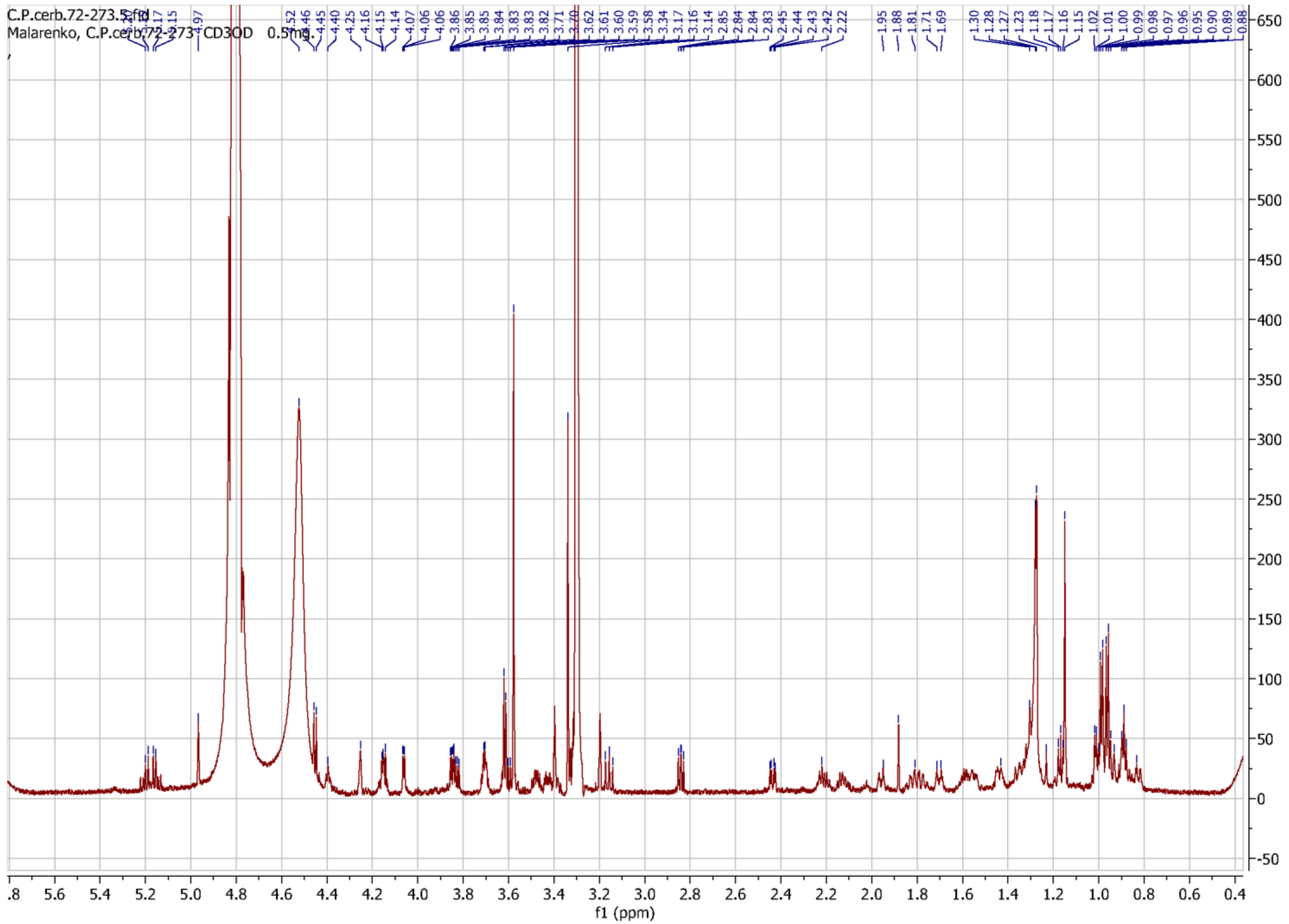

Figure S36. ^{13}C NMR spectrum of ceramasteroside E (**4**) in CD_3OD.

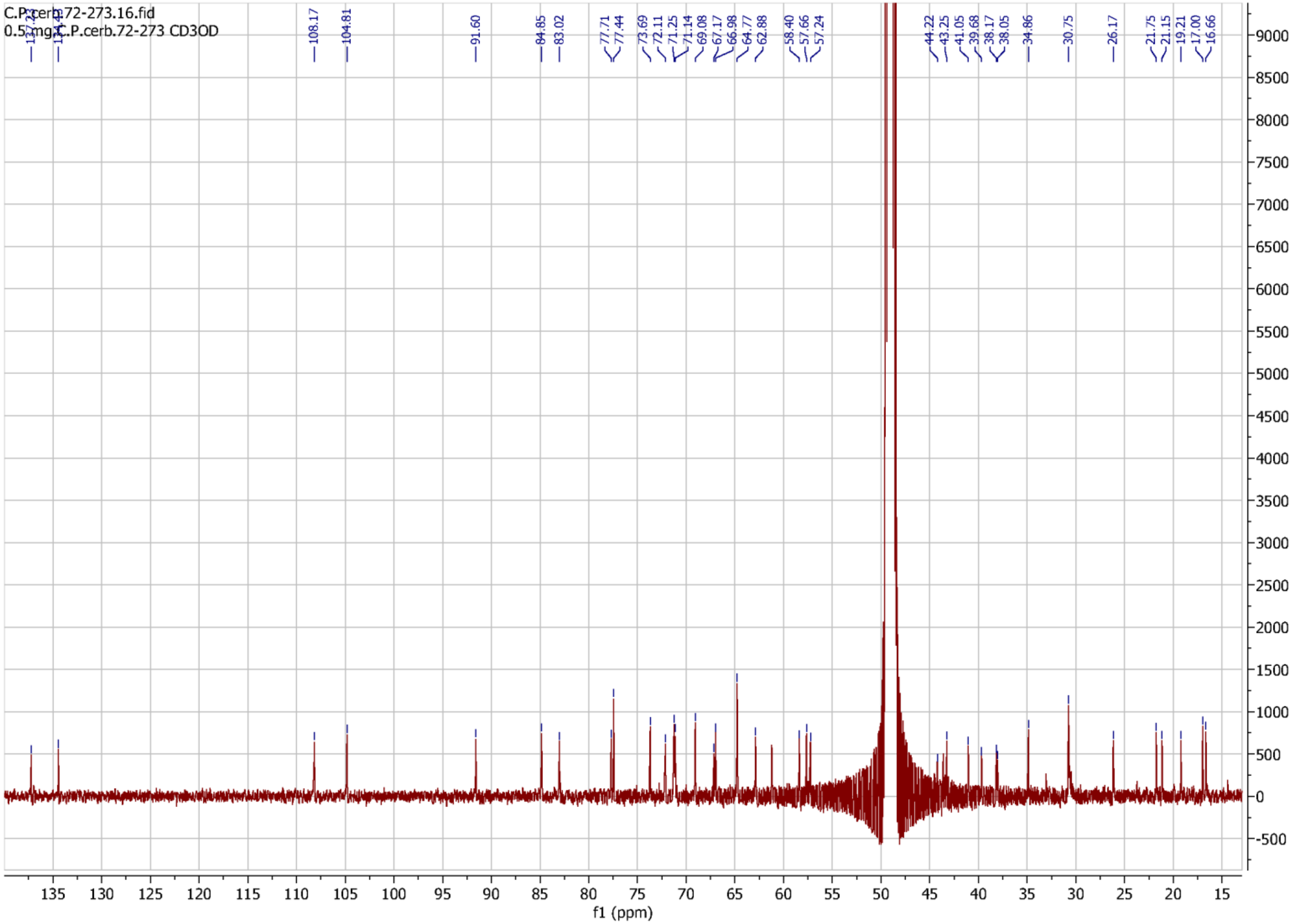

Figure S37. ^{1}H-^{1}H COSY spectrum of ceramasteroside E (**4**) in CD_3OD.

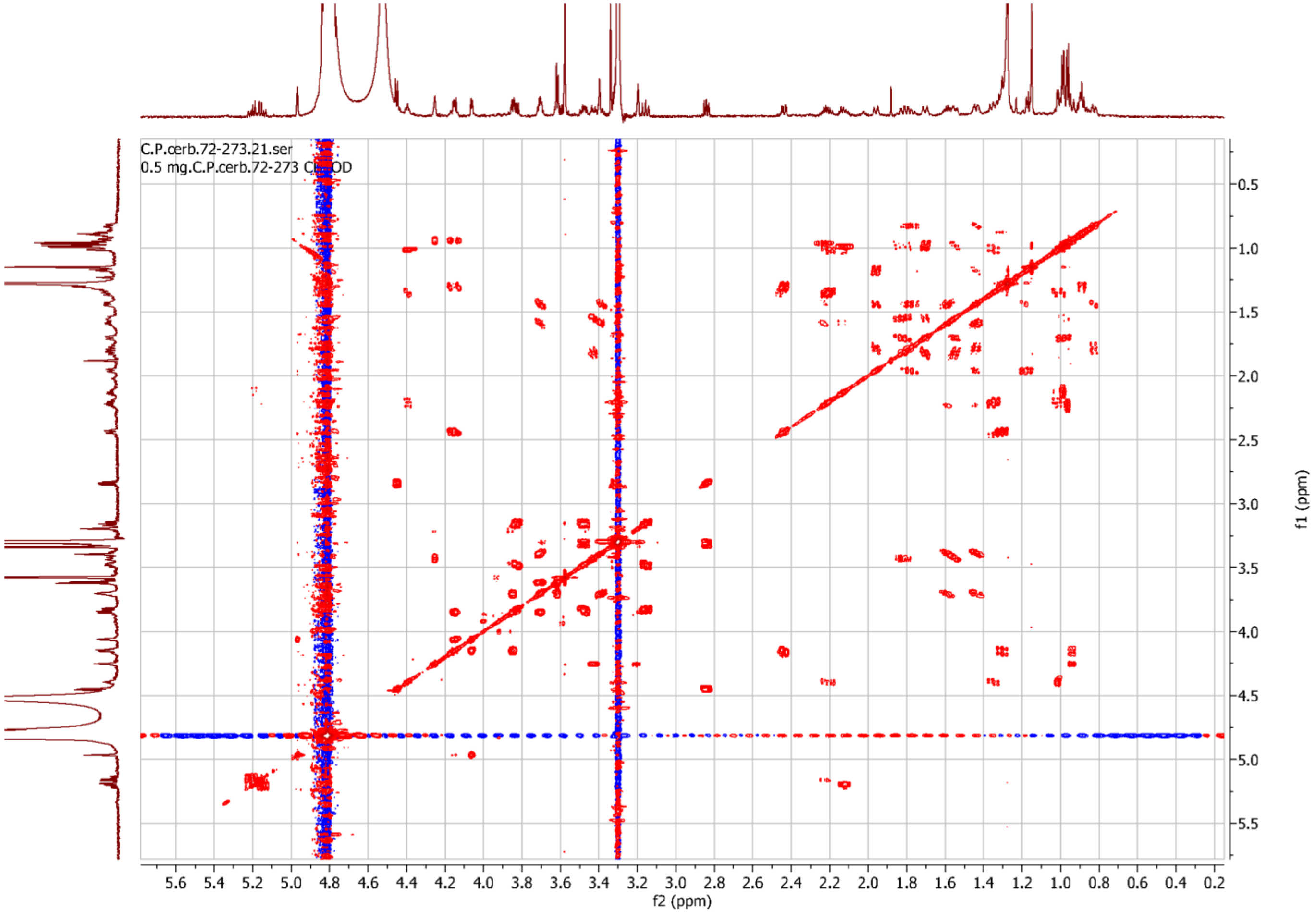

Figure S38. HSQC spectrum of ceramasteroside E (**4**) in CD_3OD.

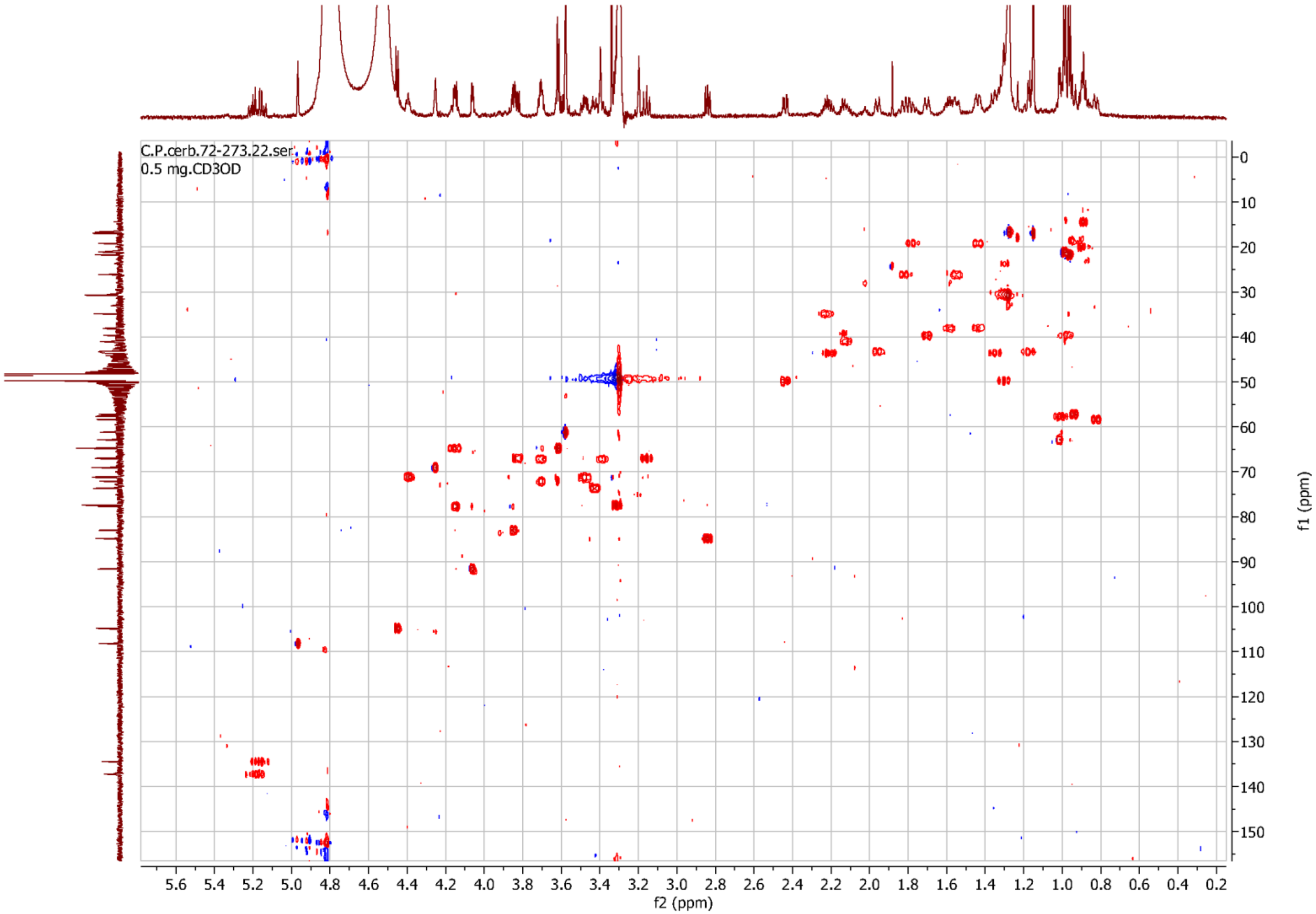

Figure S39. HMBC spectrum of ceramasteroside E (**4**) in CD_3OD.

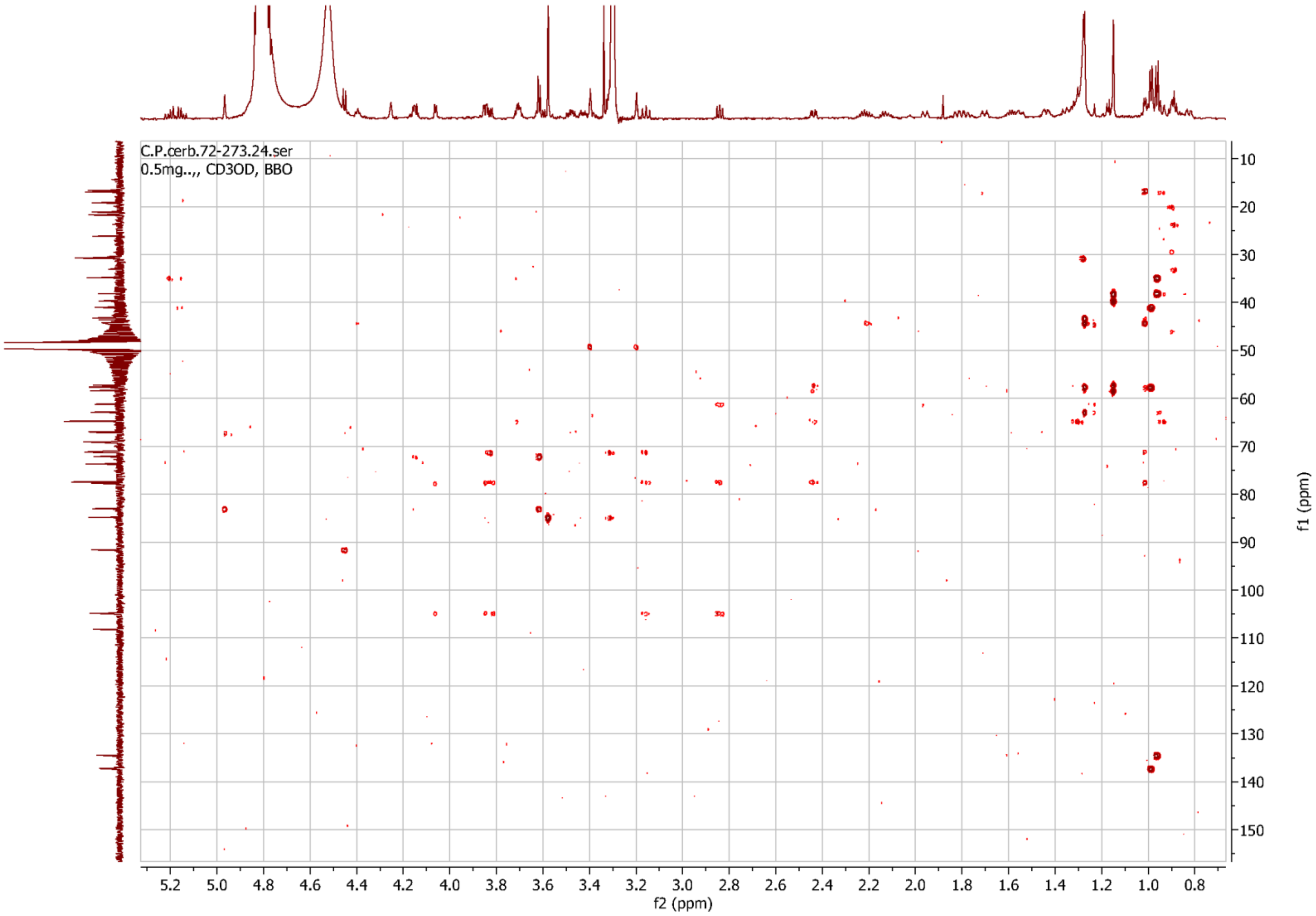

Figure S40. ROESY spectrum of ceramasteroside E (**4**) in CD_3OD.

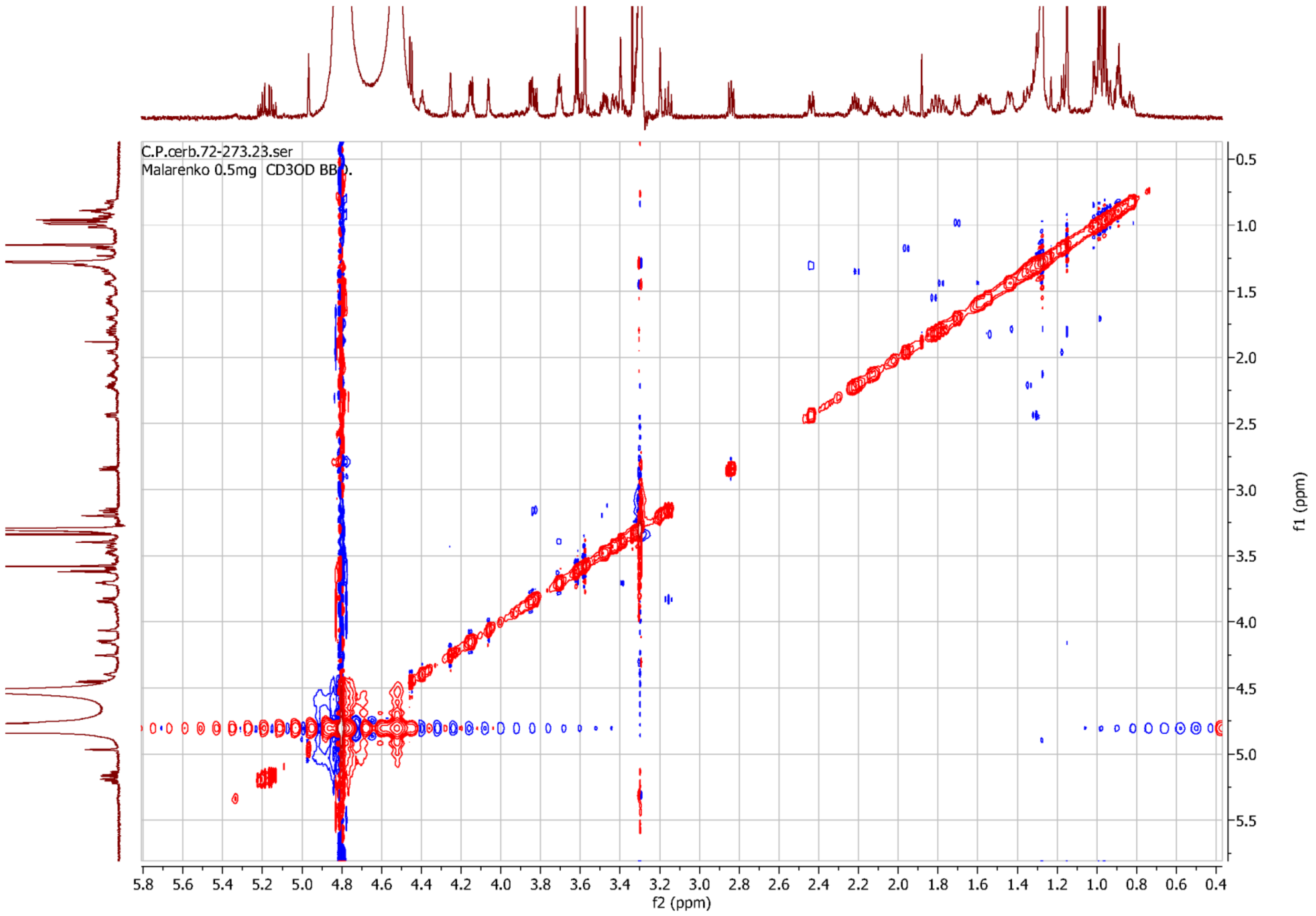

Supplement: Supplementary file 1 [file marinedrugs-22-00508-s001.zip › marinedrugs-3277730-supplementary.pdf]
